# Matrix Metalloproteinase Gene Polymorphisms Are Associated with Breast Cancer in the Caucasian Women of Russia

**DOI:** 10.3390/ijms232012638

**Published:** 2022-10-20

**Authors:** Nadezhda Pavlova, Sergey Demin, Mikhail Churnosov, Evgeny Reshetnikov, Inna Aristova, Maria Churnosova, Irina Ponomarenko

**Affiliations:** Department of Medical Biological Disciplines, Belgorod State National Research University, 308015 Belgorod, Russia

**Keywords:** *MMP*, breast cancer, SNP, association

## Abstract

We conducted this study to explore the association between matrix metalloproteinase (*MMP*) gene polymorphisms and breast cancer (BC) risk in the Caucasian women of Russia. In total, 358 affected (BC) and 746 unaffected (cancer-free) women were included in this case-control retrospective study. From BC-related genes in previous studies, ten single nucleotide polymorphisms (SNPs) in five *MMP* genes (*MMP1*, *2*, *3*, *8*, *9*) were genotyped. The BC risk was calculated by logistic regression (to evaluate the SNPs’ independent effects) and model-based multifactor dimensionality reduction (MB-MDR) (to identify SNP–SNP interactions) methods. The allelic variants’ distribution of c.836 A > G (rs17576) and c. 1721 C > G (rs2250889) *MMP9* was significantly different between BC and cancer-free women: for G minor alleles, these SNPs manifested disorder protective effects (OR 0.82 and OR 0.67–0.71, respectively, *p*_perm_ ≤ 0.035). Eleven haplotypes of six SNPs *MMP9* were involved in BC risk (nine haplotypes) and protective (two haplotypes) effects. All 10 SNPs of the *MMP* genes examined were associated with BC within the 13 SNP–SNP interaction simulated models, with a pivotal role of the two-locus (rs17577 × rs3918242) *MMP9* epistatic interaction (defined as 1.81% BC entropy within more than 60% of the genetic models). Under in silico bioinformatics, BC susceptibility *MMP* polymorphic loci are located in functionally active genome regions and impact genes expression and splicing “regulators” in the mammary gland. The biological pathways of BC *MMP* candidate genes are mainly realized due to metalloendopeptidase activity and extracellular matrix organization (structure, disassembly, metabolic process, etc.). In conclusion, our data show that *MMP* gene polymorphisms are related to BC susceptibility in the Caucasian women of Russia.

## 1. Introduction

BC is a malignant breast tumor with epithelial origin [1]. According to world statistics (material of the International Agency for Research on Cancer), currently, more than 2 million new cases of BC are detected annually among the world population [2] (http://canscreen5.iarc.fr, accessed on 17 September 2022). Among all cases of cancer (19.3 million new tumor cases are registered annually in the world), BC is the most frequent (11.7%) [2]. Among the female population, the proportion of BC among all oncological diseases is 24.5%, and regarding global female mortality, the proportion of BC is 15.5% [3]. The problem of BC is no less significant for Russia. According to official statistics, the incidence rate is 82.8 per 100,000 women [4]. BC ranks first both in terms of cancer incidence in Russian women (20.9%) and the cause of female death from malignant tumors (16.2%) [5].

Genetic factors have an essential effect on the susceptibility to BC [6,7,8,9,10]. Genome-wide association studies (GWASs) of BC indicate that more than 180 different single nucleotide polymorphic variants have a connection with the disorder [8]. It should be noted that these polymorphic loci explain only 18% of the disease heritability [9], whereas under modern genetic estimates, the contribution of the hereditary component to BC formation reaches up to 31% [10]. Based on this, it is noted that despite the active study of the genetic foundations of BC, which has been intensively conducted by numerous scientific teams over the past decades, a significant part of the genetic determinants (more than 40%) involved in the occurrence of the disease remain unknown to date, which dictates the need to continue genetic association studies of BC.

Among the “potential” candidate genes for BC, the *MMP* genes are being actively studied [11,12]. The relationship of the expression products of these genes (proteins of the same name) with BC is currently beyond doubt [13,14]. Due to its collagenase activity, the ability to cleave pro-apoptotic factors, mobilize/activate pro-angiogenic factors (growth factors, etc.), and suppress the production of angiogenesis inhibitors (angiostatin, endostatin, etc.), MMP provides degradation of the stromal connective tissue components and the basement membrane, and has “key” importance in the processes of tumor angiogenesis, invasion, and metastasis [13,14]. Increased expression of *MMP* (*MMP1*, *MMP2*, *MMP9*, etc.) in the lesion induces the growth of tumor tissue and initiates its invasion and metastasis [12,15]. There is convincing data on the association of MMP with the survival of BC patients [13].

The relationship of functionally noticeable polymorphisms of *MMP* with BC risk is being actively studied by various scientific teams [12,16,17,18,19,20,21,22,23,24,25,26] etc. Nevertheless, despite the considerable accumulated factual material on this topic, it should be stated that these data often do not agree with each other. In some cases, they are contradictory, and individual loci (for example, c.2003 G>A (rs17577) *MMP9*, p.133 C>T (rs679620) *MMP3* [27,28]) are single and fragmentary. For example, a significant association of c.-1607 2G>1G (rs1799750) *MMP1* with BC was found in five studies, whereas no such association was found in ten studies [12,16,17,18,19]. Contradictory data on the association with BC are available in the previously public literature for the locus c.-1562 C>T (rs3918242) *MMP9*. In some studies, the polymorphic variant T was associated with increased risk of developing the disease [20,21,22]. According to the results of other studies, this locus was not associated with the disorder [16,17,18,23,24,25] or vice versa, the genotype TT was a BC protective factor [26]. The above data dictate the need to continue research on this topic in order to establish the polymorphic loci of *MMP* genes causal for BC in various populations, including the Russian Federation.

Therefore, we conducted this study to further explore the association between matrix *MMP* gene polymorphisms and BC susceptibility in the Caucasian women of Russia.

## 2. Results

A comprehensive description of the phenotypic characteristics of the examined BC and cancer-free women is summarized in Table 1. The age range of the study participants was 28–84 years, with a predominance of postmenopausal women (2/3). There was no difference (*p* > 0.05) between the BC and cancer-free women regarding age, proportion of premenopausal/postmenopausal subjects, age at menarche, age at menopause, and smoker habits. At the same time, affected women had a higher body mass index (BMI) (*p* = 0.003) and a higher incidence of obesity when compared with the unaffected ones (*p* = 0.01). The majority of the BC patients were at clinical cancer stage T0–T2 (74%) and were predominantly estrogen receptor (ER) positive (66%), progesterone receptor (PR) positive (59%), and ductal carcinoma (94%) with a well differentiated/moderately differentiated (G1/G2) tumor histological grade (68%) (Table 1).

All ten investigated SNPs were in agreement with Hardy–Weinberg equilibrium (HWE) in both studied groups, BC and cancer-free woman, taking into account the Bonferroni correction for the number of examined loci (n = 10, *p*_bonf_ > 0.005 (0.05/10). According to the data of Supplementary Table S1, among the BC subjects, the observed values *p*_HWE_ were ≥0.015 and among the cancer-free group, the values were ≥0.086. Table 2 shows the association analysis results. Interestingly, the allele frequencies of the c.836 A>G (rs17576) and c. 1721 C>G (rs2250889) *MMP9* gene were significantly different between the BC and cancer-free subjects. Women carrying the G alleles of these loci, providing disorder protection, had an 18% (OR 0.82 95%CI 0.68–0.99 *p*  =  0.035 *p*_perm_ = 0.035) and 29% (OR 0.71 95%CI 0.52–0.97 *p*  =  0.033 *p*_perm_ = 0.034) lower BC risk than women with A (rs17576) and C (rs2250889) alleles, respectively. It should be noted that in the additive and dominant models, after adjustment for confounding factors, locus c. 1721 C>G (rs2250889) *MMP9* was associated with BC and reduced risk of developing BC (OR 0.69 95%CI 0.51–0.95 *p*  =  0.024 *p*_perm_ = 0.025 and OR 0.67 95%CI 0.47–0.95 *p*  =  0.026 *p*_perm_ = 0.026, respectively) (Table 2).

Next, we examined the associations between the *MMP9* gene haplotypes and BC. The detailed findings of the association analysis for various haplotypic constructions are presented in Table 3. We found eleven different haplotypes of the *MMP9* gene with six loci linked with BC, among which the vast majority of haplotypes determined an increased disorder risk (nine haplotypes) while only two haplotypes were associated with a low disease risk. Of note, five various haplotypic constructions (four-, five-, and six-locus) included different combinations of the allelic variants: rs3918242 (C), rs3918249 (T), rs17576 (A), rs3787268 (A), rs2250889 (C), and rs17577 (G) showed the most pronounced BC risk effect and increased the likelihood of development of the disease by more than three times (Table 3). Importantly, the direction of the effects of the c.836 A > G (rs17576) and c. 1721 C > G (rs2250889) loci in the composition of haplotypes fully corresponded to the nature of their independent association with the disease (minor G alleles of these loci were protective; major A (rs17576) and C (rs2250889) alleles were risky).

After demonstrating that individual SNPs and haplotypes in the *MMP9* gene may participate in BC susceptibility, we investigated the outcomes of interlocus interactions between all ten studied polymorphisms and five *MMP* genes. Using MB-MDR software (https://github.com/imbs-hl/mbmdR, accessed on 18 June 2022), the correlation between SNP interactions and BC was computed. According to the obtained simulation results, all 10 examined SNPs within 13 epistatic interaction models (5 (2-locus), 6 (3-locus), 2 (4-locus), and 1 (5-locus)) were associated with BC risk (Table 4). At the same time, it is important to note that, firstly, two polymorphisms: c.2003 G > A (rs17577) and c.-1562 C > T (rs3918242) *MMP9*, were included in the vast majority (>60%) of the significant models (9 and 8 models, respectively). Secondly, the two-locus combination: c.2003 G > A (rs17577) × c.-1562 C > T (rs3918242), formed the basis of 8 of the 13 considered genetic models, which indicates its key importance in the genetic determination of BC. The maximum value of the Wald indicator (WH = 48.71) was a five-locus model: c. 1721 G > C (rs2250889) *MMP9* × c.-1607 2G > 1G (rs1799750) *MMP1* × c.139-369 T > C (rs3918249) *MMP9* × c.259 T > C (rs1940475) *MMP8* × c.-1306 C > T (rs243865) *MMP2*.

The carriage of several specific genotype combinations of the *MMP9* gene had the most pronounced BC risk effect (beta = 1.54–1.61 and *p* = 0.0001–0.0004): GA(rs17577) × CC(rs2250889) × CC(rs3918242), GG(rs17577) × CC(rs2250889) × CT(rs3918242), GA(rs17577) × CC(rs3918242), GG(rs17577) × CT(rs3918242) (Supplementary Table S2). On the contrary, the combination of genotypes 1G1G (rs1799750) *MMP1* × TT (rs679620) *MMP3* showed a considerable protective effect on the BC relation (beta = −0.79 and *p* = 0.0004) (Supplementary Table S2).

**Figure 1:**
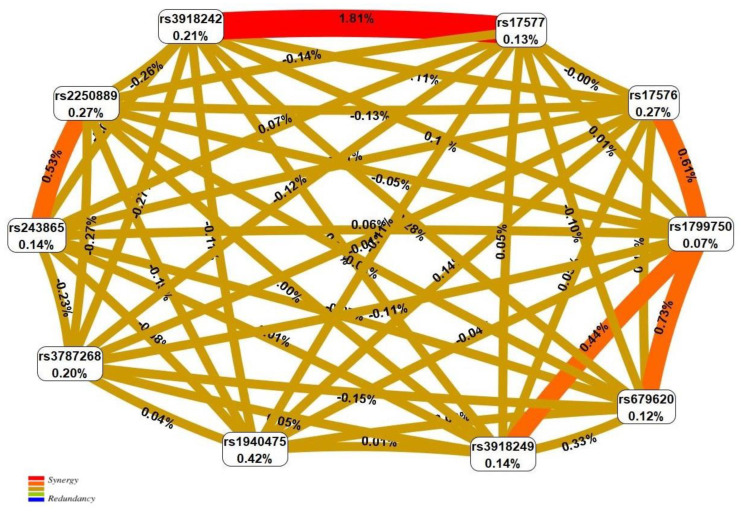
Entropy graph of the SNP × SNP interactions with breast cancer based on the MDR analysis. Positive entropy values indicate synergistic interactions while negative values indicate redundancy. The red and orange colors denote strong and moderate synergism, respectively; the brown color denotes an independent effect.

To visualize the established BC-involved interlocus interactions of *MMP* genes, a network (entropy graph) was constructed by multifactor dimensionality reduction (MDR) analysis (http://www.multifactordimensionalityreduction.org/, accessed on 18 June 2022). According the data shown in Figure 1, the largest contribution to BC susceptibility is by the synergistic two-locus interaction: c.2003 G > A (rs17577) × c.-1562 C > T (rs3918242) (which defines 1.81% of the BC entropy; in Figure 1, it is represented in red color), whose contribution to the BC predisposition was 4–5 times higher than any of the main effects of the individual loci (each of the studied loci determined no more than 0.27–0.42% of the entropy) (Figure 1).

### 2.1. Predicted Functional Outputs for BC-Associated SNPs

#### 2.1.1. Non-Synonymous (nsSNP) and Regulatory (regSNP) Impact

In total, 6 SNPs among 134 analyzed polymorphisms (10 BC-involved loci and 124 SNPs strongly liked with them) were nsSNPs such as c.836 A > G (rs17576) *MMP9* (p.Q279R), c. 1721 C > G (rs2250889) *MMP9* (p.574P), c.2003 G > A (rs17577) *MMP9* (p.R668Q), c.133 C > T (rs679620) *MMP3* (p.K45E), c.259 T > C (rs1940475) *MMP8* (p.K87E), and c.95 G > A (rs3765620) *MMP8* (p.L19V) (linked with rs1940475, r^2^ = 0.85 and D’ = 1.00) (Supplementary Table S3). All these nsSNPs are marked in the SIFT (https://sift.bii.a-star.edu.sg/, accessed on 18 June 2022) and PolyPhen-2 (http://genetics.bwh.harvard.edu/pph2/index.shtml, accessed on 18 June 2022) public databases as tolerated and benign, respectively (Supplementary Table S3).

In accordance with the HaploReg public epigenetic data (https://pubs.broadinstitute.org/mammals/haploreg/haploreg.php, accessed on 18 June 2022), we predicted a meaningful function of 10 BC-related polymorphic loci of *MMP* genes and the vast majority of strongly linked polymorphisms (121 loci out of 124 SNPs, 97.58%) in relation to 10 genes located next to them such as *MMP9*, *SLC12A5*, *MMP8*, *RP11-465L10.7*, *MMP3*, *RP11-465L10.10*, *MMP2*, *LOC100288077*, *MMP1*, and *ZNF335* (Supplementary Table S4). Interestingly, nine out of the 10 BC-associated loci (with the exception of rs17577) w located in the region of 42 regulatory motifs and determine their sensitivity to 39 TFs (transcription factors) (Supplementary Table S5). SNP c.-1607 2G > 1G (rs1799750) *MMP1* is situated in the region of the largest number of DNA motifs (n = 21). In general, 105 loci out of 124 strongly coupled polymorphisms (84.68%) define the DNA motifs’ sensitivity to plenty of TFs (Supplementary Table S4). A considerable quantity of loci among the studied 134 SNPs are located in functionally active regions of the genome such as hypersensibility to DNase (76 SNPs, 56.71%), enhancers (72 SNPs, 53.74%), promoters (49 SNPs, 36.56%), binding/interaction with regulatory proteins (21 SNPs, 15.67%), etc. (Supplementary Table S4).

At the same time, it is important to emphasize that almost all polymorphic loci associated with BC (with the exception of rs3918242) exhibit significant epigenetic effects in cultures of target organ cells in BC: mammary gland (primary epithelial and myoepithelial breast cells). So, in particular, c.836 A > G (rs17576) and c.2003 G > A (rs17577) *MMP9* are located in the promoter and hypersensibility to DNase regions, respectively, of HMEC mammary epithelial primary cells (Epigenome ID: E119, Mnemonic: BRST.HMEC); SNPs c.-1607 2G > 1G (rs1799750) *MMP1*, c.259 T > C (rs1940475) *MMP8*, c.836 A > G (rs17576) *MMP9*, c. 1721 C > G (rs2250889) *MMP9* are situated in enhancer sites of this mammary cell culture. The polymorphic loci c.836 A > G (rs17576), c. 1721 C > G (rs2250889), c. 1331-163 G > A (rs3787268), and c.2003 G > A (rs17577) of the *MMP9* gene are positioned in the enhancer points of the breast variant human mammary epithelial cells (vHMEC) (Epigenome ID: E028, Mnemonic: BRST.HMEC.35). SNPs c.-1306 C > T (rs243865) *MMP2*, c.133 C > T (rs679620) *MMP3*, c.139-369 T > C (rs3918249) *MMP9*, c. 1331-163 G > A (rs3787268) *MMP9*, c.2003 G > A (rs17577) *MMP9*, c.836 A > G (rs17576) *MMP9*, c. 1721 C > G (rs2250889) *MMP9*, and c.836 A > G (rs17576) *MMP9* are disposed in the enhancer and promoter regions, respectively, of breast myoepithelial primary cells (Epigenome ID: E027, Mnemonic: BRST.MYO). A significant number of strongly linked loci also exhibit functional effects in primary epithelial and myoepithelial breast cells (Supplementary Table S4).

#### 2.1.2. Expression (eSNP) and Splicing (sSNP) Impact

In accordance with the Blood eQTL Browser (http://genenetwork.nl/bloodeqtlbrowser/, accessed on 18 June 2022), two eSNPs, c. 1331-163 G > A (rs3787268) *MMP9* and c.-1306 C > T (rs243865) *MMP2*, are blood expression quantitative trait loci (eQTL), which are related and displayed an association (pFDR = 0) with the transcription level of three genes, such as *AL162458.10-3*, *MMP9* (Z score = −11.51), and *AYTL1* (Z score = −14.37). In addition, 18 loci are highly linked with 4 BC-associated polymorphisms, including the expression regulators of four genes (*DNTTIP1*, *AL162458.10-3*, *MMP9*, *AYTL1*) in peripheral blood (Supplementary Table S6).

Furthermore, messenger ribonucleic acid (mRNA) expression and splicing information obtained from the GTEx portal database (https://gtexportal.org/home/, accessed on 18 June 2022) demonstrated, firstly, that 10 BC risk loci and 115 SNPs among 124 loci strongly linked to them (92.74%) are identified by eQTL for 23 genes, such as *RP11-212I21.2*, *RP11-465L10.10*, *RP11-817J15.3*, *RP3-337O18.9*, *RPL13P2*, *CD40*, *DNTTIP1*, *MMP1*, *MMP10*, *PCIF1*, *NEURL2*, *MP27*, *MMP9*, *PLTP*, *SLC12A5*, *SNX21*, *SPATA25*, *SYS1*, *WFDC10B*, *WFDC3*, *WTAPP1*, *ZNF335*, and *ZSWIM1* (Supplementary Tables S7 and S8). Secondly, 8 BC-involved polymorphisms and 97 coupled loci are sSNPs for 6 genes, such as *SNX21*, *WTAPP1*, *ACOT8*, *CD40*, *PLTP*, and *SLC12A5* (Supplementary Tables S9 and S10).

It is extremely interesting that 3 BC-associated SNPs, c.-1607 2G > 1G (rs1799750) *MMP1*, c.2003 G > A (rs17577) *MMP9*, and c.-1562 C > T (rs3918242) *MMP9*, and 12 loci linked to the last 2 SNPs are eSNPs (*MMP1*, *SNX21*, *SLC12A5*) in BC target organ: the mammary gland (Supplementary Tables S7 and S8). Additionally, interestingly, polymorphism c. 1331-163 G > A (rs3787268) *MMP9* and 12 coupled SNPs are sSNPs (*PLTP*) in breast tissue (Supplementary Tables S9 and S10).

### 2.2. Identification of Biological Pathways for BC Putative Target Genes

As a summary of our research, we deciphered the biological mechanisms (molecular functions; biological processes; cellular components; reactomic pathways; protein classes) of the BC putative target genes obtained based on the considered causal SNP (with linked loci) functional variants (nsSNP, regSNPs, eSNP, sSNP). So, in particular, c.836 A > G (rs17576) and c.2003 G > A (rs17577) *MMP9* are located in the promoter and hypersensibility to DNase regions, respectively, of HMEC mammary epithelial primary cells (Epigenome ID: E119, Mnemonic: BRST.HMEC); SNPs c.-1607 2G > 1G (rs1799750) *MMP1*, c.259 T > C (rs1940475) *MMP8*, c.836 A > G (rs17576) *MMP9*, and c. 1721 C > G (rs2250889) *MMP9* are situated in the enhancer sites of this mammary cell culture. At the beginning, we examined the biological pathways of 32 genes functionally related to BC causal loci and SNPs linked to them in various organs and tissues of the organism (Supplementary Tables S3, S4 and S7–S10). Then, we evaluated the significant enrichment of the pathways for breast-specific genes only (*n* = 12; *LOC100288077*, *RP11-465L10.10*, *RP11-465L10*, *MMP1*, *MMP2*, *MMP3*, *MMP8*, *MMP9*, *ZNF335*, *SLC12A5*, *PLTP*, *SNX21*), i.e., genes that are functionally appreciable (missense mutations; eQTL; sQTL; regulatory) in the mammary gland (Supplementary Tables S3, S4 and S7–S10).

**Figure 2:**
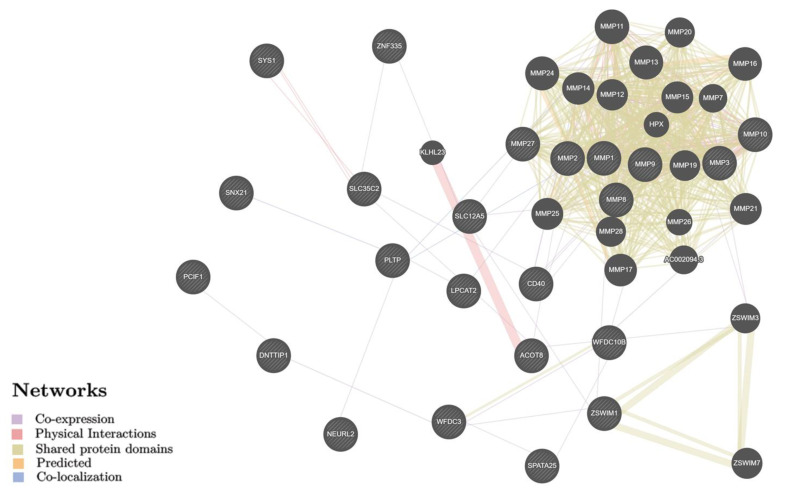
The interaction networks of the candidate genes for breast cancer in various tissues/organs inferred using GeneMANIA (http://genemania.org, accessed on 18 June 2022). The candidate genes are cross-shaded.

Using the Gene Ontology biological resource (http://geneontology.org/, accessed on 18 June 2022), about 50 different biological pathways were identified (Supplementary Table S11), which are largely related to metalloendopeptidase activity and extracellular matrix organization (structure, disassembly, metabolic process, etc.). Several biological mechanisms demonstrated a Fold Enrichment indicator of more than 100 such as activation of matrix metalloproteinases, extracellular matrix disassembly, collagen metabolic/catabolic process, cellular response to UV-A, and plasminogen-activating cascade (Supplementary Table S11). The interaction network of BC candidate genes (Figure 2, obtained by the GeneMANIA (https://genemania.org/, accessed on 18 June 2022)) indicates a key role in the following interactions: cooperative expression of genes (40.67%), overall domains of proteins (30.90%), and physical (positional) communications (15.96%), for which the leading role was paired gene–gene mutual influence of *KLHL23–ACOT8*, *MMP3–MMP10*, *ZSWIM7–ZSWIM1*, *ZSWIM3–ZSWIM1*, and *ZSWIM3–ZSWIM7* (the weight indicators are equal 0.43–0.55) (Supplementary Table S12).

**Figure 3:**
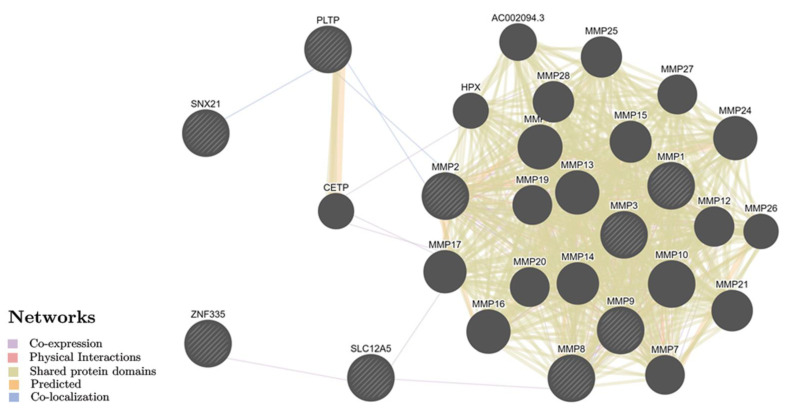
The interaction networks of the breast cancer candidate genes that are functionally significant for breast tissue inferred using GeneMANIA (http://genemania.org, accessed on 18 June 2022). The candidate genes are cross-shaded.

The biological mechanisms characteristic of the 12 breast-specific genes (we identified more than 60 of these pathways; Supplementary Table S13) are very like those identified for the above-mentioned 32 BC-involved genes (Supplementary Table S11) and are in a large part represented by metalloendopeptidase activity/extracellular matrix organization, etc. The breast-specific gene interactions (Figure 3) are mainly realized due to the overall domains of proteins (45.22%) and positional communications (38.04%) with the maximum weight index (0.52) of the *CETP–PLTP* genes paired interaction (Supplementary Table S14).

## 3. Discussion

In our report, we demonstrated the involvement of *MMP* gene polymorphisms in BC susceptibility in the Caucasian women of Russia. All examined ten SNPs of the *MMP* genes were BC-associated: two loci (c.836 A > G (rs17576) and c. 1721 C > G (rs2250889) *MMP9*) showed an independent disease protective effect, eleven haplotypes of six SNPs *MMP9* were mostly involved with BC risk, and thirteen SNP–SNP interaction simulated models are correlated with BC. Under in silico materials, BC susceptibility *MMP* polymorphic loci are located in functionally active regions of the breast genome, impact on epigenetic processes, and are regulators of gene expression/splicing.

Our results demonstrated a protective impact of the G allele (the C allele is risky) of SNP c. 1721 C > G (rs2250889) *MMP9* on BC formation, both independently (OR 0.67–0.71) and as part of eight haplotypes of the *MMP9* gene. The relationship of c. 1721 C > G (rs2250889) *MMP9* with BC was considered in seven previously published works: four experimental studies (Malaysian, Chinese, and Jordanian populations were studied) [25,27,29,30] and three meta-analyses [20,21,22]. The association of this polymorphism with disease was demonstrated in two of these studies (one experiment performed in a Malaysian population [29] and one meta-analysis [21]). It should be emphasized that the GG genotype (OR 10.84) has a risk value for BC in Malaysian women [29] while in Caucasian women in Russia, the G allele exhibits a protective effect in relation to the disease (our data). The above data make it possible to suggest the presence of interethnic peculiarities (Asian/European) in the association of c. 1721 C > G (rs2250889) *MMP9* with BC; however, this assumption needs to be confirmed in further molecular and genetic studies.

Another *MMP9* gene polymorphism independently associated with BC in Caucasian women of Russia, c.836 A > G (rs17576) (the G allele is protective, OR 0.82) also contributes to the risk of disorder within nine haplotypes of the *MMP9* gene (the A allele is risky). Data from the literature on the association between c.836 A > G (rs17576) *MMP9* and the disease are very contradictory. No associations of this locus with the disease were identified in two works [25,27] while opposite results (risk/protective value of polymorphism) were demonstrated in another three studies [29,31,32]. In three meta-analyses, significant associations of c.836 A > G (rs17576) *MMP9* with breast cancer were not established [20,21,22]. Our data on the protective effect of the G allele (rs17576) on breast cancer in Caucasian women of Russia are consistent with the results of Resler et al., who studied white women of the Seattle metropolitan area (USA) [31], and contrast with the data obtained in Malaysian [29] and Brazilian [32] populations (according to their data, the G allele is risky).

We obtained interesting data in silico regarding the pronounced cell/organ-specific functional effects of c.836 A > G (rs17576) and c. 1721 C > G (rs2250889) *MMP9* in the mammary gland, which is a BC target organ. These BC risk loci (together with coupled SNPs) are involved in amino acid modification of *MMP9* protein (p.Q279R and p.574P, respectively), epigenetic control gene activity due to enhancer- and promoter-driven transcriptions in primary epithelial and myoepithelial breast cells, and gene expression/splicing level regulation in BC-related organs (brain, adipose, adrenal gland, thyroid, blood, etc.). The above-stated SNPs’ functional actions may be the pathophysiological basis of these loci associations with BC.

The *MMP9* protein (gelatinase B), resulting from the transcription of the gene of the same name, is a well-known multifunctional collagenase [33,34]. *MMP9*, due to its ability to destroy type IV collagen and denatured collagens, resulting in the destruction of basement membranes, is essential for the biology of tumors, including BC [34,35]. Importantly, *MMP9* protein is constitutively expressed by breast cancer cell lines (MDA-MB-231) [33]. The tumorigenic effects of *MMP9* are realized in lots of ways, such as DNA degradation and accompanying molecular changes, epithelial to mesenchymal transition and cancer stem cell formation/support, matrix degradation, production and activation of matrix-associated and non-matrix cytokines, inflammatory tumor-related activity, cancer immunological monitoring regulation, change in the cyto-skeletal organization during tumorigenesis (tumor–stromal relationship, etc.), proliferation and invasion of cancer cells, effect on angiogenesis (apoptosis) factors, etc., which predetermines the fundamental role of this collagenase in BC progression and metastasis, relapse, and survival of disease-affected patients [34].

There is convincing evidence of an important prognostic value of the *MMP9* expression level in BC patients [36,37]. As a result of a meta-analysis (based on data on 2344 women with BC from 15 studies), Song et al. showed a direct link between overexpression of *MMP9* and both relapse-free and overall survival in BC patients [36]. The meta-analysis performed by Jiang et al. (the materials of 41 experimental studies, including 6517 BC patients, were used) not only shows the association of increased expression of *MMP9* with a shorter overall survival of BC-affected women but also the association of high expression of *MMP9* with other BC clinical (clinicopathological) signs such as histological grade, clinical stage, larger tumor sizes, and lymph node metastasis [37]. The literature suggest a link between an increased content of MMP9 protein and negative status of hormonal receptors and a higher tumor grade [35]. As a result of an experimental study of patients with primary early BC, higher expression of *MMP9* in tumor cells was found in comparison with tumor-associated stroma and its correlation with a tumor cell proliferation index [38]. A higher level of *MMP9* protein in the tumor than in normal tissue and its direct connection with increased tumor size were also reported in the work of Przybylowska et al. [12]. The expression of *MMP9* protein concerns important regulators/markers of the cell cycle/ECM remodeling such as cell division control protein 42, epidermal growth factor and cell surface adhesion receptors, cytokeratin 17, and Ki67 proliferation biomarker [35]. The abovementioned data indicate the considerable putative clinical and pathophysiological significance of *MMP9* in BC and, consequently, functionally meaningful polymorphisms of this gene may become important prognostic markers of this disease in the future.

## 4. Materials and Methods

### 4.1. Study Subjects

We enrolled 358 affected (BC) and 746 unaffected (cancer-free) women at the Belgorod Regional Oncological Dispensary and Belgorod Regional Clinical Hospital of St. Ioasaph from March 2010 to December 2016. All participants were Caucasian women who were born and living in central Russia [39,40]. BC diagnosis was histopathologically confirmed by certified pathologists. The control group were BC free (confirmed by mammography or breast ultrasound) without malignant tumor history and heavy disorder-related to the vital organs. We obtained ethical approval from the Human Investigation (Ethics) Committee of Belgorod State National Research University.

### 4.2. SNP Selection and Genotyping

We selected the phenol-chloroform method to obtain quality DNA extract from the peripheral venous blood samples (≈4–5 mL) [41]. DNA extraction was carried out according previously described procedure [42]. A Nanodrop 2000 was used to detect the DNA concentration and purity [43].

*MMP* gene SNPs associated with BC reported in more than 50 previous association studies [12,16,17,18,19,20,21,22,23,24,25,26,27,28] (Supplementary Table S15) and with potential regulatory functions (Supplementary Table S16) [44,45,46,47,48,49,50] were selected for our genetic–epidemiological study. As a result, 10 SNPs of the *MMP* genes were investigated in this study, including 6 loci of chromosome 20 (c.139-369 T > C (rs3918249) *MMP9*, c. 1331-163 G > A (rs3787268) *MMP9*, c.2003 G > A (rs17577) *MMP9*, c.-1562 C > T (rs3918242) *MMP9*, c.836 A > G (rs17576) *MMP9*, c. 1721 C > G (rs2250889) *MMP9*), 3 SNP of chromosome 11 (c.259 T > C (rs1940475) *MMP8*, c.133 C > T (rs679620) *MMP3*, c.-1607 2G > 1G (rs1799750) *MMP1*), and 1 locus of chromosome 16 (c.-1306 C > T (rs243865) *MMP2*).

The standard real-time PCR genotyping system (CFX96, Bio-Rad) was used to determine the genotypes of all participants (BC and cancer-free) [51]. Quality control was used in the genotyping procedure [52]. As a result, the percentage of genotyping errors did not exceed 0.5%.

### 4.3. Statistical Analysis

The SNP genotype frequencies in the BC and cancer-free subjects were analyzed separately to find out the compliance with Hardy–Weinberg equilibrium [53]. Differences in the distribution of polymorphic variant frequencies between the BC and cancer-free groups were evaluated based on four common genetic models (recessive, additive, dominant, and allelic) by logistic regression [54] with age and BMI adjustment. Association links of genetic markers with BC were appreciated based on the indicators’ OR (odds ratio) and 95% CIs (confidence intervals for OR) [55]. The individual SNPs and their haplotype association analyses were executed using gPLINK software [56]. The MB-MDR and MDR methods and the corresponding software packages were utilized to evaluate the association of SNP interactions with BC [57] (http://sourceforge.net/projects/mdr, accessed on 18 June 2022). Among the many obtained simulation epistatic models for subsequent permutation procedures, we selected models corresponding to the level of statistical significance chosen by us according to the Bonferroni amendment (taking into account the 10 examined loci combinations), such as *p*_interact_ < 1.11 × 10^−3^ (<0.05/45) (2–locus models), *p*_interact_ < 4.17 × 10^−4^ (<0.05/120) (3–locus), *p*_interact_ < 2.38 ×10^−4^ (<0.05/210) (4–locus), and *p*_interact_ < 1.98 × 10^−4^ (<0.05/252) (5–locus). The association analysis results were corrected for multiple testing by a permutation test [58,59]. As a result, *p*_perm_ value ≤ 0.05 for individual SNPs (adaptive permutations were carried out [54], their haplotypes, and SNP*SNP interactions (1000 permutations were done [60]) was recognized as the cutoff parameter for statistical significance.

### 4.4. SNPs and Genes Predict Functions

We examined in silico the prediction functions of BC-involved SNP *MMP* genes and their strongly linked variants (r^2^ equal to and more than 0.80) [61] based on the value widely used in human genetics bioinformatics resources such as GTExproject [62], PolyPhen-2 [63], HaploReg [64], Blood eQTL browser [65], SIFT [66], GeneMANIA [67], and Gene Ontology [68].

## 5. Conclusions

*MMP* gene polymorphisms located in functionally active genome regions in the mammary gland are related to BC susceptibility in the Caucasian women of Russia.

## Figures and Tables

**Table 1:** Phenotypic characteristics of the study participants.

Parameters	BC Patients, % (n)	Controls,% (n)	*p*
*N*	358	746	-
Age, years (min–max)	55.74 ± 12.79 (28–84)	55.29 ± 12.27 (30–82)	0.54
<50 years	33.80 (121)	35.12 (262)	0.72
≥50 years	66.20 (237)	64.88 (484)	
BMI, kg/m^2^	30.27 ± 6.13	28.19 ± 5.73	0.003
Obesity (BMI ≥ 30) (yes)	33.24 (119)	25.47 (190)	0.01
Age at menarche, years	12.42 ± 1.12	12.64 ± 1.14	0.58
Age at menopause, years	48.27 ± 5.02	47.97 ± 4.91	0.48
Mensuration status			
Premenopause	31.84 (114)	34.05 (254)	0.51
Postmenopause	68.16 (244)	65.95 (492)	
Smoker (yes)	22.07 (79)	18.77 (140)	0.22
Clinicopathological parameters of BC patients			
Stage of the cancer	T_0_–T_2_—74%, T_3_–T_4_—26%		
Lymph node involvement (N)	negative—47%, positive—53%		
Estrogen receptor (ER)	negative—34%, positive—66%		
Progesterone receptor (PR)	negative—41%, positive—59%		
Human epidermal growth factor receptor 2 (HER2)	negative—64%, positive—36%		
Triple negative	22%		
Tumor histological type	ductal—94%, lobular—6%		
Tumor histological grade (G)	G1/G2—68%, G3—32%		
Progression	absent—66%, present—34%		
Metastasis	absent—78%, present—22%		
Death	absent—81%, present—19%		

Note: G1—well differentiated; G2—moderately differentiated; G3—poorly differentiated.

**Table 2:** Associations of the studied gene polymorphisms with breast cancer.

SNP	Gene	Minor Allele	n	Allelic Model				Additive Model				Dominant Model				Recessive Model			
				OR	95%CI		*p*	OR	95%CI		*p*	OR	95%CI		*p*	OR	95%CI		*p*
					L95	U95			L95	U95			L95	U95			L95	U95	
rs1799750	*MMP-1*	2G	1077	1.02	0.85	1.22	0.831	1.05	0.87	1.25	0.626	1.16	0.87	1.54	0.325	0.96	0.70	1.32	0.811
rs243865	*MMP-2*	T	1082	0.93	0.75	1.15	0.491	0.95	0.77	1.18	0.651	1.01	0.78	1.32	0.922	0.68	0.38	1.20	0.185
rs679620	*MMP-3*	T	1095	0.90	0.76	1.08	0.267	0.87	0.72	1.05	0.135	0.88	0.65	1.18	0.381	0.78	0.57	1.06	0.117
rs1940475	*MMP-8*	T	1096	0.97	0.81	1.16	0.742	1.00	0.84	1.20	0.989	1.22	0.91	1.64	0.189	0.81	0.60	1.10	0.185
rs3918242	*MMP-9*	T	1089	1.02	0.80	1.30	0.869	1.04	0.82	1.33	0.744	0.96	0.73	1.28	0.802	1.82	0.90	3.70	0.097
rs3918249	*MMP-9*	C	1083	0.88	0.73	1.06	0.180	0.90	0.75	1.09	0.294	0.83	0.63	1.08	0.161	0.97	0.68	1.40	0.890
rs17576	*MMP-9*	G	1095	**0.82**	**0.68**	**0.99**	**0.035**	0.84	0.70	1.01	0.068	0.78	0.60	1.01	0.063	0.82	0.57	1.19	0.302
rs3787268	*MMP-9*	A	1089	1.11	0.89	1.37	0.352	1.11	0.89	1.38	0.350	1.05	0.80	1.36	0.746	1.68	0.95	2.96	0.074
rs2250889	*MMP-9*	G	1090	**0.71**	**0.52**	**0.97**	**0.033**	**0.69**	**0.51**	**0.95**	**0.024**	**0.67**	**0.47**	**0.95**	**0.026**	0.54	0.18	1.67	0.286
rs17577	*MMP-9*	A	1079	0.97	0.76	1.23	0.798	0.98	0.77	1.25	0.850	0.92	0.69	1.22	0.556	1.41	0.69	2.85	0.343

Note: OR: odds ratio; 95% CI: 95% confidence interval; *p* values < 0.05 are shown in bold; all results were obtained after adjustment for covariates.

**Table 3:** Significance associations of the *MMP-9* gene haplotypes with breast cancer risk.

SNPs	Haplotype	Frequency		OR	*p*_raw value_	*p*_perm_
		Cases	Controls			
risk effect						
rs17576-rs3787268	AA	0.0377	0.0186	2.46	0.004	0.020
rs17576-rs3787268-rs2250889	AAC	0.0358	0.0179	2.53	0.001	0.012
rs3918249-rs17576-rs3787268	TAA	0.0254	0.0110	2.89	0.004	0.034
rs17576-rs3787268-rs2250889-rs17577	AACG	0.0375	0.0171	2.68	0.003	0.020
rs3918249-rs17576-rs3787268-rs2250889	TAAC	0.0246	0.0102	3.21	0.004	0.031
rs3918242-rs3918249-rs17576-rs3787268	CTAA	0.0252	0.0101	3.07	0.005	0.032
rs3918249-rs17576-rs3787268-rs2250889-rs17577	TAACG	0.0263	0.0095	3.63	0.003	0.016
rs3918242-rs3918249-rs17576-rs3787268-rs2250889	CTAAC	0.0247	0.0092	3.55	0.002	0.016
rs3918242-rs3918249-rs17576-rs3787268-rs2250889-rs17577	CTAACG	0.0247	0.0090	3.26	0.003	0.046
protective effect						
rs3787268-rs2250889	GG	0.0693	0.1011	0.63	0.013	0.032
rs3787268-rs2250889-rs17577	GGG	0.0710	0.0994	0.66	0.017	0.050

Note: OR: odds ratio; *p*: significance level; all results were obtained after adjustment for covariates.

**Table 4:** SNP × SNP interactions significantly associated with breast cancer.

N	SNP × SNP Interaction Models	NH	*beta* H	WH	NL	*beta* L	WL	*p*_perm_
Two-order interaction models (*p* < 1.02 × 10^−3^)								
1	rs17577 *MMP9* × rs3918242 *MMP9*	2	1.589	29.73	1	−0.434	7.15	<0.001
2	rs1799750 *MMP1* × rs17576 *MMP9*	2	0.502	13.58	1	−0.608	8.24	0.004
3	rs17577 *MMP*9 × rs17576 *MMP9*	2	1.256	12.47	0	-	-	0.005
4	rs1799750 *MMP1* × rs679620 *MMP3*	2	0.413	7.38	1	−0.788	12.65	0.011
5	rs2250889 *MMP9* × rs243865 *MMP2*	0	-	-	2	−0.674	10.79	0.011
Three-order interaction models (*p* < 2.15 × 10^−7^)								
1	rs17577 *MMP9* × rs3918242 *MMP9 ×* rs1940475 *MMP8*	6	1.620	32.65	1	−0.536	3.11	<0.001
2	rs17577 *MMP9* × rs3918242 *MMP9* × rs243865 *MMP2*	4	1.654	31.11	1	−0.702	7.13	<0.001
3	rs17577 *MMP9* × rs3787268 *MMP9* × rs3918242 *MMP9*	4	1.545	27.67	2	−0.452	7.67	<0.001
4	rs17577 *MMP9* × rs1799750 *MMP1* × rs3918242 *MMP9*	5	1.640	27.52	2	−0.918	14.91	<0.001
5	rs17577 *MMP9* × rs3918242 *MMP9* × rs679620 *MMP3*	5	1.538	27.38	1	−1.189	10.40	<0.001
6	rs17577 *MMP9 ×* rs2250889 *MMP9* × rs3918242 *MMP9*	4	1.620	26.90	1	−0.352	4.22	<0.001
Four-order interaction models (*p* < 9.93 × 10^−8^)								
1	rs17577 *MMP9* × rs3787268 *MMP9* × rs3918242 *MMP9* × rs243865 *MMP2*	7	1.341	28.39	2	−0.702	7.13	<0.001
2	rs1799750 *MMP1* × rs3918249 *MMP9* × rs194047 *MMP8* × rs243865 *MMP2*	6	0.993	30.90	1	−1.086	3.94	<0.001
Five-order interaction models (*p* = 2.96 × 10^−12^)								
1	rs2250889 *MMP9* × rs1799750 *MMP1* × rs3918249 *MMP9* × rs1940475 *MMP8* × rs243865 *MMP2*	9	1.308	48.71	0	-	-	<0.001

Note: NH: number of significant high risk genotypes in the interaction; beta H: regression coefficient for high-risk exposition in the step 2 analysis; WH: Wald statistic for the high-risk category; NL: number of significant low-risk genotypes in the interaction; beta L: regression coefficient for low-risk exposition in the step 2 analysis; WL: Wald statistic for the low-risk category; *p*_perm_: permutation *p*-value for the interaction model (1.000 permutations); the results were obtained using the MB-MDR method with adjustment for covariates.

## Data Availability

The data generated in the present study are available from the corresponding author upon reasonable request.

## Supplementary Materials

The following supporting information can be downloaded at: https://www.mdpi.com/article/10.3390/ijms232012638/s1. References [69,70,71,72,73,74,75,76,77,78,79,80,81,82,83,84,85,86,87,88,89,90,91,92,93,94,95,96,97,98,99,100,101,102,103] are cited in the supplementary materials.
