# Supplementary material for: Matrix Metalloproteinase Gene Polymorphisms Are Associated with Breast Cancer in the Caucasian Women of Russia
Source: Int J Mol Sci. 2022 Oct 20;23(20):12638. doi: 10.3390/ijms232012638 (PMC9604098; doi:10.3390/ijms232012638)
Supplement: Supplementary file 1 [file ijms-23-12638-s001.zip › Suppl table s11.pdf]

Supplementary Table S11 Gene set enrichment analysis of biological pat

(Gene Ontology Portal tools, http://geneontology.org).

Database	Gene set	Original # genes in gene set
GO biological process complete	cellular response to UV-A	11
	response to UV-A	14
	response to UV	150
	cellular response to UV	91
	cellular response to light stimulus	116
	cellular response to radiation	181
	cellular response to abiotic stimulus	320
	cellular response to environmental stimul	320
	collagen catabolic process	38
	collagen metabolic process	57
	extracellular matrix disassembly	47
	extracellular matrix organization	294
	extracellular structure organization	295
	external encapsulating structure organizat	297
	cellular component disassembly	300
	regulation of neuroinflammatory response	35
	endodermal cell differentiation	41
	endoderm formation	51
	response to amyloid-beta	49
GO molecular function complete	metalloendopeptidase activity	114
	endopeptidase activity	457
	peptidase activity	643
	metallopeptidase activity	190
	serine-type endopeptidase activity	180
	serine-type peptidase activity	198
	serine hydrolase activity	202
	zinc ion binding	837
	transition metal ion binding	1113
GO cellular component complete	extracellular matrix	572
	external encapsulating structure	573

PANTHER Pathways	Plasminogen activating cascade	17
	Alzheimer disease-presenilin pathway	131
PANTHER GO-Slim Molecular Function	metalloendopeptidase activity	84
	endopeptidase activity	324
	peptidase activity	433
	hydrolase activity	1739
	metallopeptidase activity	116
PANTHER GO-Slim Biological Process	extracellular matrix organization	74
	external encapsulating structure organizat	74
	extracellular structure organization	75
	catabolic process	893
PANTHER Protein Class	metalloprotease	155
	protease	595
	protein modifying enzyme	1622
Reactome pathways	Activation of Matrix Metalloproteinases	33
	Degradation of the extracellular matrix	140
	Collagen degradation	64
	Extra-nuclear estrogen signaling	73
	Interleukin-4 and Interleukin-13 signaling	111

hways with 32 BC-associated genes (BC-associated polymorphisms functionality in var

# genes in gene set analyzed by Gene Ontology	Fold Enrichment	raw P-value	FDR
4	> 100	0.00000000159	0.00000625
4	> 100	0.00000000357	0.0000112
4	23.87	0.0000238	0.0266
4	39.35	0.00000351	0.00549
4	30.87	0.00000888	0.0127
4	19.78	0.0000487	0.0401
5	13.99	0.0000252	0.0263
5	13.99	0.0000252	0.0247
7	> 100	0.0000000000000346	0.000000000542
7	> 100	0.000000000000468	0.00000000366
6	> 100	0.0000000000211	0.00000011
7	21.31	0.0000000265	0.0000692
7	21.24	0.0000000271	0.0000607
7	21.01	0.0000000284	0.0000556
6	17.09	0.000000832	0.00145
3	76.73	0.00000998	0.013
3	65.5	0.0000156	0.0188
3	52.66	0.000029	0.0253
3	54.81	0.0000259	0.0239
7	54.97	0.0000000000457	0.000000224
7	13.71	0.000000502	0.000411
7	27638	0.00000474	0.00291
7	32.98	0.00000000141	0.00000346
6	29.84	0.0000000443	0.0000724
6	27.13	0.0000000766	0.000094
6	26.59	0.0000000859	0.0000844
8	8.56	0.00000219	0.00153
8	6.43	0.0000176	0.00958
7	10.95	0.00000221	0.0045
7	11.94	0.00000223	0.00228

4	> 100	0.00000000696	0.00000111
5	34.17	0.000000355	0.0000284
7	74.6	0.00000000000598	0.00000000327
7	19.34	0.0000000508	0.00000925
7	14.47	0.000000351	0.000048
8	4.12	0.000403	0.044
7	54.02	0.0000000000514	0.000000014
7	84.68	0.00000000000259	0.00000000574
7	84.68	0.00000000000259	0.00000000287
7	83.55	0.00000000000283	0.00000000209
8	8.02	0.00000353	0.00196
7	40.43	0.000000000359	0.0000000703
7	10.53	0.00000286	0.00028
8	4.42	0.00025	0.0164
6	> 100	0.00000000000302	0.00000000754
6	38.36	0.0000000104	0.00000865
6	83.92	0.000000000119	0.000000148
3	36.79	0.0000809	0.0336
4	32.26	0.0000075	0.00374
