# Supplementary material for: Matrix Metalloproteinase Gene Polymorphisms Are Associated with Breast Cancer in the Caucasian Women of Russia
Source: Int J Mol Sci. 2022 Oct 20;23(20):12638. doi: 10.3390/ijms232012638 (PMC9604098; doi:10.3390/ijms232012638)
Supplement: Supplementary file 1 [file ijms-23-12638-s001.zip › Suppl table s12.pdf]

Supplementary Table S12 Results of the gene-gene interaction analysis of the BC-assoc

Gene 1	Gene 2	Weight	Network group	Network
WFDC3	*DNTTIP1*	0.021363324	Co-expression	Arijs-Rutgeerts-2009
MMP8	*MMP10*	0.025001792	Co-expression	Arijs-Rutgeerts-2009
MMP1	*MMP10*	0.016835684	Co-expression	Arijs-Rutgeerts-2009
MMP1	*MMP8*	0.01994568	Co-expression	Arijs-Rutgeerts-2009
MMP3	*MMP10*	0.014015151	Co-expression	Arijs-Rutgeerts-2009
MMP3	*MMP8*	0.014503988	Co-expression	Arijs-Rutgeerts-2009
MMP3	*MMP1*	0.012034981	Co-expression	Arijs-Rutgeerts-2009
SLC12A5	*ZSWIM1*	0.0035285335	Co-expression	Arijs-Rutgeerts-2009
SLC12A5	*ZNF335*	0.003524543	Co-expression	Arijs-Rutgeerts-2009
MMP9	*MMP10*	0.016440187	Co-expression	Arijs-Rutgeerts-2009
MMP9	*MMP8*	0.018592577	Co-expression	Arijs-Rutgeerts-2009
MMP9	*MMP1*	0.011928413	Co-expression	Arijs-Rutgeerts-2009
MMP9	*MMP3*	0.009092866	Co-expression	Arijs-Rutgeerts-2009
MMP11	*SLC12A5*	0.0041121915	Co-expression	Arijs-Rutgeerts-2009
MMP13	*MMP8*	0.020443471	Co-expression	Arijs-Rutgeerts-2009
MMP21	*WFDC10B*	0.018210186	Co-expression	Arijs-Rutgeerts-2009
MMP12	*MMP10*	0.018506225	Co-expression	Arijs-Rutgeerts-2009
MMP12	*MMP8*	0.016926065	Co-expression	Arijs-Rutgeerts-2009
MMP12	*MMP1*	0.011808239	Co-expression	Arijs-Rutgeerts-2009
MMP12	*MMP3*	0.009681639	Co-expression	Arijs-Rutgeerts-2009
MMP12	*MMP9*	0.012654959	Co-expression	Arijs-Rutgeerts-2009
MMP7	*MMP10*	0.01624499	Co-expression	Arijs-Rutgeerts-2009
MMP26	*MMP27*	0.003192505	Co-expression	Arijs-Rutgeerts-2009
WFDC3	*ZSWIM1*	0.009348901	Co-expression	Innocenti-Brown-2011
MMP1	*MMP10*	0.025890643	Co-expression	Innocenti-Brown-2011
CD40	*SLC35C2*	0.01798979	Co-expression	Innocenti-Brown-2011
MMP3	*MMP10*	0.03973388	Co-expression	Innocenti-Brown-2011
MMP3	*MMP1*	0.021670682	Co-expression	Innocenti-Brown-2011
SLC12A5	*MMP8*	0.01042752	Co-expression	Innocenti-Brown-2011
PLTP	*AYTL1*	0.015930394	Co-expression	Innocenti-Brown-2011
MMP13	*MMP24*	0.019798096	Co-expression	Innocenti-Brown-2011
MMP17	*WFDC10B*	0.006819241	Co-expression	Innocenti-Brown-2011
MMP25	*MMP9*	0.032020573	Co-expression	Innocenti-Brown-2011
WFDC10B	*SPATA25*	0.008939263	Co-expression	Mallon-McKay-2013
WFDC3	*SPATA25*	0.008462458	Co-expression	Mallon-McKay-2013
WFDC3	*WFDC10B*	0.019885043	Co-expression	Mallon-McKay-2013
ZNF335	*SLC35C2*	0.005005366	Co-expression	Mallon-McKay-2013
MMP1	*MMP10*	0.023359546	Co-expression	Mallon-McKay-2013
PLTP	*NEURL2*	0.017989358	Co-expression	Mallon-McKay-2013
ZSWIM3	*ACOT8*	0.008891669	Co-expression	Mallon-McKay-2013
PCIF1	*DNTTIP1*	0.014498062	Co-expression	Ramaswamy-Golub-2001
MMP1	*MMP10*	0.029388728	Co-expression	Ramaswamy-Golub-2001
MMP3	*MMP1*	0.031768974	Co-expression	Ramaswamy-Golub-2001

MMP13	*MMP10*	0.0159287	Co-expression	Ramaswamy-Golub-2001
MMP12	*MMP1*	0.053733964	Co-expression	Ramaswamy-Golub-2001
MMP12	*MMP3*	0.028386803	Co-expression	Ramaswamy-Golub-2001
MMP7	*MMP11*	0.014169933	Co-expression	Ramaswamy-Golub-2001
MMP7	*MMP12*	0.023871709	Co-expression	Ramaswamy-Golub-2001
ACOT8	*SLC35C2*	0.014591565	Co-expression	Dobbin-Giordano-2005
MMP8	*ZSWIM1*	0.014179909	Co-expression	Dobbin-Giordano-2005
MMP1	*MMP10*	0.0087530175	Co-expression	Dobbin-Giordano-2005
MMP3	*MMP10*	0.009577051	Co-expression	Dobbin-Giordano-2005
MMP3	*MMP1*	0.0071732434	Co-expression	Dobbin-Giordano-2005
MMP11	*MMP2*	0.012768546	Co-expression	Dobbin-Giordano-2005
MMP13	*MMP10*	0.011859682	Co-expression	Dobbin-Giordano-2005
MMP13	*MMP9*	0.015401816	Co-expression	Dobbin-Giordano-2005
MMP14	*MMP2*	0.010036182	Co-expression	Dobbin-Giordano-2005
MMP9	*MMP1*	0.010204952	Co-expression	Rosenwald-Staudt-2001
MMP9	*CD40*	0.0044214097	Co-expression	Rosenwald-Staudt-2001
MMP2	*MMP1*	0.014201451	Co-expression	Rosenwald-Staudt-2001
MMP2	*CD40*	0.0048950617	Co-expression	Rosenwald-Staudt-2001
MMP2	*MMP9*	0.005824463	Co-expression	Rosenwald-Staudt-2001
MMP15	*MMP9*	0.00978206	Co-expression	Rosenwald-Staudt-2001
MMP15	*MMP2*	0.010811945	Co-expression	Rosenwald-Staudt-2001
MMP12	*CD40*	0.0028690915	Co-expression	Rosenwald-Staudt-2001
MMP12	*MMP14*	0.0053635295	Co-expression	Rosenwald-Staudt-2001
MMP7	*ZSWIM3*	0.0129487	Co-expression	Rosenwald-Staudt-2001
MMP9	*MMP1*	0.008568336	Co-expression	Alizadeh-Staudt-2000
MMP9	*CD40*	0.006759039	Co-expression	Alizadeh-Staudt-2000
MMP2	*MMP1*	0.012227431	Co-expression	Alizadeh-Staudt-2000
MMP2	*CD40*	0.006280036	Co-expression	Alizadeh-Staudt-2000
MMP2	*MMP9*	0.0064525036	Co-expression	Alizadeh-Staudt-2000
MMP14	*MMP3*	0.02104439	Co-expression	Alizadeh-Staudt-2000
MMP15	*MMP9*	0.008582863	Co-expression	Alizadeh-Staudt-2000
MMP15	*MMP2*	0.009046634	Co-expression	Alizadeh-Staudt-2000
MMP7	*MMP3*	0.025251048	Co-expression	Alizadeh-Staudt-2000
MMP3	*MMP1*	0.03471106	Co-expression	Bild-Nevins-2006 B
MMP2	*MMP3*	0.010101463	Co-expression	Bild-Nevins-2006 B
MMP11	*MMP10*	0.011269141	Co-expression	Bild-Nevins-2006 B
MMP11	*MMP1*	0.01595287	Co-expression	Bild-Nevins-2006 B
MMP11	*MMP2*	0.009790911	Co-expression	Bild-Nevins-2006 B
MMP14	*MMP11*	0.015116542	Co-expression	Bild-Nevins-2006 B
MMP25	*MMP15*	0.007518016	Co-expression	Bild-Nevins-2006 B
MMP7	*MMP12*	0.009254638	Co-expression	Bild-Nevins-2006 B
MMP9	*MMP1*	0.023311744	Co-expression	Wang-Maris-2006
MMP2	*MMP10*	0.013783539	Co-expression	Wang-Maris-2006
MMP12	*MMP1*	0.03607238	Co-expression	Wang-Maris-2006
MMP12	*MMP9*	0.028320719	Co-expression	Wang-Maris-2006
MMP19	*MMP14*	0.010384828	Co-expression	Wang-Maris-2006
MMP20	*MMP8*	0.019756397	Co-expression	Wang-Maris-2006

MMP20	*MMP9*	0.016377661	Co-expression	Wang-Maris-2006
MMP1	*MMP27*	0.022062583	Co-expression	Chen-Brown-2002
MMP9	*MMP1*	0.014893332	Co-expression	Chen-Brown-2002
MMP12	*AYTL1*	0.018943634	Co-expression	Chen-Brown-2002
MMP12	*MMP9*	0.017586546	Co-expression	Chen-Brown-2002
MMP9	*MMP8*	0.028932996	Co-expression	Burington-Shaughnessy-2008
MMP24	*MMP11*	0.0022543943	Co-expression	Burington-Shaughnessy-2008
MMP25	*MMP2*	0.010628911	Co-expression	Burington-Shaughnessy-2008
MMP19	*MMP13*	0.009604596	Co-expression	Burington-Shaughnessy-2008
MMP20	*MMP11*	0.006200976	Co-expression	Burington-Shaughnessy-2008
HPX	*MMP25*	0.0112562915	Co-expression	Burington-Shaughnessy-2008
MMP9	*CD40*	0.006440802	Co-expression	Rieger-Chu-2004
MMP2	*ACOT8*	0.007743109	Co-expression	Rieger-Chu-2004
MMP25	*MMP11*	0.011598226	Co-expression	Rieger-Chu-2004
MMP12	*MMP11*	0.009452511	Co-expression	Rieger-Chu-2004
MMP19	*MMP3*	0.032761995	Co-expression	Rieger-Chu-2004
MMP9	*MMP1*	0.008684233	Co-expression	Wang-Cheung-2015
MMP12	*MMP3*	0.0019335776	Co-expression	Wang-Cheung-2015
MMP28	*MMP13*	0.016147366	Co-expression	Wang-Cheung-2015
MMP3	*MMP1*	0.004863176	Co-expression	Boldrick-Relman-2002
MMP12	*MMP3*	0.005013258	Co-expression	Boldrick-Relman-2002
ZSWIM3	*MMP15*	0.021783765	Co-expression	Boldrick-Relman-2002
MMP3	*MMP10*	0.02944486	Co-localization	Johnson-Shoemaker-2003
MMP9	*MMP8*	0.01102059	Co-localization	Johnson-Shoemaker-2003
MMP2	*MMP3*	0.0063566775	Co-localization	Johnson-Shoemaker-2003
PLTP	*SNX21*	0.018297002	Co-localization	Johnson-Shoemaker-2003
PLTP	*MMP2*	0.005005489	Co-localization	Johnson-Shoemaker-2003
MMP16	*MMP24*	0.012562963	Co-localization	Johnson-Shoemaker-2003
MMP14	*MMP10*	0.006571168	Co-localization	Johnson-Shoemaker-2003
MMP14	*MMP3*	0.007614681	Co-localization	Johnson-Shoemaker-2003
MMP14	*MMP2*	0.0035088009	Co-localization	Johnson-Shoemaker-2003
MMP14	*PLTP*	0.0065223807	Co-localization	Johnson-Shoemaker-2003
MMP19	*MMP14*	0.009298849	Co-localization	Johnson-Shoemaker-2003
MMP7	*MMP2*	0.0052156677	Co-localization	Johnson-Shoemaker-2003
MMP7	*MMP14*	0.006517967	Co-localization	Johnson-Shoemaker-2003
MMP3	*MMP10*	0.50150436	Physical Interactions	Huttlin-Gygi-2015
KLHL23	*ACOT8*	0.55343205	Physical Interactions	Huttlin-Gygi-2015
MMP8	*MMP10*	0.034334067	Physical Interactions	Vastrik-Stein-2007
MMP1	*MMP10*	0.022712572	Physical Interactions	Vastrik-Stein-2007
MMP3	*MMP10*	0.021534419	Physical Interactions	Vastrik-Stein-2007
MMP3	*MMP1*	0.01170476	Physical Interactions	Vastrik-Stein-2007
MMP9	*MMP10*	0.026511664	Physical Interactions	Vastrik-Stein-2007
MMP9	*MMP1*	0.014410078	Physical Interactions	Vastrik-Stein-2007
MMP2	*MMP10*	0.024194602	Physical Interactions	Vastrik-Stein-2007
MMP2	*MMP1*	0.013150669	Physical Interactions	Vastrik-Stein-2007
MMP2	*MMP3*	0.012468514	Physical Interactions	Vastrik-Stein-2007
MMP11	*MMP8*	0.034191784	Physical Interactions	Vastrik-Stein-2007

MMP11	*MMP1*	0.022618448	Physical Interactions	Vastrik-Stein-2007
MMP11	*MMP3*	0.02144518	Physical Interactions	Vastrik-Stein-2007
MMP11	*MMP9*	0.0264018	Physical Interactions	Vastrik-Stein-2007
MMP11	*MMP2*	0.02409434	Physical Interactions	Vastrik-Stein-2007
MMP13	*MMP10*	0.025822693	Physical Interactions	Vastrik-Stein-2007
MMP13	*MMP3*	0.013307539	Physical Interactions	Vastrik-Stein-2007
MMP13	*MMP11*	0.025715683	Physical Interactions	Vastrik-Stein-2007
MMP14	*MMP8*	0.026552005	Physical Interactions	Vastrik-Stein-2007
MMP14	*MMP1*	0.017564604	Physical Interactions	Vastrik-Stein-2007
MMP14	*MMP3*	0.016653487	Physical Interactions	Vastrik-Stein-2007
MMP14	*MMP9*	0.020502605	Physical Interactions	Vastrik-Stein-2007
MMP14	*MMP2*	0.018710723	Physical Interactions	Vastrik-Stein-2007
MMP14	*MMP13*	0.019969795	Physical Interactions	Vastrik-Stein-2007
MMP14	*MMP16*	0.13556261	Physical Interactions	Vastrik-Stein-2007
MMP15	*MMP8*	0.03837757	Physical Interactions	Vastrik-Stein-2007
MMP15	*MMP1*	0.025387421	Physical Interactions	Vastrik-Stein-2007
MMP15	*MMP3*	0.024070518	Physical Interactions	Vastrik-Stein-2007
MMP15	*MMP9*	0.02963393	Physical Interactions	Vastrik-Stein-2007
MMP15	*MMP2*	0.027043989	Physical Interactions	Vastrik-Stein-2007
MMP15	*MMP13*	0.02886382	Physical Interactions	Vastrik-Stein-2007
MMP12	*MMP10*	0.028728068	Physical Interactions	Vastrik-Stein-2007
MMP12	*MMP11*	0.028609017	Physical Interactions	Vastrik-Stein-2007
MMP12	*MMP14*	0.022216644	Physical Interactions	Vastrik-Stein-2007
MMP12	*MMP15*	0.032111358	Physical Interactions	Vastrik-Stein-2007
MMP19	*MMP10*	0.027956305	Physical Interactions	Vastrik-Stein-2007
MMP19	*MMP8*	0.022970373	Physical Interactions	Vastrik-Stein-2007
MMP19	*MMP1*	0.015195295	Physical Interactions	Vastrik-Stein-2007
MMP19	*MMP3*	0.014407081	Physical Interactions	Vastrik-Stein-2007
MMP19	*MMP9*	0.017736986	Physical Interactions	Vastrik-Stein-2007
MMP19	*MMP2*	0.016186813	Physical Interactions	Vastrik-Stein-2007
MMP19	*MMP11*	0.027840454	Physical Interactions	Vastrik-Stein-2007
MMP19	*MMP13*	0.017276047	Physical Interactions	Vastrik-Stein-2007
MMP19	*MMP14*	0.021619808	Physical Interactions	Vastrik-Stein-2007
MMP19	*MMP15*	0.031248705	Physical Interactions	Vastrik-Stein-2007
MMP19	*MMP12*	0.019219816	Physical Interactions	Vastrik-Stein-2007
MMP7	*MMP10*	0.02430869	Physical Interactions	Vastrik-Stein-2007
MMP7	*MMP1*	0.01321268	Physical Interactions	Vastrik-Stein-2007
MMP7	*MMP3*	0.012527309	Physical Interactions	Vastrik-Stein-2007
MMP7	*MMP2*	0.014074829	Physical Interactions	Vastrik-Stein-2007
MMP7	*MMP11*	0.024207955	Physical Interactions	Vastrik-Stein-2007
MMP7	*MMP14*	0.018798953	Physical Interactions	Vastrik-Stein-2007
MMP7	*MMP15*	0.027171515	Physical Interactions	Vastrik-Stein-2007
MMP7	*MMP19*	0.01626314	Physical Interactions	Vastrik-Stein-2007
MMP8	*MMP10*	0.034334067	Physical Interactions	IREF-reactome
MMP1	*MMP10*	0.022712572	Physical Interactions	IREF-reactome
MMP3	*MMP10*	0.021534419	Physical Interactions	IREF-reactome
MMP3	*MMP1*	0.01170476	Physical Interactions	IREF-reactome

MMP9	*MMP10*	0.026511664	Physical Interactions	IREF-reactome
MMP9	*MMP1*	0.014410078	Physical Interactions	IREF-reactome
MMP2	*MMP10*	0.024194602	Physical Interactions	IREF-reactome
MMP2	*MMP1*	0.013150669	Physical Interactions	IREF-reactome
MMP2	*MMP3*	0.012468514	Physical Interactions	IREF-reactome
MMP11	*MMP8*	0.034191784	Physical Interactions	IREF-reactome
MMP11	*MMP1*	0.022618448	Physical Interactions	IREF-reactome
MMP11	*MMP3*	0.02144518	Physical Interactions	IREF-reactome
MMP11	*MMP9*	0.0264018	Physical Interactions	IREF-reactome
MMP11	*MMP2*	0.02409434	Physical Interactions	IREF-reactome
MMP13	*MMP10*	0.025822693	Physical Interactions	IREF-reactome
MMP13	*MMP3*	0.013307539	Physical Interactions	IREF-reactome
MMP13	*MMP11*	0.025715683	Physical Interactions	IREF-reactome
MMP14	*MMP8*	0.026552005	Physical Interactions	IREF-reactome
MMP14	*MMP1*	0.017564604	Physical Interactions	IREF-reactome
MMP14	*MMP3*	0.016653487	Physical Interactions	IREF-reactome
MMP14	*MMP9*	0.020502605	Physical Interactions	IREF-reactome
MMP14	*MMP2*	0.018710723	Physical Interactions	IREF-reactome
MMP14	*MMP13*	0.019969795	Physical Interactions	IREF-reactome
MMP14	*MMP16*	0.13556261	Physical Interactions	IREF-reactome
MMP15	*MMP8*	0.03837757	Physical Interactions	IREF-reactome
MMP15	*MMP1*	0.025387421	Physical Interactions	IREF-reactome
MMP15	*MMP3*	0.024070518	Physical Interactions	IREF-reactome
MMP15	*MMP9*	0.02963393	Physical Interactions	IREF-reactome
MMP15	*MMP2*	0.027043989	Physical Interactions	IREF-reactome
MMP15	*MMP13*	0.02886382	Physical Interactions	IREF-reactome
MMP12	*MMP10*	0.028728068	Physical Interactions	IREF-reactome
MMP12	*MMP11*	0.028609017	Physical Interactions	IREF-reactome
MMP12	*MMP14*	0.022216644	Physical Interactions	IREF-reactome
MMP12	*MMP15*	0.032111358	Physical Interactions	IREF-reactome
MMP19	*MMP10*	0.027956305	Physical Interactions	IREF-reactome
MMP19	*MMP8*	0.022970373	Physical Interactions	IREF-reactome
MMP19	*MMP1*	0.015195295	Physical Interactions	IREF-reactome
MMP19	*MMP3*	0.014407081	Physical Interactions	IREF-reactome
MMP19	*MMP9*	0.017736986	Physical Interactions	IREF-reactome
MMP19	*MMP2*	0.016186813	Physical Interactions	IREF-reactome
MMP19	*MMP11*	0.027840454	Physical Interactions	IREF-reactome
MMP19	*MMP13*	0.017276047	Physical Interactions	IREF-reactome
MMP19	*MMP14*	0.021619808	Physical Interactions	IREF-reactome
MMP19	*MMP15*	0.031248705	Physical Interactions	IREF-reactome
MMP19	*MMP12*	0.019219816	Physical Interactions	IREF-reactome
MMP7	*MMP10*	0.02430869	Physical Interactions	IREF-reactome
MMP7	*MMP1*	0.01321268	Physical Interactions	IREF-reactome
MMP7	*MMP3*	0.012527309	Physical Interactions	IREF-reactome
MMP7	*MMP2*	0.014074829	Physical Interactions	IREF-reactome
MMP7	*MMP11*	0.024207955	Physical Interactions	IREF-reactome
MMP7	*MMP14*	0.018798953	Physical Interactions	IREF-reactome

MMP7	*MMP15*	0.027171515	Physical Interactions	IREF-reactome
MMP7	*MMP19*	0.01626314	Physical Interactions	IREF-reactome
SLC35C2	*SYS1*	0.022186106	Physical Interactions	IREF-huri
SLC35C2	*SYS1*	0.021844983	Physical Interactions	Luck-Calderwood-2020
SLC35C2	*SYS1*	0.027210968	Physical Interactions	IREF-intact
MMP1	*MMP8*	0.13822381	Predicted	Wu-Stein-2010
MMP3	*MMP1*	0.07603955	Predicted	Wu-Stein-2010
MMP9	*MMP10*	0.28006592	Predicted	Wu-Stein-2010
MMP9	*MMP8*	0.06284516	Predicted	Wu-Stein-2010
MMP9	*MMP1*	0.033200886	Predicted	Wu-Stein-2010
MMP9	*MMP3*	0.034572322	Predicted	Wu-Stein-2010
MMP2	*MMP1*	0.029429162	Predicted	Wu-Stein-2010
MMP2	*MMP3*	0.030644799	Predicted	Wu-Stein-2010
MMP2	*MMP9*	0.013380332	Predicted	Wu-Stein-2010
MMP11	*MMP9*	0.17002596	Predicted	Wu-Stein-2010
MMP11	*MMP2*	0.15071048	Predicted	Wu-Stein-2010
MMP24	*MMP2*	0.25902104	Predicted	Wu-Stein-2010
MMP13	*MMP1*	0.08638272	Predicted	Wu-Stein-2010
MMP13	*MMP3*	0.08995094	Predicted	Wu-Stein-2010
MMP13	*MMP9*	0.03927497	Predicted	Wu-Stein-2010
MMP16	*MMP2*	0.12822069	Predicted	Wu-Stein-2010
MMP16	*MMP13*	0.37636313	Predicted	Wu-Stein-2010
MMP17	*MMP2*	0.25902104	Predicted	Wu-Stein-2010
MMP14	*MMP8*	0.10642476	Predicted	Wu-Stein-2010
MMP14	*MMP3*	0.058546286	Predicted	Wu-Stein-2010
MMP14	*MMP9*	0.025562862	Predicted	Wu-Stein-2010
MMP14	*MMP13*	0.066509955	Predicted	Wu-Stein-2010
MMP15	*MMP2*	0.08224454	Predicted	Wu-Stein-2010
MMP15	*MMP14*	0.15712658	Predicted	Wu-Stein-2010
MMP25	*MMP2*	0.115931414	Predicted	Wu-Stein-2010
MMP7	*MMP1*	0.10645419	Predicted	Wu-Stein-2010
MMP7	*MMP9*	0.04840071	Predicted	Wu-Stein-2010
MMP26	*MMP9*	0.061761584	Predicted	Wu-Stein-2010
MMP26	*MMP7*	0.19803023	Predicted	Wu-Stein-2010
WFDC3	*WFDC10B*	0.06809132	Shared protein domains	INTERPRO
MMP27	*MMP10*	0.04241342	Shared protein domains	INTERPRO
MMP8	*MMP10*	0.043842833	Shared protein domains	INTERPRO
MMP8	*MMP27*	0.039561167	Shared protein domains	INTERPRO
MMP1	*MMP10*	0.043842833	Shared protein domains	INTERPRO
MMP1	*MMP27*	0.039561167	Shared protein domains	INTERPRO
MMP1	*MMP8*	0.046538528	Shared protein domains	INTERPRO
MMP3	*MMP10*	0.043842833	Shared protein domains	INTERPRO
MMP3	*MMP27*	0.039561167	Shared protein domains	INTERPRO
MMP3	*MMP8*	0.046538528	Shared protein domains	INTERPRO
MMP3	*MMP1*	0.046538528	Shared protein domains	INTERPRO
MMP9	*MMP10*	0.038019557	Shared protein domains	INTERPRO
MMP9	*MMP27*	0.034306385	Shared protein domains	INTERPRO

MMP9	*MMP8*	0.04035738	Shared protein domains	INTERPRO
MMP9	*MMP1*	0.04035738	Shared protein domains	INTERPRO
MMP9	*MMP3*	0.04035738	Shared protein domains	INTERPRO
MMP2	*MMP10*	0.038019557	Shared protein domains	INTERPRO
MMP2	*MMP27*	0.034306385	Shared protein domains	INTERPRO
MMP2	*MMP8*	0.04035738	Shared protein domains	INTERPRO
MMP2	*MMP1*	0.04035738	Shared protein domains	INTERPRO
MMP2	*MMP3*	0.04035738	Shared protein domains	INTERPRO
MMP2	*MMP9*	0.04305101	Shared protein domains	INTERPRO
MMP11	*MMP10*	0.038830653	Shared protein domains	INTERPRO
MMP11	*MMP27*	0.03296157	Shared protein domains	INTERPRO
MMP11	*MMP8*	0.04295017	Shared protein domains	INTERPRO
MMP11	*MMP1*	0.04295017	Shared protein domains	INTERPRO
MMP11	*MMP3*	0.04295017	Shared protein domains	INTERPRO
MMP11	*MMP9*	0.037245277	Shared protein domains	INTERPRO
MMP11	*MMP2*	0.037245277	Shared protein domains	INTERPRO
MMP24	*MMP10*	0.038713504	Shared protein domains	INTERPRO
MMP24	*MMP27*	0.04090887	Shared protein domains	INTERPRO
MMP24	*MMP8*	0.041946262	Shared protein domains	INTERPRO
MMP24	*MMP1*	0.041946262	Shared protein domains	INTERPRO
MMP24	*MMP3*	0.041946262	Shared protein domains	INTERPRO
MMP24	*MMP9*	0.036374882	Shared protein domains	INTERPRO
MMP24	*MMP2*	0.036374882	Shared protein domains	INTERPRO
MMP24	*MMP11*	0.03704605	Shared protein domains	INTERPRO
MMP13	*MMP10*	0.043842833	Shared protein domains	INTERPRO
MMP13	*MMP27*	0.039561167	Shared protein domains	INTERPRO
MMP13	*MMP8*	0.046538528	Shared protein domains	INTERPRO
MMP13	*MMP1*	0.046538528	Shared protein domains	INTERPRO
MMP13	*MMP3*	0.046538528	Shared protein domains	INTERPRO
MMP13	*MMP9*	0.04035738	Shared protein domains	INTERPRO
MMP13	*MMP2*	0.04035738	Shared protein domains	INTERPRO
MMP13	*MMP11*	0.04295017	Shared protein domains	INTERPRO
MMP13	*MMP24*	0.041946262	Shared protein domains	INTERPRO
MMP16	*MMP10*	0.043031245	Shared protein domains	INTERPRO
MMP16	*MMP27*	0.038828816	Shared protein domains	INTERPRO
MMP16	*MMP8*	0.04567707	Shared protein domains	INTERPRO
MMP16	*MMP1*	0.04567707	Shared protein domains	INTERPRO
MMP16	*MMP3*	0.04567707	Shared protein domains	INTERPRO
MMP16	*MMP9*	0.039610337	Shared protein domains	INTERPRO
MMP16	*MMP2*	0.039610337	Shared protein domains	INTERPRO
MMP16	*MMP11*	0.04215508	Shared protein domains	INTERPRO
MMP16	*MMP24*	0.049316004	Shared protein domains	INTERPRO
MMP16	*MMP13*	0.04567707	Shared protein domains	INTERPRO
MMP17	*MMP10*	0.042559948	Shared protein domains	INTERPRO
MMP17	*MMP27*	0.044973437	Shared protein domains	INTERPRO
MMP17	*MMP8*	0.039697845	Shared protein domains	INTERPRO
MMP17	*MMP1*	0.039697845	Shared protein domains	INTERPRO

MMP17	*MMP3*	0.039697845	Shared protein domains INTERPRO
MMP17	*MMP9*	0.034424905	Shared protein domains INTERPRO
MMP17	*MMP2*	0.034424905	Shared protein domains INTERPRO
MMP17	*MMP11*	0.03307544	Shared protein domains INTERPRO
MMP17	*MMP24*	0.041050196	Shared protein domains INTERPRO
MMP17	*MMP13*	0.039697845	Shared protein domains INTERPRO
MMP17	*MMP16*	0.03896296	Shared protein domains INTERPRO
MMP14	*MMP10*	0.043031245	Shared protein domains INTERPRO
MMP14	*MMP27*	0.038828816	Shared protein domains INTERPRO
MMP14	*MMP8*	0.04567707	Shared protein domains INTERPRO
MMP14	*MMP1*	0.04567707	Shared protein domains INTERPRO
MMP14	*MMP3*	0.04567707	Shared protein domains INTERPRO
MMP14	*MMP9*	0.039610337	Shared protein domains INTERPRO
MMP14	*MMP2*	0.039610337	Shared protein domains INTERPRO
MMP14	*MMP11*	0.04215508	Shared protein domains INTERPRO
MMP14	*MMP24*	0.049316004	Shared protein domains INTERPRO
MMP14	*MMP13*	0.04567707	Shared protein domains INTERPRO
MMP14	*MMP16*	0.05256368	Shared protein domains INTERPRO
MMP14	*MMP17*	0.03896296	Shared protein domains INTERPRO
MMP15	*MMP10*	0.043031245	Shared protein domains INTERPRO
MMP15	*MMP27*	0.038828816	Shared protein domains INTERPRO
MMP15	*MMP8*	0.04567707	Shared protein domains INTERPRO
MMP15	*MMP1*	0.04567707	Shared protein domains INTERPRO
MMP15	*MMP3*	0.04567707	Shared protein domains INTERPRO
MMP15	*MMP9*	0.039610337	Shared protein domains INTERPRO
MMP15	*MMP2*	0.039610337	Shared protein domains INTERPRO
MMP15	*MMP11*	0.04215508	Shared protein domains INTERPRO
MMP15	*MMP24*	0.049316004	Shared protein domains INTERPRO
MMP15	*MMP13*	0.04567707	Shared protein domains INTERPRO
MMP15	*MMP16*	0.05256368	Shared protein domains INTERPRO
MMP15	*MMP17*	0.03896296	Shared protein domains INTERPRO
MMP15	*MMP14*	0.05256368	Shared protein domains INTERPRO
MMP25	*MMP10*	0.03976889	Shared protein domains INTERPRO
MMP25	*MMP27*	0.042024206	Shared protein domains INTERPRO
MMP25	*MMP8*	0.03709437	Shared protein domains INTERPRO
MMP25	*MMP1*	0.03709437	Shared protein domains INTERPRO
MMP25	*MMP3*	0.03709437	Shared protein domains INTERPRO
MMP25	*MMP9*	0.032167003	Shared protein domains INTERPRO
MMP25	*MMP2*	0.032167003	Shared protein domains INTERPRO
MMP25	*MMP11*	0.036435995	Shared protein domains INTERPRO
MMP25	*MMP24*	0.038358092	Shared protein domains INTERPRO
MMP25	*MMP13*	0.03709437	Shared protein domains INTERPRO
MMP25	*MMP16*	0.036407646	Shared protein domains INTERPRO
MMP25	*MMP17*	0.04216939	Shared protein domains INTERPRO
MMP25	*MMP14*	0.036407646	Shared protein domains INTERPRO
MMP25	*MMP15*	0.036407646	Shared protein domains INTERPRO
MMP21	*MMP10*	0.042432584	Shared protein domains INTERPRO

MMP21	*MMP27*	0.04483885	Shared protein domains	INTERPRO
MMP21	*MMP8*	0.039579045	Shared protein domains	INTERPRO
MMP21	*MMP1*	0.039579045	Shared protein domains	INTERPRO
MMP21	*MMP3*	0.039579045	Shared protein domains	INTERPRO
MMP21	*MMP9*	0.034321886	Shared protein domains	INTERPRO
MMP21	*MMP2*	0.034321886	Shared protein domains	INTERPRO
MMP21	*MMP11*	0.032976463	Shared protein domains	INTERPRO
MMP21	*MMP24*	0.040927354	Shared protein domains	INTERPRO
MMP21	*MMP13*	0.039579045	Shared protein domains	INTERPRO
MMP21	*MMP16*	0.03884636	Shared protein domains	INTERPRO
MMP21	*MMP17*	0.044993754	Shared protein domains	INTERPRO
MMP21	*MMP14*	0.03884636	Shared protein domains	INTERPRO
MMP21	*MMP15*	0.03884636	Shared protein domains	INTERPRO
MMP21	*MMP25*	0.042043194	Shared protein domains	INTERPRO
MMP12	*MMP10*	0.043842833	Shared protein domains	INTERPRO
MMP12	*MMP27*	0.039561167	Shared protein domains	INTERPRO
MMP12	*MMP8*	0.046538528	Shared protein domains	INTERPRO
MMP12	*MMP1*	0.046538528	Shared protein domains	INTERPRO
MMP12	*MMP3*	0.046538528	Shared protein domains	INTERPRO
MMP12	*MMP9*	0.04035738	Shared protein domains	INTERPRO
MMP12	*MMP2*	0.04035738	Shared protein domains	INTERPRO
MMP12	*MMP11*	0.04295017	Shared protein domains	INTERPRO
MMP12	*MMP24*	0.041946262	Shared protein domains	INTERPRO
MMP12	*MMP13*	0.046538528	Shared protein domains	INTERPRO
MMP12	*MMP16*	0.04567707	Shared protein domains	INTERPRO
MMP12	*MMP17*	0.039697845	Shared protein domains	INTERPRO
MMP12	*MMP14*	0.04567707	Shared protein domains	INTERPRO
MMP12	*MMP15*	0.04567707	Shared protein domains	INTERPRO
MMP12	*MMP25*	0.03709437	Shared protein domains	INTERPRO
MMP12	*MMP21*	0.039579045	Shared protein domains	INTERPRO
ZSWIM7	*ZSWIM1*	0.12318517	Shared protein domains	INTERPRO
MMP19	*MMP10*	0.042432584	Shared protein domains	INTERPRO
MMP19	*MMP27*	0.04483885	Shared protein domains	INTERPRO
MMP19	*MMP8*	0.039579045	Shared protein domains	INTERPRO
MMP19	*MMP1*	0.039579045	Shared protein domains	INTERPRO
MMP19	*MMP3*	0.039579045	Shared protein domains	INTERPRO
MMP19	*MMP9*	0.034321886	Shared protein domains	INTERPRO
MMP19	*MMP2*	0.034321886	Shared protein domains	INTERPRO
MMP19	*MMP11*	0.032976463	Shared protein domains	INTERPRO
MMP19	*MMP24*	0.040927354	Shared protein domains	INTERPRO
MMP19	*MMP13*	0.039579045	Shared protein domains	INTERPRO
MMP19	*MMP16*	0.03884636	Shared protein domains	INTERPRO
MMP19	*MMP17*	0.044993754	Shared protein domains	INTERPRO
MMP19	*MMP14*	0.03884636	Shared protein domains	INTERPRO
MMP19	*MMP15*	0.03884636	Shared protein domains	INTERPRO
MMP19	*MMP25*	0.042043194	Shared protein domains	INTERPRO
MMP19	*MMP21*	0.04485911	Shared protein domains	INTERPRO

MMP19	*MMP12*	0.039579045	Shared protein domains	INTERPRO
MMP28	*MMP10*	0.042430583	Shared protein domains	INTERPRO
MMP28	*MMP27*	0.044836733	Shared protein domains	INTERPRO
MMP28	*MMP8*	0.039577175	Shared protein domains	INTERPRO
MMP28	*MMP1*	0.039577175	Shared protein domains	INTERPRO
MMP28	*MMP3*	0.039577175	Shared protein domains	INTERPRO
MMP28	*MMP9*	0.034320265	Shared protein domains	INTERPRO
MMP28	*MMP2*	0.034320265	Shared protein domains	INTERPRO
MMP28	*MMP11*	0.032974906	Shared protein domains	INTERPRO
MMP28	*MMP24*	0.040925417	Shared protein domains	INTERPRO
MMP28	*MMP13*	0.039577175	Shared protein domains	INTERPRO
MMP28	*MMP16*	0.038844526	Shared protein domains	INTERPRO
MMP28	*MMP17*	0.044991635	Shared protein domains	INTERPRO
MMP28	*MMP14*	0.038844526	Shared protein domains	INTERPRO
MMP28	*MMP15*	0.038844526	Shared protein domains	INTERPRO
MMP28	*MMP25*	0.04204121	Shared protein domains	INTERPRO
MMP28	*MMP21*	0.04485699	Shared protein domains	INTERPRO
MMP28	*MMP12*	0.039577175	Shared protein domains	INTERPRO
MMP28	*MMP19*	0.04485699	Shared protein domains	INTERPRO
MMP20	*MMP10*	0.042542666	Shared protein domains	INTERPRO
MMP20	*MMP27*	0.044955175	Shared protein domains	INTERPRO
MMP20	*MMP8*	0.03968172	Shared protein domains	INTERPRO
MMP20	*MMP1*	0.03968172	Shared protein domains	INTERPRO
MMP20	*MMP3*	0.03968172	Shared protein domains	INTERPRO
MMP20	*MMP9*	0.034410927	Shared protein domains	INTERPRO
MMP20	*MMP2*	0.034410927	Shared protein domains	INTERPRO
MMP20	*MMP11*	0.03306201	Shared protein domains	INTERPRO
MMP20	*MMP24*	0.04103353	Shared protein domains	INTERPRO
MMP20	*MMP13*	0.03968172	Shared protein domains	INTERPRO
MMP20	*MMP16*	0.038947135	Shared protein domains	INTERPRO
MMP20	*MMP17*	0.045110483	Shared protein domains	INTERPRO
MMP20	*MMP14*	0.038947135	Shared protein domains	INTERPRO
MMP20	*MMP15*	0.038947135	Shared protein domains	INTERPRO
MMP20	*MMP25*	0.042152263	Shared protein domains	INTERPRO
MMP20	*MMP21*	0.044975486	Shared protein domains	INTERPRO
MMP20	*MMP12*	0.03968172	Shared protein domains	INTERPRO
MMP20	*MMP19*	0.044975486	Shared protein domains	INTERPRO
MMP20	*MMP28*	0.044973362	Shared protein domains	INTERPRO
ZSWIM3	*ZSWIM1*	0.12318487	Shared protein domains	INTERPRO
ZSWIM3	*ZSWIM7*	0.12318487	Shared protein domains	INTERPRO
AC002094.	*MMP10*	0.029944472	Shared protein domains	INTERPRO
AC002094.	*MMP27*	0.031643704	Shared protein domains	INTERPRO
AC002094.	*MMP8*	0.027929543	Shared protein domains	INTERPRO
AC002094.	*MMP1*	0.027929543	Shared protein domains	INTERPRO
AC002094.	*MMP3*	0.027929543	Shared protein domains	INTERPRO
AC002094.	*MMP9*	0.024217188	Shared protein domains	INTERPRO
AC002094.	*MMP2*	0.024217188	Shared protein domains	INTERPRO

AC002094..	*MMP11*	0.033313278	Shared protein domains	INTERPRO
AC002094..	*MMP24*	0.028881663	Shared protein domains	INTERPRO
AC002094..	*MMP13*	0.027929543	Shared protein domains	INTERPRO
AC002094..	*MMP16*	0.02741216	Shared protein domains	INTERPRO
AC002094..	*MMP17*	0.031753026	Shared protein domains	INTERPRO
AC002094..	*MMP14*	0.02741216	Shared protein domains	INTERPRO
AC002094..	*MMP15*	0.02741216	Shared protein domains	INTERPRO
AC002094..	*MMP25*	0.034980737	Shared protein domains	INTERPRO
AC002094..	*MMP21*	0.031658	Shared protein domains	INTERPRO
AC002094..	*MMP12*	0.027929543	Shared protein domains	INTERPRO
AC002094..	*MMP19*	0.031658	Shared protein domains	INTERPRO
AC002094..	*MMP28*	0.031656504	Shared protein domains	INTERPRO
AC002094..	*MMP20*	0.03174013	Shared protein domains	INTERPRO
MMP7	*MMP10*	0.04021619	Shared protein domains	INTERPRO
MMP7	*MMP27*	0.042497	Shared protein domains	INTERPRO
MMP7	*MMP8*	0.037511457	Shared protein domains	INTERPRO
MMP7	*MMP1*	0.037511457	Shared protein domains	INTERPRO
MMP7	*MMP3*	0.037511457	Shared protein domains	INTERPRO
MMP7	*MMP9*	0.0325284	Shared protein domains	INTERPRO
MMP7	*MMP2*	0.0325284	Shared protein domains	INTERPRO
MMP7	*MMP11*	0.025982523	Shared protein domains	INTERPRO
MMP7	*MMP24*	0.038789466	Shared protein domains	INTERPRO
MMP7	*MMP13*	0.037511457	Shared protein domains	INTERPRO
MMP7	*MMP16*	0.036816973	Shared protein domains	INTERPRO
MMP7	*MMP17*	0.04264382	Shared protein domains	INTERPRO
MMP7	*MMP14*	0.036816973	Shared protein domains	INTERPRO
MMP7	*MMP15*	0.036816973	Shared protein domains	INTERPRO
MMP7	*MMP25*	0.03706057	Shared protein domains	INTERPRO
MMP7	*MMP21*	0.042516205	Shared protein domains	INTERPRO
MMP7	*MMP12*	0.037511457	Shared protein domains	INTERPRO
MMP7	*MMP19*	0.042516205	Shared protein domains	INTERPRO
MMP7	*MMP28*	0.042514194	Shared protein domains	INTERPRO
MMP7	*MMP20*	0.0426265	Shared protein domains	INTERPRO
MMP26	*MMP10*	0.03241773	Shared protein domains	INTERPRO
MMP26	*MMP27*	0.034256745	Shared protein domains	INTERPRO
MMP26	*MMP8*	0.030236969	Shared protein domains	INTERPRO
MMP26	*MMP1*	0.030236969	Shared protein domains	INTERPRO
MMP26	*MMP3*	0.030236969	Shared protein domains	INTERPRO
MMP26	*MMP9*	0.026219176	Shared protein domains	INTERPRO
MMP26	*MMP2*	0.026219176	Shared protein domains	INTERPRO
MMP26	*MMP11*	0.03606374	Shared protein domains	INTERPRO
MMP26	*MMP24*	0.03126742	Shared protein domains	INTERPRO
MMP26	*MMP13*	0.030236969	Shared protein domains	INTERPRO
MMP26	*MMP16*	0.029677015	Shared protein domains	INTERPRO
MMP26	*MMP17*	0.034375094	Shared protein domains	INTERPRO
MMP26	*MMP14*	0.029677015	Shared protein domains	INTERPRO
MMP26	*MMP15*	0.029677015	Shared protein domains	INTERPRO

MMP26	*MMP25*	0.03786844	Shared protein domains	INTERPRO
MMP26	*MMP21*	0.034272224	Shared protein domains	INTERPRO
MMP26	*MMP12*	0.030236969	Shared protein domains	INTERPRO
MMP26	*MMP19*	0.034272224	Shared protein domains	INTERPRO
MMP26	*MMP28*	0.034270607	Shared protein domains	INTERPRO
MMP26	*MMP20*	0.034361135	Shared protein domains	INTERPRO
MMP26	*MMP7*	0.045187496	Shared protein domains	INTERPRO
HPX	*MMP10*	0.02392443	Shared protein domains	INTERPRO
HPX	*MMP27*	0.025282046	Shared protein domains	INTERPRO
HPX	*MMP8*	0.035150975	Shared protein domains	INTERPRO
HPX	*MMP1*	0.035150975	Shared protein domains	INTERPRO
HPX	*MMP3*	0.035150975	Shared protein domains	INTERPRO
HPX	*MMP9*	0.0304804	Shared protein domains	INTERPRO
HPX	*MMP2*	0.0304804	Shared protein domains	INTERPRO
HPX	*MMP11*	0.041924458	Shared protein domains	INTERPRO
HPX	*MMP24*	0.036348853	Shared protein domains	INTERPRO
HPX	*MMP13*	0.035150975	Shared protein domains	INTERPRO
HPX	*MMP16*	0.034500044	Shared protein domains	INTERPRO
HPX	*MMP17*	0.025369389	Shared protein domains	INTERPRO
HPX	*MMP14*	0.034500044	Shared protein domains	INTERPRO
HPX	*MMP15*	0.034500044	Shared protein domains	INTERPRO
HPX	*MMP25*	0.0279482	Shared protein domains	INTERPRO
HPX	*MMP21*	0.02529347	Shared protein domains	INTERPRO
HPX	*MMP12*	0.035150975	Shared protein domains	INTERPRO
HPX	*MMP19*	0.02529347	Shared protein domains	INTERPRO
HPX	*MMP28*	0.025292275	Shared protein domains	INTERPRO
HPX	*MMP20*	0.025359085	Shared protein domains	INTERPRO
HPX	*AC002094.*	0.06355177	Shared protein domains	INTERPRO
MMP27	*MMP10*	0.044638466	Shared protein domains	PFAM
MMP8	*MMP10*	0.044638466	Shared protein domains	PFAM
MMP8	*MMP27*	0.044638466	Shared protein domains	PFAM
MMP1	*MMP10*	0.044638466	Shared protein domains	PFAM
MMP1	*MMP27*	0.044638466	Shared protein domains	PFAM
MMP1	*MMP8*	0.044638466	Shared protein domains	PFAM
MMP3	*MMP10*	0.044638466	Shared protein domains	PFAM
MMP3	*MMP27*	0.044638466	Shared protein domains	PFAM
MMP3	*MMP8*	0.044638466	Shared protein domains	PFAM
MMP3	*MMP1*	0.044638466	Shared protein domains	PFAM
MMP9	*MMP10*	0.037137225	Shared protein domains	PFAM
MMP9	*MMP27*	0.037137225	Shared protein domains	PFAM
MMP9	*MMP8*	0.037137225	Shared protein domains	PFAM
MMP9	*MMP1*	0.037137225	Shared protein domains	PFAM
MMP9	*MMP3*	0.037137225	Shared protein domains	PFAM
MMP2	*MMP10*	0.037137225	Shared protein domains	PFAM
MMP2	*MMP27*	0.037137225	Shared protein domains	PFAM
MMP2	*MMP8*	0.037137225	Shared protein domains	PFAM
MMP2	*MMP1*	0.037137225	Shared protein domains	PFAM

MMP2	*MMP3*	0.037137225	Shared protein domains	PFAM
MMP2	*MMP9*	0.04289084	Shared protein domains	PFAM
MMP11	*MMP10*	0.038203504	Shared protein domains	PFAM
MMP11	*MMP27*	0.038203504	Shared protein domains	PFAM
MMP11	*MMP8*	0.038203504	Shared protein domains	PFAM
MMP11	*MMP1*	0.038203504	Shared protein domains	PFAM
MMP11	*MMP3*	0.038203504	Shared protein domains	PFAM
MMP11	*MMP9*	0.03178301	Shared protein domains	PFAM
MMP11	*MMP2*	0.03178301	Shared protein domains	PFAM
MMP24	*MMP10*	0.042370785	Shared protein domains	PFAM
MMP24	*MMP27*	0.042370785	Shared protein domains	PFAM
MMP24	*MMP8*	0.042370785	Shared protein domains	PFAM
MMP24	*MMP1*	0.042370785	Shared protein domains	PFAM
MMP24	*MMP3*	0.042370785	Shared protein domains	PFAM
MMP24	*MMP9*	0.03525061	Shared protein domains	PFAM
MMP24	*MMP2*	0.03525061	Shared protein domains	PFAM
MMP24	*MMP11*	0.036262486	Shared protein domains	PFAM
MMP13	*MMP10*	0.044638466	Shared protein domains	PFAM
MMP13	*MMP27*	0.044638466	Shared protein domains	PFAM
MMP13	*MMP8*	0.044638466	Shared protein domains	PFAM
MMP13	*MMP1*	0.044638466	Shared protein domains	PFAM
MMP13	*MMP3*	0.044638466	Shared protein domains	PFAM
MMP13	*MMP9*	0.037137225	Shared protein domains	PFAM
MMP13	*MMP2*	0.037137225	Shared protein domains	PFAM
MMP13	*MMP11*	0.038203504	Shared protein domains	PFAM
MMP13	*MMP24*	0.042370785	Shared protein domains	PFAM
MMP16	*MMP10*	0.042370785	Shared protein domains	PFAM
MMP16	*MMP27*	0.042370785	Shared protein domains	PFAM
MMP16	*MMP8*	0.042370785	Shared protein domains	PFAM
MMP16	*MMP1*	0.042370785	Shared protein domains	PFAM
MMP16	*MMP3*	0.042370785	Shared protein domains	PFAM
MMP16	*MMP9*	0.03525061	Shared protein domains	PFAM
MMP16	*MMP2*	0.03525061	Shared protein domains	PFAM
MMP16	*MMP11*	0.036262486	Shared protein domains	PFAM
MMP16	*MMP24*	0.06165466	Shared protein domains	PFAM
MMP16	*MMP13*	0.042370785	Shared protein domains	PFAM
MMP17	*MMP10*	0.044638466	Shared protein domains	PFAM
MMP17	*MMP27*	0.044638466	Shared protein domains	PFAM
MMP17	*MMP8*	0.044638466	Shared protein domains	PFAM
MMP17	*MMP1*	0.044638466	Shared protein domains	PFAM
MMP17	*MMP3*	0.044638466	Shared protein domains	PFAM
MMP17	*MMP9*	0.037137225	Shared protein domains	PFAM
MMP17	*MMP2*	0.037137225	Shared protein domains	PFAM
MMP17	*MMP11*	0.038203504	Shared protein domains	PFAM
MMP17	*MMP24*	0.042370785	Shared protein domains	PFAM
MMP17	*MMP13*	0.044638466	Shared protein domains	PFAM
MMP17	*MMP16*	0.042370785	Shared protein domains	PFAM

MMP14	*MMP10*	0.042370785	Shared protein domains	PFAM
MMP14	*MMP27*	0.042370785	Shared protein domains	PFAM
MMP14	*MMP8*	0.042370785	Shared protein domains	PFAM
MMP14	*MMP1*	0.042370785	Shared protein domains	PFAM
MMP14	*MMP3*	0.042370785	Shared protein domains	PFAM
MMP14	*MMP9*	0.03525061	Shared protein domains	PFAM
MMP14	*MMP2*	0.03525061	Shared protein domains	PFAM
MMP14	*MMP11*	0.036262486	Shared protein domains	PFAM
MMP14	*MMP24*	0.06165466	Shared protein domains	PFAM
MMP14	*MMP13*	0.042370785	Shared protein domains	PFAM
MMP14	*MMP16*	0.06165466	Shared protein domains	PFAM
MMP14	*MMP17*	0.042370785	Shared protein domains	PFAM
MMP15	*MMP10*	0.042370785	Shared protein domains	PFAM
MMP15	*MMP27*	0.042370785	Shared protein domains	PFAM
MMP15	*MMP8*	0.042370785	Shared protein domains	PFAM
MMP15	*MMP1*	0.042370785	Shared protein domains	PFAM
MMP15	*MMP3*	0.042370785	Shared protein domains	PFAM
MMP15	*MMP9*	0.03525061	Shared protein domains	PFAM
MMP15	*MMP2*	0.03525061	Shared protein domains	PFAM
MMP15	*MMP11*	0.036262486	Shared protein domains	PFAM
MMP15	*MMP24*	0.06165466	Shared protein domains	PFAM
MMP15	*MMP13*	0.042370785	Shared protein domains	PFAM
MMP15	*MMP16*	0.06165466	Shared protein domains	PFAM
MMP15	*MMP17*	0.042370785	Shared protein domains	PFAM
MMP15	*MMP14*	0.06165466	Shared protein domains	PFAM
MMP25	*MMP10*	0.044638466	Shared protein domains	PFAM
MMP25	*MMP27*	0.044638466	Shared protein domains	PFAM
MMP25	*MMP8*	0.044638466	Shared protein domains	PFAM
MMP25	*MMP1*	0.044638466	Shared protein domains	PFAM
MMP25	*MMP3*	0.044638466	Shared protein domains	PFAM
MMP25	*MMP9*	0.037137225	Shared protein domains	PFAM
MMP25	*MMP2*	0.037137225	Shared protein domains	PFAM
MMP25	*MMP11*	0.038203504	Shared protein domains	PFAM
MMP25	*MMP24*	0.042370785	Shared protein domains	PFAM
MMP25	*MMP13*	0.044638466	Shared protein domains	PFAM
MMP25	*MMP16*	0.042370785	Shared protein domains	PFAM
MMP25	*MMP17*	0.044638466	Shared protein domains	PFAM
MMP25	*MMP14*	0.042370785	Shared protein domains	PFAM
MMP25	*MMP15*	0.042370785	Shared protein domains	PFAM
MMP21	*MMP10*	0.044638466	Shared protein domains	PFAM
MMP21	*MMP27*	0.044638466	Shared protein domains	PFAM
MMP21	*MMP8*	0.044638466	Shared protein domains	PFAM
MMP21	*MMP1*	0.044638466	Shared protein domains	PFAM
MMP21	*MMP3*	0.044638466	Shared protein domains	PFAM
MMP21	*MMP9*	0.037137225	Shared protein domains	PFAM
MMP21	*MMP2*	0.037137225	Shared protein domains	PFAM
MMP21	*MMP11*	0.038203504	Shared protein domains	PFAM

MMP21	*MMP24*	0.042370785	Shared protein domains	PFAM
MMP21	*MMP13*	0.044638466	Shared protein domains	PFAM
MMP21	*MMP16*	0.042370785	Shared protein domains	PFAM
MMP21	*MMP17*	0.044638466	Shared protein domains	PFAM
MMP21	*MMP14*	0.042370785	Shared protein domains	PFAM
MMP21	*MMP15*	0.042370785	Shared protein domains	PFAM
MMP21	*MMP25*	0.044638466	Shared protein domains	PFAM
MMP12	*MMP10*	0.044638466	Shared protein domains	PFAM
MMP12	*MMP27*	0.044638466	Shared protein domains	PFAM
MMP12	*MMP8*	0.044638466	Shared protein domains	PFAM
MMP12	*MMP1*	0.044638466	Shared protein domains	PFAM
MMP12	*MMP3*	0.044638466	Shared protein domains	PFAM
MMP12	*MMP9*	0.037137225	Shared protein domains	PFAM
MMP12	*MMP2*	0.037137225	Shared protein domains	PFAM
MMP12	*MMP11*	0.038203504	Shared protein domains	PFAM
MMP12	*MMP24*	0.042370785	Shared protein domains	PFAM
MMP12	*MMP13*	0.044638466	Shared protein domains	PFAM
MMP12	*MMP16*	0.042370785	Shared protein domains	PFAM
MMP12	*MMP17*	0.044638466	Shared protein domains	PFAM
MMP12	*MMP14*	0.042370785	Shared protein domains	PFAM
MMP12	*MMP15*	0.042370785	Shared protein domains	PFAM
MMP12	*MMP25*	0.044638466	Shared protein domains	PFAM
MMP12	*MMP21*	0.044638466	Shared protein domains	PFAM
ZSWIM7	*ZSWIM1*	0.43098608	Shared protein domains	PFAM
MMP19	*MMP10*	0.044638466	Shared protein domains	PFAM
MMP19	*MMP27*	0.044638466	Shared protein domains	PFAM
MMP19	*MMP8*	0.044638466	Shared protein domains	PFAM
MMP19	*MMP1*	0.044638466	Shared protein domains	PFAM
MMP19	*MMP3*	0.044638466	Shared protein domains	PFAM
MMP19	*MMP9*	0.037137225	Shared protein domains	PFAM
MMP19	*MMP2*	0.037137225	Shared protein domains	PFAM
MMP19	*MMP11*	0.038203504	Shared protein domains	PFAM
MMP19	*MMP24*	0.042370785	Shared protein domains	PFAM
MMP19	*MMP13*	0.044638466	Shared protein domains	PFAM
MMP19	*MMP16*	0.042370785	Shared protein domains	PFAM
MMP19	*MMP17*	0.044638466	Shared protein domains	PFAM
MMP19	*MMP14*	0.042370785	Shared protein domains	PFAM
MMP19	*MMP15*	0.042370785	Shared protein domains	PFAM
MMP19	*MMP25*	0.044638466	Shared protein domains	PFAM
MMP19	*MMP21*	0.044638466	Shared protein domains	PFAM
MMP19	*MMP12*	0.044638466	Shared protein domains	PFAM
MMP28	*MMP10*	0.044638466	Shared protein domains	PFAM
MMP28	*MMP27*	0.044638466	Shared protein domains	PFAM
MMP28	*MMP8*	0.044638466	Shared protein domains	PFAM
MMP28	*MMP1*	0.044638466	Shared protein domains	PFAM
MMP28	*MMP3*	0.044638466	Shared protein domains	PFAM
MMP28	*MMP9*	0.037137225	Shared protein domains	PFAM

MMP28	*MMP2*	0.037137225	Shared protein domains	PFAM
MMP28	*MMP11*	0.038203504	Shared protein domains	PFAM
MMP28	*MMP24*	0.042370785	Shared protein domains	PFAM
MMP28	*MMP13*	0.044638466	Shared protein domains	PFAM
MMP28	*MMP16*	0.042370785	Shared protein domains	PFAM
MMP28	*MMP17*	0.044638466	Shared protein domains	PFAM
MMP28	*MMP14*	0.042370785	Shared protein domains	PFAM
MMP28	*MMP15*	0.042370785	Shared protein domains	PFAM
MMP28	*MMP25*	0.044638466	Shared protein domains	PFAM
MMP28	*MMP21*	0.044638466	Shared protein domains	PFAM
MMP28	*MMP12*	0.044638466	Shared protein domains	PFAM
MMP28	*MMP19*	0.044638466	Shared protein domains	PFAM
MMP20	*MMP10*	0.044638466	Shared protein domains	PFAM
MMP20	*MMP27*	0.044638466	Shared protein domains	PFAM
MMP20	*MMP8*	0.044638466	Shared protein domains	PFAM
MMP20	*MMP1*	0.044638466	Shared protein domains	PFAM
MMP20	*MMP3*	0.044638466	Shared protein domains	PFAM
MMP20	*MMP9*	0.037137225	Shared protein domains	PFAM
MMP20	*MMP2*	0.037137225	Shared protein domains	PFAM
MMP20	*MMP11*	0.038203504	Shared protein domains	PFAM
MMP20	*MMP24*	0.042370785	Shared protein domains	PFAM
MMP20	*MMP13*	0.044638466	Shared protein domains	PFAM
MMP20	*MMP16*	0.042370785	Shared protein domains	PFAM
MMP20	*MMP17*	0.044638466	Shared protein domains	PFAM
MMP20	*MMP14*	0.042370785	Shared protein domains	PFAM
MMP20	*MMP15*	0.042370785	Shared protein domains	PFAM
MMP20	*MMP25*	0.044638466	Shared protein domains	PFAM
MMP20	*MMP21*	0.044638466	Shared protein domains	PFAM
MMP20	*MMP12*	0.044638466	Shared protein domains	PFAM
MMP20	*MMP19*	0.044638466	Shared protein domains	PFAM
MMP20	*MMP28*	0.044638466	Shared protein domains	PFAM
ZSWIM3	*ZSWIM1*	0.43098608	Shared protein domains	PFAM
ZSWIM3	*ZSWIM7*	0.43098608	Shared protein domains	PFAM
AC002094.	*MMP10*	0.031686947	Shared protein domains	PFAM
AC002094.	*MMP27*	0.031686947	Shared protein domains	PFAM
AC002094.	*MMP8*	0.031686947	Shared protein domains	PFAM
AC002094.	*MMP1*	0.031686947	Shared protein domains	PFAM
AC002094.	*MMP3*	0.031686947	Shared protein domains	PFAM
AC002094.	*MMP9*	0.026360147	Shared protein domains	PFAM
AC002094.	*MMP2*	0.026360147	Shared protein domains	PFAM
AC002094.	*MMP11*	0.041484844	Shared protein domains	PFAM
AC002094.	*MMP24*	0.030076455	Shared protein domains	PFAM
AC002094.	*MMP13*	0.031686947	Shared protein domains	PFAM
AC002094.	*MMP16*	0.030076455	Shared protein domains	PFAM
AC002094.	*MMP17*	0.031686947	Shared protein domains	PFAM
AC002094.	*MMP14*	0.030076455	Shared protein domains	PFAM
AC002094.	*MMP15*	0.030076455	Shared protein domains	PFAM

AC002094..	*MMP25*	0.031686947	Shared protein domains	PFAM
AC002094..	*MMP21*	0.031686947	Shared protein domains	PFAM
AC002094..	*MMP12*	0.031686947	Shared protein domains	PFAM
AC002094..	*MMP19*	0.031686947	Shared protein domains	PFAM
AC002094..	*MMP28*	0.031686947	Shared protein domains	PFAM
AC002094..	*MMP20*	0.031686947	Shared protein domains	PFAM
MMP7	*MMP10*	0.04232532	Shared protein domains	PFAM
MMP7	*MMP27*	0.04232532	Shared protein domains	PFAM
MMP7	*MMP8*	0.04232532	Shared protein domains	PFAM
MMP7	*MMP1*	0.04232532	Shared protein domains	PFAM
MMP7	*MMP3*	0.04232532	Shared protein domains	PFAM
MMP7	*MMP9*	0.035212174	Shared protein domains	PFAM
MMP7	*MMP2*	0.035212174	Shared protein domains	PFAM
MMP7	*MMP11*	0.026986677	Shared protein domains	PFAM
MMP7	*MMP24*	0.040174913	Shared protein domains	PFAM
MMP7	*MMP13*	0.04232532	Shared protein domains	PFAM
MMP7	*MMP16*	0.040174913	Shared protein domains	PFAM
MMP7	*MMP17*	0.04232532	Shared protein domains	PFAM
MMP7	*MMP14*	0.040174913	Shared protein domains	PFAM
MMP7	*MMP15*	0.040174913	Shared protein domains	PFAM
MMP7	*MMP25*	0.04232532	Shared protein domains	PFAM
MMP7	*MMP21*	0.04232532	Shared protein domains	PFAM
MMP7	*MMP12*	0.04232532	Shared protein domains	PFAM
MMP7	*MMP19*	0.04232532	Shared protein domains	PFAM
MMP7	*MMP28*	0.04232532	Shared protein domains	PFAM
MMP7	*MMP20*	0.04232532	Shared protein domains	PFAM
MMP26	*MMP10*	0.03326784	Shared protein domains	PFAM
MMP26	*MMP27*	0.03326784	Shared protein domains	PFAM
MMP26	*MMP8*	0.03326784	Shared protein domains	PFAM
MMP26	*MMP1*	0.03326784	Shared protein domains	PFAM
MMP26	*MMP3*	0.03326784	Shared protein domains	PFAM
MMP26	*MMP9*	0.027675318	Shared protein domains	PFAM
MMP26	*MMP2*	0.027675318	Shared protein domains	PFAM
MMP26	*MMP11*	0.043554474	Shared protein domains	PFAM
MMP26	*MMP24*	0.031577017	Shared protein domains	PFAM
MMP26	*MMP13*	0.03326784	Shared protein domains	PFAM
MMP26	*MMP16*	0.031577017	Shared protein domains	PFAM
MMP26	*MMP17*	0.03326784	Shared protein domains	PFAM
MMP26	*MMP14*	0.031577017	Shared protein domains	PFAM
MMP26	*MMP15*	0.031577017	Shared protein domains	PFAM
MMP26	*MMP25*	0.03326784	Shared protein domains	PFAM
MMP26	*MMP21*	0.03326784	Shared protein domains	PFAM
MMP26	*MMP12*	0.03326784	Shared protein domains	PFAM
MMP26	*MMP19*	0.03326784	Shared protein domains	PFAM
MMP26	*MMP28*	0.03326784	Shared protein domains	PFAM
MMP26	*MMP20*	0.03326784	Shared protein domains	PFAM
MMP26	*MMP7*	0.04674424	Shared protein domains	PFAM

HPX	*MMP10*	0.031686947	Shared protein domains	PFAM
HPX	*MMP27*	0.031686947	Shared protein domains	PFAM
HPX	*MMP8*	0.031686947	Shared protein domains	PFAM
HPX	*MMP1*	0.031686947	Shared protein domains	PFAM
HPX	*MMP3*	0.031686947	Shared protein domains	PFAM
HPX	*MMP9*	0.026360147	Shared protein domains	PFAM
HPX	*MMP2*	0.026360147	Shared protein domains	PFAM
HPX	*MMP11*	0.041484844	Shared protein domains	PFAM
HPX	*MMP24*	0.030076455	Shared protein domains	PFAM
HPX	*MMP13*	0.031686947	Shared protein domains	PFAM
HPX	*MMP16*	0.030076455	Shared protein domains	PFAM
HPX	*MMP17*	0.031686947	Shared protein domains	PFAM
HPX	*MMP14*	0.030076455	Shared protein domains	PFAM
HPX	*MMP15*	0.030076455	Shared protein domains	PFAM
HPX	*MMP25*	0.031686947	Shared protein domains	PFAM
HPX	*MMP21*	0.031686947	Shared protein domains	PFAM
HPX	*MMP12*	0.031686947	Shared protein domains	PFAM
HPX	*MMP19*	0.031686947	Shared protein domains	PFAM
HPX	*MMP28*	0.031686947	Shared protein domains	PFAM
HPX	*MMP20*	0.031686947	Shared protein domains	PFAM
HPX	*AC002094.*	0.0692488	Shared protein domains	PFAM

ciated genes (functionality in various tissue/organs) (GeneMANIA) (http://genemania.org

;)
