# Supplementary material for: Matrix Metalloproteinase Gene Polymorphisms Are Associated with Breast Cancer in the Caucasian Women of Russia
Source: Int J Mol Sci. 2022 Oct 20;23(20):12638. doi: 10.3390/ijms232012638 (PMC9604098; doi:10.3390/ijms232012638)
Supplement: Supplementary file 1 [file ijms-23-12638-s001.zip › Suppl Table 16.pdf]

Supplementary table S16. The regulatory potential of the studied SNPs
(HaploReg, v4.1, http://archive.broadinstitute.org/mammals/haploreg/haploreg.php)

chr	pos (hg38)	variant	Ref	Alt	AFR freq	AMR freq	ASN freq	EUR freq	GERP cons	SiPhy cons	Promoter histone marks	Enhancer histone marks
11	102722517	rs1940475	T	C	0.72	0.54	0.59	0.53				BLD
11	102799764	rs1799750	TC	T	0.55	0.44	0.33	0.49				6 tissues
11	102842889	rs679620	T	C	0.71	0.64	0.67	0.54				
16	55477894	rs243865	C	T	0.02	0.19	0.09	0.27			4 tissues	15 tissues
20	46007337	rs3918242	C	T	0.12	0.09	0.17	0.17				BLD, THYM, SPLN
20	46009497	rs3918249	T	C	0.56	0.31	0.72	0.39			4 tissues	BLD
20	46011586	rs17576	A	G	0.35	0.28	0.72	0.39			17 tissues	4 tissues
20	46013092	rs3787268	G	A	0.09	0.16	0.36	0.21			BLD, SKIN	BLD
20	46013767	rs2250889	G	C	0.82	0.80	0.76	0.95			24 tissues	
20	46014472	rs17577	G	A,C	0.17	0.10	0.18	0.18			21 tissues	SKIN

DNAse	Proteins bound	Motifs changed	NHGRI/EBI GWAS hits	GRASP QTL hits	Selected eQTL hits	GENCODE genes
BLD,BLD		CIZ,Myc,NF-AT1		1 hit	2 hits	*MMP8*
5 tissues	CFOS,GATA2	21 altered motifs			8 hits	*1.6kb 5' of MMP1*
		p300			3 hits	*MMP3*
ESDR,IPSC, MUS		Myf		1 hit	1 hit	*MMP2*
IPSC		4 altered motifs		1 hit	6 hits	*1.6kb 5' of MMP9*
BLD,BLD		4 altered motifs			9 hits	*MMP9*
ESC		Pax-4			12 hits	*MMP9*
BLD,BLD		6 altered motifs			4 hits	*MMP9*
15 tissues	CTCF	NRSF				*MMP9*
22 tissues	4 bound proteins			1 hit	6 hits	*MMP9*

RefSeq genes	dbSNP func annot
MMP8	missense
LOC100288077	intronic
MMP3	missense
1.3kb 5' of MMP2	
1.6kb 5' of MMP9	
MMP9	intronic
MMP9	missense
MMP9	intronic
MMP9	missense
MMP9	missense
