# Supplementary material for: Matrix Metalloproteinase Gene Polymorphisms Are Associated with Breast Cancer in the Caucasian Women of Russia
Source: Int J Mol Sci. 2022 Oct 20;23(20):12638. doi: 10.3390/ijms232012638 (PMC9604098; doi:10.3390/ijms232012638)
Supplement: Supplementary file 1 [file ijms-23-12638-s001.zip › Suppl table s1.pdf]

Supplementary table S1

The allele and genotype frequencies of the studied SNPs in the breast cancer and control groups

| Chr | SNP | Gene | Minor allele | Major allele | Minor allele frequency | Number of the studied chromosomes | Genotype distribution* | $H_o$ | $H_e$ | $P_{HWE}$ |
|---|---|---|---|---|---|---|---|---|---|---|
| Breast cancer patients (n=358) | | | | | | | | | | |
| 11 | rs1940475 | *MMP-8* | T | C | 0.482 | 710 | 76/190/89 | 0.535 | 0.499 | 0.202 |
| 11 | rs1799750 | *MMP-1* | 2G | 1G | 0.469 | 708 | 76/180/98 | 0.509 | 0.498 | 0.749 |
| 11 | rs679620 | *MMP-3* | T | C | 0.473 | 712 | 77/183/96 | 0.514 | 0.499 | 0.596 |
| 16 | rs243865 | *MMP-2* | T | C | 0.236 | 700 | 17/131/202 | 0.374 | 0.360 | 0.554 |
| 20 | rs3918242 | *MMP-9* | T | C | 0.170 | 708 | 15/90/249 | 0.254 | 0.282 | 0.086 |
| 20 | rs3918249 | *MMP-9* | C | T | 0.359 | 696 | 51/148/149 | 0.425 | 0.460 | 0.163 |
| 20 | rs17576 | *MMP-9* | G | A | 0.342 | 704 | 47/147/158 | 0.418 | 0.450 | 0.192 |
| 20 | rs3787268 | *MMP-9* | A | G | 0.233 | 700 | 23/117/210 | 0.334 | 0.357 | 0.232 |
| 20 | rs2250889 | *MMP-9* | G | C | 0.080 | 710 | 4/49/302 | 0.138 | 0.148 | 0.264 |
| 20 | rs17577 | *MMP-9* | A | G | 0.166 | 706 | 14/89/250 | 0.252 | 0.277 | 0.120 |
| Control group (n=746) | | | | | | | | | | |
| 11 | rs1940475 | *MMP-8* | T | C | 0.489 | 1482 | 194/337/210 | 0.455 | 0.500 | 0.015 |
| 11 | rs1799750 | *MMP-1* | 2G | 1G | 0.464 | 1446 | 168/335/220 | 0.463 | 0.497 | 0.073 |
| 11 | rs679620 | *MMP-3* | T | C | 0.499 | 1478 | 188/361/190 | 0.489 | 0.500 | 0.556 |
| 16 | rs243865 | *MMP-2* | T | C | 0.249 | 1464 | 52/261/419 | 0.357 | 0.374 | 0.200 |
| 20 | rs3918242 | *MMP-9* | T | C | 0.167 | 1470 | 18/209/508 | 0.284 | 0.278 | 0.596 |
| 20 | rs3918249 | *MMP-9* | C | T | 0.389 | 1470 | 113/346/276 | 0.471 | 0.475 | 0.816 |
| 20 | rs17576 | *MMP-9* | G | A | 0.389 | 1486 | 119/340/284 | 0.458 | 0.475 | 0.316 |
| 20 | rs3787268 | *MMP-9* | A | G | 0.215 | 1478 | 30/258/451 | 0.349 | 0.338 | 0.386 |
| 20 | rs2250889 | *MMP-9* | G | C | 0.110 | 1470 | 15/131/589 | 0.178 | 0.195 | 0.023 |
| 20 | rs17577 | *MMP-9* | A | G | 0.170 | 1452 | 20/207/499 | 0.285 | 0.282 | 0.896 |

Note: * minor allele homozygotes / heterozygotes / major allele homozygotes
