# Supplementary material for: Matrix Metalloproteinase Gene Polymorphisms Are Associated with Breast Cancer in the Caucasian Women of Russia
Source: Int J Mol Sci. 2022 Oct 20;23(20):12638. doi: 10.3390/ijms232012638 (PMC9604098; doi:10.3390/ijms232012638)
Supplement: Supplementary file 1 [file ijms-23-12638-s001.zip › Suppl table s10.pdf]

Supplementary table S10 Effect of SNPs in high LD (r2≥0.80) with the BC-associated polymorphisms of the *M*
(according to Genotype-Tissue Expression (GTEx) (http://www.gtexportal.org/)) (p <8.0E5, FDR≤0.05)

Chr	Lead SNP	Position lead SNP(hg38)	Proxy SNP	Position proxy SNP (hg38)	Distance (bp) between proxy and lead SNP	r2 between lead and proxy	D between lead and proxy
11	rs679620	102827993	rs526215	102807707	20286	0.82	-0.92
11	rs679620	102827993	rs1144396	102808321	19672	0.81	-0.92
11	rs679620	102827993	rs565082	102808927	19066	0.82	-0.92
11	rs679620	102827993	rs502174	102811772	16221	0.81	-0.91
11	rs679620	102827993	rs10791596	102816687	11306	0.83	-0.93
11	rs679620	102827993	rs2097248	102819770	8223	0.89	-0.96
11	rs679620	102827993	rs756544	102820431	7562	0.9	-0.96
11	rs679620	102827993	rs12804136	102820659	7334	0.91	-0.97
11	rs679620	102827993	rs11225434	102820751	7242	0.9	-0.97
11	rs679620	102827993	rs7117013	102821493	6500	0.91	-0.97
11	rs679620	102827993	rs10895364	102822132	5861	0.91	-0.97
11	rs679620	102827993	rs12801529	102822879	5114	0.92	-0.98
11	rs679620	102827993	rs10895365	102823462	4531	0.92	-0.98
11	rs679620	102827993	rs6590993	102825487	2506	0.89	-0.96
11	rs679620	102827993	rs6590994	102825901	2092	0.92	-0.98
11	rs679620	102827993	rs10895366	102826358	1635	0.96	-0.98
11	rs679620	102827993	rs7126392	102826390	1603	0.96	-0.98
11	rs679620	102827993	rs2212637	102826825	1168	0.94	-0.97
11	rs679620	102827993	rs12792871	102827605	388	0.96	-0.98
11	rs679620	102827993	rs1010698	102830290	-2297	0.98	-1
11	rs679620	102827993	rs2155013	102831127	-3134	0.98	-1
11	rs679620	102827993	rs4754884	102831532	-3539	0.98	-1
11	rs679620	102827993	rs635746	102832897	-4904	0.99	1
11	rs679620	102827993	rs633962	102833340	-5347	0.99	1
11	rs679620	102827993	rs666734	102833449	-5456	0.99	1
11	rs679620	102827993	rs595840	102834166	-6173	0.99	1
11	rs679620	102827993	rs639752	102836608	-8615	0.99	1
11	rs679620	102827993	rs575027	102837183	-9190	0.98	0.99
11	rs679620	102827993	rs520540	102838694	-10701	0.98	0.99
11	rs679620	102827993	rs591058	102840607	-12614	1	1
11	rs679620	102827993	rs602128	102842734	-14741	1	1
11	rs679620	102827993	rs679620	102842889	-14896	1	1
11	rs679620	102827993	rs678815	102843046	-15053	1	1
11	rs679620	102827993	rs617819	102843985	-15992	0.99	0.99
11	rs679620	102827993	rs632478	102844950	-16957	0.99	0.99
11	rs679620	102827993	rs645419	102845590	-17597	0.99	0.99
20	rs3918242	46007337	rs1888235	45995328	12009	0.95	0.98

20	rs3918242	46007337	rs6073984	46002014	5323	0.95	0.98
20	rs3918242	46007337	rs6065912	46003728	3609	0.86	0.98
20	rs3918242	46007337	rs3918241	46007096	241	1	1
20	rs3918242	46007337	rs2274755	46011053	-3716	0.99	1

20	rs3918242	46007337	rs2236416	46011936	-4599	0.99	1
20	rs3918242	46007337	rs17577	46014472	-7135	0.98	1
20	rs3918242	46007337	rs3918261	46014953	-7616	0.98	1
20	rs3918242	46007337	rs13925	46016326	-8989	0.98	1
20	rs3918242	46007337	rs3918270	46016700	-9363	0.96	1

20	rs3918242	46007337	rs3918271	46016954	-9617	0.93	0.97
20	rs3918242	46007337	rs66553311	46020003	-12666	0.82	1
20	rs3918242	46007337	rs10432735	46021679	-14342	0.89	0.98
20	rs3918249	46009497	rs6017721	45987758	21739	0.83	0.94
20	rs3918249	46009497	rs3848721	46005037	4460	0.94	0.99
20	rs3918249	46009497	rs3848722	46005181	4316	0.94	0.99
20	rs3918249	46009497	rs4810482	46005911	3586	0.9	0.96
20	rs3918249	46009497	rs3761158	46006273	3224	0.85	0.99

20	rs3918249	46009497	rs3761159	46006318	3179	0.95	0.99
20	rs3918249	46009497	rs6065913	46006464	3033	0.99	0.99
20	rs3918249	46009497	rs3918240	46006977	2520	0.99	0.99
20	rs3918249	46009497	rs3918251	46010142	-645	0.83	0.92
20	rs3918249	46009497	rs17576	46011586	-2089	0.99	0.99
20	rs3918249	46009497	rs13969	46014194	-4697	0.82	-0.98
20	rs17576	46011586	rs6017721	45987758	23828	0.82	0.94
20	rs17576	46011586	rs3848721	46005037	6549	0.92	0.99
20	rs17576	46011586	rs3848722	46005181	6405	0.92	0.99
20	rs17576	46011586	rs4810482	46005911	5675	0.89	0.96
20	rs17576	46011586	rs3761158	46006273	5313	0.84	0.99
20	rs17576	46011586	rs3761159	46006318	5268	0.94	0.99
20	rs17576	46011586	rs6065913	46006464	5122	0.99	0.98

20	rs17576	46011586	rs3918240	46006977	4609	0.99	0.98
20	rs17576	46011586	rs3918249	46009497	2089	0.99	0.99
20	rs17576	46011586	rs3918251	46010142	1444	0.84	0.93
20	rs17576	46011586	rs13969	46014194	-2608	0.83	-0.98
20	rs3787268	46013092	rs35610751	45991015	22077	0.87	0.94
20	rs3787268	46013092	rs6073980	45991065	22027	0.86	0.94
20	rs3787268	46013092	rs6073983	46001252	11840	0.89	0.98
20	rs3787268	46013092	rs3761157	46006183	6909	0.98	0.99
20	rs3787268	46013092	rs3918262	46015131	-2039	1	1
20	rs3787268	46013092	rs6073989	46022571	-9479	0.95	0.98
20	rs3787268	46013092	rs16991010	46023675	-10583	0.95	0.98
20	rs3787268	46013092	rs6130998	46024480	-11388	0.95	0.98
20	rs3787268	46013092	rs6130999	46026853	-13761	0.95	0.98
20	rs3787268	46013092	rs6073991	46027473	-14381	0.95	0.98
20	rs3787268	46013092	rs13039389	46031067	-17975	0.91	0.97

20	rs3787268	46013092	rs6131001	46032717	-19625	0.94	0.98
20	rs2250889	46013767	rs6094239	46024910	-11143	0.92	0.97
20	rs17577	46014472	rs1888235	45995328	19144	0.94	0.98
20	rs17577	46014472	rs6073984	46002014	12458	0.94	0.98
20	rs17577	46014472	rs6065912	46003728	10744	0.84	0.98
20	rs17577	46014472	rs3918241	46007096	7376	0.98	1

20	rs17577	46014472	rs3918242	46007337	7135	0.98	1
20	rs17577	46014472	rs2274755	46011053	3419	0.99	1
20	rs17577	46014472	rs2236416	46011936	2536	0.99	1
20	rs17577	46014472	rs3918261	46014953	-481	1	1
20	rs17577	46014472	rs13925	46016326	-1854	1	1
20	rs17577	46014472	rs3918270	46016700	-2228	0.98	1

20	rs17577	46014472	rs3918271	46016954	-2482	0.95	0.97
20	rs17577	46014472	rs66553311	46020003	-5531	0.84	1
20	rs17577	46014472	rs10432735	46021679	-7207	0.91	0.98

1MP genes on alternative splicing (cis-sQTL)

Gencode Id	Gene Symbol	Intron Id	p	Effect Size (β)	Tissue
ENSG0000	*WTAPP1*	10283290€	1.2E-34	-0.91	Testis
ENSG0000	*WTAPP1*	10283290€	7.7E-36	-0.89	Testis
ENSG0000	*WTAPP1*	10283290€	2.7E-37	-0.92	Testis
ENSG0000	*WTAPP1*	10283290€	2.7E-37	-0.92	Testis
ENSG0000	*WTAPP1*	10283290€	2.7E-37	-0.92	Testis
ENSG0000	*WTAPP1*	10283290€	5.3E-40	-0.95	Testis
ENSG0000	*WTAPP1*	10283290€	5E-39	-0.95	Testis
ENSG0000	*WTAPP1*	10283290€	1E-37	-0.93	Testis
ENSG0000	*WTAPP1*	10283290€	1.5E-40	-0.96	Testis
ENSG0000	*WTAPP1*	10283290€	6.3E-39	-0.94	Testis
ENSG0000	*WTAPP1*	10283290€	6.3E-39	-0.94	Testis
ENSG0000	*WTAPP1*	10283290€	2.5E-40	-0.96	Testis
ENSG0000	*WTAPP1*	10283290€	4.7E-40	-0.96	Testis
ENSG0000	*WTAPP1*	10283290€	1.1E-39	-0.96	Testis
ENSG0000	*WTAPP1*	10283290€	1.1E-39	-0.96	Testis
ENSG0000	*WTAPP1*	10283290€	1.2E-38	-0.93	Testis
ENSG0000	*WTAPP1*	10283290€	4.5E-41	-0.95	Testis
ENSG0000	*WTAPP1*	10283290€	5.3E-40	-0.93	Testis
ENSG0000	*WTAPP1*	10283290€	1.2E-38	-0.93	Testis
ENSG0000	*WTAPP1*	10283290€	9.3E-42	-0.96	Testis
ENSG0000	*WTAPP1*	10283290€	3.8E-42	-0.96	Testis
ENSG0000	*WTAPP1*	10283290€	3.8E-42	-0.96	Testis
ENSG0000	*WTAPP1*	10283290€	6.7E-43	0.96	Testis
ENSG0000	*WTAPP1*	10283290€	2E-41	0.95	Testis
ENSG0000	*WTAPP1*	10283290€	6.7E-43	0.96	Testis
ENSG0000	*WTAPP1*	10283290€	1.1E-40	0.94	Testis
ENSG0000	*WTAPP1*	10283290€	2E-41	0.95	Testis
ENSG0000	*WTAPP1*	10283290€	2E-41	0.95	Testis
ENSG0000	*WTAPP1*	10283290€	6.7E-43	0.96	Testis
ENSG0000	*WTAPP1*	10283290€	1.4E-41	0.95	Testis
ENSG0000	*WTAPP1*	10283290€	3.4E-43	0.97	Testis
ENSG0000	*WTAPP1*	10283290€	7.5E-41	0.94	Testis
ENSG0000	*WTAPP1*	10283290€	1.9E-41	0.95	Testis
ENSG0000	*WTAPP1*	10283290€	7.5E-41	0.94	Testis
ENSG0000	*WTAPP1*	10283290€	1.9E-41	0.95	Testis
ENSG0000	*WTAPP1*	10283290€	1.9E-41	0.95	Testis
ENSG0000	*CD40*	46126741:(	).00000000096	0.47	Lung
ENSG0000	*CD40*	46126741:(	).00000000096	0.47	Lung
ENSG0000	*CD40*	46126741:	0.0000000038	0.51	Thyroid
ENSG0000	*SLC12A5*	46021886:	0.000000018	0.78	Pituitary

ENSG0000	*CD40*	46126741:	0.00000018	0.51	Artery - Aorta
ENSG0000	*ACOT8*	45841956:	0.00000037	0.61	Heart - Left Ventricle
ENSG0000	*CD40*	46126741:	0.00000076	0.76	Cells - EBV-transformed lymphocytes
ENSG0000	*CD40*	46126741:	0.0000012	0.36	Adipose - Subcutaneous
ENSG0000	*CD40*	46126741:	0.0000017	0.41	Adipose - Visceral (Omentum)
ENSG0000	*CD40*	46126741:	0.0000017	0.41	Adipose - Visceral (Omentum)
ENSG0000	*CD40*	46126741:	0.0000035	0.39	Nerve - Tibial
ENSG0000	*CD40*	46126741:	).000000000096	0.47	Lung
ENSG0000	*CD40*	46126741:	).000000000096	0.47	Lung
ENSG0000	*CD40*	46126741:	0.0000000043	0.51	Thyroid
ENSG0000	*SLC12A5*	46021886:	0.000000018	0.78	Pituitary
ENSG0000	*CD40*	46126741:	0.00000018	0.51	Artery - Aorta
ENSG0000	*ACOT8*	45841956:	0.00000049	0.61	Heart - Left Ventricle
ENSG0000	*CD40*	46126741:	0.00000076	0.76	Cells - EBV-transformed lymphocytes
ENSG0000	*CD40*	46126741:	0.0000015	0.35	Adipose - Subcutaneous
ENSG0000	*CD40*	46126741:	0.0000017	0.41	Adipose - Visceral (Omentum)
ENSG0000	*CD40*	46126741:	0.0000017	0.41	Adipose - Visceral (Omentum)
ENSG0000	*CD40*	46126741:	0.0000035	0.39	Nerve - Tibial
ENSG0000	*CD40*	46126741:	).00000000028	0.52	Lung
ENSG0000	*CD40*	46126741:	).00000000028	0.52	Lung
ENSG0000	*CD40*	46126741:	0.0000000011	0.55	Thyroid
ENSG0000	*CD40*	46126741:	0.000000011	0.58	Artery - Aorta
ENSG0000	*ACOT8*	45841956:	0.000000024	0.69	Heart - Left Ventricle
ENSG0000	*SLC12A5*	46021886:	0.00000004	0.78	Pituitary
ENSG0000	*ACOT8*	45841956:	0.000000059	0.62	Heart - Atrial Appendage
ENSG0000	*CD40*	46126741:	0.000000062	0.88	Cells - EBV-transformed lymphocytes
ENSG0000	*CD40*	46126741:	0.00000046	0.45	Adipose - Visceral (Omentum)
ENSG0000	*CD40*	46126741:	0.00000046	0.45	Adipose - Visceral (Omentum)
ENSG0000	*CD40*	46126741:	0.00000062	0.38	Adipose - Subcutaneous
ENSG0000	*CD40*	46126741:	0.0000007	0.43	Nerve - Tibial
ENSG0000	*SLC12A5*	46023071:	0.0000045	-0.47	Lung
ENSG0000	*CD40*	46126741:	).000000000096	0.47	Lung
ENSG0000	*CD40*	46126741:	).000000000096	0.47	Lung
ENSG0000	*CD40*	46126741:	0.0000000043	0.51	Thyroid
ENSG0000	*SLC12A5*	46021886:	0.000000018	0.78	Pituitary
ENSG0000	*CD40*	46126741:	0.00000018	0.51	Artery - Aorta
ENSG0000	*ACOT8*	45841956:	0.00000049	0.61	Heart - Left Ventricle
ENSG0000	*CD40*	46126741:	0.00000076	0.76	Cells - EBV-transformed lymphocytes
ENSG0000	*CD40*	46126741:	0.0000015	0.35	Adipose - Subcutaneous
ENSG0000	*CD40*	46126741:	0.0000017	0.41	Adipose - Visceral (Omentum)
ENSG0000	*CD40*	46126741:	0.0000017	0.41	Adipose - Visceral (Omentum)
ENSG0000	*CD40*	46126741:	0.0000035	0.39	Nerve - Tibial
ENSG0000	*SLC12A5*	46021886:	).00000000026	0.86	Pituitary
ENSG0000	*CD40*	46126741:	0.0000000065	0.45	Lung
ENSG0000	*CD40*	46126741:	0.0000000065	0.45	Lung
ENSG0000	*CD40*	46126741:	0.000000015	0.47	Thyroid
ENSG0000	*CD40*	46126741:	0.0000002	0.5	Artery - Aorta

ENSG0000	*ACOT8*	45841956:	0.00000066	0.59	Heart - Left Ventricle
ENSG0000	*CD40*	46126741:	0.00000092	0.76	Cells - EBV-transformed lymphocytes
ENSG0000	*SLC12A5*	46023071:	0.0000012	-0.43	Brain - Cerebellar Hemisphere
ENSG0000	*ACOT8*	45841956:	0.0000025	0.49	Heart - Atrial Appendage
ENSG0000	*SLC12A5*	46021886:	0.0000000058	0.79	Pituitary
ENSG0000	*CD40*	46126741:	0.0000000067	0.45	Lung
ENSG0000	*CD40*	46126741:	0.0000000067	0.45	Lung
ENSG0000	*CD40*	46126741:	0.000000022	0.47	Thyroid
ENSG0000	*CD40*	46126741:	0.00000046	0.78	Cells - EBV-transformed lymphocytes
ENSG0000	*CD40*	46126741:	0.00000053	0.49	Artery - Aorta
ENSG0000	*ACOT8*	45841956:	0.0000015	0.58	Heart - Left Ventricle
ENSG0000	*ACOT8*	45841956:	0.0000039	0.49	Heart - Atrial Appendage
ENSG0000	*SLC12A5*	46023071:	0.000018	-0.42	Lung
ENSG0000	*SLC12A5*	46021886:	).0000000001	0.88	Pituitary
ENSG0000	*CD40*	46126741:	0.000000006	0.45	Lung
ENSG0000	*CD40*	46126741:	0.000000006	0.45	Lung
ENSG0000	*CD40*	46126741:	0.000000043	0.46	Thyroid
ENSG0000	*CD40*	46126741:	0.00000038	0.49	Artery - Aorta
ENSG0000	*CD40*	46126741:	0.00000092	0.76	Cells - EBV-transformed lymphocytes
ENSG0000	*SLC12A5*	46023071:	0.0000012	-0.43	Brain - Cerebellar Hemisphere
ENSG0000	*ACOT8*	45841956:	0.0000018	0.56	Heart - Left Ventricle
ENSG0000	*ACOT8*	45841956:	0.0000021	0.49	Heart - Atrial Appendage
ENSG0000	*SLC12A5*	46023071:	0.000017	-0.41	Lung
ENSG0000	*SLC12A5*	46021886:	).0000000001	0.88	Pituitary
ENSG0000	*CD40*	46126741:	0.000000027	0.42	Lung
ENSG0000	*CD40*	46126741:	0.000000027	0.42	Lung
ENSG0000	*CD40*	46126741:	0.000000067	0.45	Thyroid
ENSG0000	*CD40*	46126741:	0.00000055	0.47	Artery - Aorta
ENSG0000	*ACOT8*	45841956:	0.00000085	0.58	Heart - Left Ventricle
ENSG0000	*SLC12A5*	46023071:	0.0000012	-0.43	Brain - Cerebellar Hemisphere
ENSG0000	*CD40*	46126741:	0.0000023	0.73	Cells - EBV-transformed lymphocytes
ENSG0000	*ACOT8*	45841956:	0.0000036	0.48	Heart - Atrial Appendage
ENSG0000	*SLC12A5*	46021886:	0.000000002	0.82	Pituitary
ENSG0000	*CD40*	46126741:	0.000000011	0.44	Lung
ENSG0000	*CD40*	46126741:	0.000000011	0.44	Lung
ENSG0000	*CD40*	46126741:	0.000000012	0.48	Thyroid
ENSG0000	*CD40*	46126741:	0.0000004	0.49	Artery - Aorta
ENSG0000	*CD40*	46126741:	0.00000092	0.76	Cells - EBV-transformed lymphocytes
ENSG0000	*ACOT8*	45841956:	0.0000012	0.58	Heart - Left Ventricle
ENSG0000	*ACOT8*	45841956:	0.0000018	0.5	Heart - Atrial Appendage
ENSG0000	*SLC12A5*	46023071:	0.00002	-0.41	Lung
ENSG0000	*SLC12A5*	46021886:	).0000000002	0.86	Pituitary
ENSG0000	*CD40*	46126741:	0.000000018	0.43	Lung
ENSG0000	*CD40*	46126741:	0.000000018	0.43	Lung
ENSG0000	*CD40*	46126741:	0.000000032	0.46	Thyroid
ENSG0000	*CD40*	46126741:	0.00000032	0.48	Artery - Aorta
ENSG0000	*ACOT8*	45841956:	0.0000007	0.58	Heart - Left Ventricle

ENSG0000	*CD40*	46126741:	0.00000092	0.76	Cells - EBV-transformed lymphocytes
ENSG0000	*SLC12A5*	46023071:	0.0000012	-0.43	Brain - Cerebellar Hemisphere
ENSG0000	*ACOT8*	45841956:	0.0000021	0.49	Heart - Atrial Appendage
ENSG0000	*SLC12A5*	46021886:	).0000000002€	0.86	Pituitary
ENSG0000	*CD40*	46126741:	0.0000000065	0.45	Lung
ENSG0000	*CD40*	46126741:	0.0000000065	0.45	Lung
ENSG0000	*CD40*	46126741:	0.000000032	0.46	Thyroid
ENSG0000	*CD40*	46126741:	0.00000038	0.49	Artery - Aorta
ENSG0000	*ACOT8*	45841956:	0.0000007	0.58	Heart - Left Ventricle
ENSG0000	*CD40*	46126741:	0.00000092	0.76	Cells - EBV-transformed lymphocytes
ENSG0000	*SLC12A5*	46023071:	0.0000012	-0.43	Brain - Cerebellar Hemisphere
ENSG0000	*ACOT8*	45841956:	0.0000021	0.49	Heart - Atrial Appendage
ENSG0000	*SLC12A5*	46021886:	00000000000(	0.98	Pituitary
ENSG0000	*SLC12A5*	46021886:	00000000000(	0.99	Brain - Cerebellum
ENSG0000	*SLC12A5*	46021886:	)00000000000	0.74	Testis
ENSG0000	*SLC12A5*	46023071:	)00000000000	-0.57	Brain - Cerebellar Hemisphere
ENSG0000	*SLC12A5*	46023071:	0.0000000017	-0.69	Brain - Cortex
ENSG0000	*SLC12A5*	46021886:	0.00000097	0.43	Adipose - Visceral (Omentum)
ENSG0000	*CD40*	46126741:	0.0000013	0.36	Thyroid
ENSG0000	*SLC35C2*	46357702:	0.0000024	0.61	Brain - Cerebellum
ENSG0000	*ACOT8*	45841956:	0.000003	0.48	Heart - Left Ventricle
ENSG0000	*SLC12A5*	46021886:	).0000000002€	0.86	Pituitary
ENSG0000	*CD40*	46126741:	0.0000000065	0.45	Lung
ENSG0000	*CD40*	46126741:	0.0000000065	0.45	Lung
ENSG0000	*CD40*	46126741:	0.000000032	0.46	Thyroid
ENSG0000	*CD40*	46126741:	0.00000038	0.49	Artery - Aorta
ENSG0000	*ACOT8*	45841956:	0.0000007	0.58	Heart - Left Ventricle
ENSG0000	*CD40*	46126741:	0.00000092	0.76	Cells - EBV-transformed lymphocytes
ENSG0000	*SLC12A5*	46023071:	0.0000012	-0.43	Brain - Cerebellar Hemisphere
ENSG0000	*ACOT8*	45841956:	0.0000021	0.49	Heart - Atrial Appendage

ENSG0000	*SLC12A5*	46021886:	)00000000000	0.64	Pituitary
ENSG0000	*SLC12A5*	46021886:	0.0000000048	0.48	Testis
ENSG0000	*SLC12A5*	46021886:	0.000000039	0.5	Brain - Cerebellum
ENSG0000	*SLC12A5*	46023071:	0.000000041	-0.46	Brain - Cortex
ENSG0000	*SLC12A5*	46023071:	0.0000014	-0.3	Brain - Cerebellar Hemisphere
ENSG0000	*SLC12A5*	46021886:	0.0000000002	0.58	Pituitary
ENSG0000	*SLC12A5*	46023071:	0.0000013	-0.42	Brain - Cortex
ENSG0000	*SLC12A5*	46021886:	.00000000004	0.59	Pituitary
ENSG0000	*SLC12A5*	46021886:	0.000000075	0.45	Testis
ENSG0000	*SLC12A5*	46021886:	0.00000068	0.59	Brain - Substantia nigra
ENSG0000	*SLC12A5*	46023071:	0.00000079	-0.42	Brain - Cortex
ENSG0000	*SLC12A5*	46021886:	00000000000:	0.61	Pituitary
ENSG0000	*SLC12A5*	46021886:	0.000000052	0.46	Testis
ENSG0000	*SLC12A5*	46023071:	0.00000037	-0.43	Brain - Cortex
ENSG0000	*SLC12A5*	46021886:	0.00000051	0.59	Brain - Substantia nigra
ENSG0000	*SLC12A5*	46021886:	00000000000(	0.68	Pituitary

ENSG0000	*SLC12A5*	46021886:	.00000000001	0.6	Brain - Cerebellum
ENSG0000	*SLC12A5*	46021886:	.00000000003	0.53	Testis
ENSG0000	*SLC12A5*	46023071:	0.0000000012	-0.51	Brain - Cortex
ENSG0000	*SLC12A5*	46023071:	0.00000012	-0.32	Brain - Cerebellar Hemisphere
ENSG0000	*SLC12A5*	46021886:	.00000000002	0.58	Pituitary
ENSG0000	*SLC12A5*	46023071:	0.0000017	-0.41	Brain - Cortex
ENSG0000	*SNX21*	45835116:	0.0000069	-0.24	Muscle - Skeletal
ENSG0000	*SLC12A5*	46021886:	0.0000000001	0.59	Pituitary
ENSG0000	*SLC12A5*	46023071:	0.00000072	-0.43	Brain - Cortex
ENSG0000	*SLC12A5*	46021886:	0.0000000001	0.59	Pituitary
ENSG0000	*SLC12A5*	46023071:	0.00000072	-0.43	Brain - Cortex
ENSG0000	*SLC12A5*	46021886:	0.0000000093	0.54	Pituitary
ENSG0000	*ACOT8*	45841956:	0.00000011	0.41	Heart - Atrial Appendage
ENSG0000	*SNX21*	45835116:	0.0000036	-0.24	Muscle - Skeletal
ENSG0000	*PLTP*	45910070:	0.0000056	-0.23	Whole Blood
ENSG0000	*ACOT8*	45841956:	0.0000064	0.25	Nerve - Tibial
ENSG0000	*SLC12A5*	46021886:	00000000000	0.63	Pituitary
ENSG0000	*SLC12A5*	46023071:	0.00000033	-0.45	Brain - Cortex
ENSG0000	*SLC12A5*	46021886:	0.000002	0.46	Brain - Cerebellum
ENSG0000	*SLC12A5*	46021886:	0.000011	0.38	Testis
ENSG0000	*ACOT8*	45841956:	0.00000038	-0.41	Heart - Left Ventricle
ENSG0000	*SLC12A5*	46021886:	000000000000	0.64	Pituitary
ENSG0000	*SLC12A5*	46021886:	0.0000000048	0.48	Testis
ENSG0000	*SLC12A5*	46021886:	0.000000039	0.5	Brain - Cerebellum
ENSG0000	*SLC12A5*	46023071:	0.000000041	-0.46	Brain - Cortex
ENSG0000	*SLC12A5*	46023071:	0.0000014	-0.3	Brain - Cerebellar Hemisphere
ENSG0000	*SLC12A5*	46021886:	0.0000000002	0.58	Pituitary
ENSG0000	*SLC12A5*	46023071:	0.0000013	-0.42	Brain - Cortex
ENSG0000	*SLC12A5*	46021886:	.00000000004	0.59	Pituitary
ENSG0000	*SLC12A5*	46021886:	0.000000075	0.45	Testis
ENSG0000	*SLC12A5*	46021886:	0.00000068	0.59	Brain - Substantia nigra
ENSG0000	*SLC12A5*	46023071:	0.00000079	-0.42	Brain - Cortex
ENSG0000	*SLC12A5*	46021886:	00000000000	0.61	Pituitary
ENSG0000	*SLC12A5*	46021886:	0.000000052	0.46	Testis
ENSG0000	*SLC12A5*	46023071:	0.00000037	-0.43	Brain - Cortex
ENSG0000	*SLC12A5*	46021886:	0.00000051	0.59	Brain - Substantia nigra
ENSG0000	*SLC12A5*	46021886:	00000000000	0.68	Pituitary
ENSG0000	*SLC12A5*	46021886:	.00000000001	0.6	Brain - Cerebellum
ENSG0000	*SLC12A5*	46021886:	.00000000003	0.53	Testis
ENSG0000	*SLC12A5*	46023071:	0.0000000012	-0.51	Brain - Cortex
ENSG0000	*SLC12A5*	46023071:	0.00000012	-0.32	Brain - Cerebellar Hemisphere
ENSG0000	*SLC12A5*	46021886:	0.000055	0.31	Cells - Cultured fibroblasts
ENSG0000	*SLC12A5*	46021886:	.00000000002	0.58	Pituitary
ENSG0000	*SLC12A5*	46023071:	0.0000017	-0.41	Brain - Cortex
ENSG0000	*SNX21*	45835116:	0.0000069	-0.24	Muscle - Skeletal
ENSG0000	*SLC12A5*	46021886:	0.0000000001	0.59	Pituitary

ENSG0000	*SLC12A5*	46023071:	0.00000072	-0.43	Brain - Cortex
ENSG0000	*SLC12A5*	46021886:	0.0000000001	0.59	Pituitary
ENSG0000	*SLC12A5*	46023071:	0.00000072	-0.43	Brain - Cortex
ENSG0000	*SLC12A5*	46021886:	.00000000000	0.63	Pituitary
ENSG0000	*SLC12A5*	46021886:	0.0000000099	0.49	Testis
ENSG0000	*SLC12A5*	46023071:	0.00000029	-0.44	Brain - Cortex
ENSG0000	*SLC12A5*	46021886:	0.00000056	0.61	Brain - Substantia nigra
ENSG0000	*SLC12A5*	46023071:	0.00000078	-0.32	Brain - Cerebellar Hemisphere
ENSG0000	*SLC12A5*	46021886:	0.000002	0.45	Brain - Cerebellum
ENSG0000	*SLC12A5*	46021886:	0.0000062	0.45	Spleen
ENSG0000	*SLC12A5*	46021886:	0.0000000093	0.54	Pituitary
ENSG0000	*ACOT8*	45841956:	0.00000011	0.41	Heart - Atrial Appendage
ENSG0000	*SNX21*	45835116:	0.0000036	-0.24	Muscle - Skeletal
ENSG0000	*PLTP*	45910070:	0.0000056	-0.23	Whole Blood
ENSG0000	*ACOT8*	45841956:	0.0000064	0.25	Nerve - Tibial
ENSG0000	*ACOT8*	45841956:	0.00000038	-0.41	Heart - Left Ventricle
ENSG0000	*PLTP*	45907904:	0.00000011	0.37	Adipose - Subcutaneous
ENSG0000	*PLTP*	45907904:	0.0000012	0.41	Breast - Mammary Tissue
ENSG0000	*PLTP*	45907904:	0.0000025	0.33	Skin - Sun Exposed (Lower leg)
ENSG0000	*PLTP*	45907904:	0.000008	0.34	Lung
ENSG0000	*PLTP*	45907904:	0.00000013	0.36	Adipose - Subcutaneous
ENSG0000	*PLTP*	45907904:	0.0000022	0.39	Breast - Mammary Tissue
ENSG0000	*PLTP*	45907904:	0.0000025	0.33	Skin - Sun Exposed (Lower leg)
ENSG0000	*PLTP*	45907904:	0.000008	0.34	Lung
ENSG0000	*PLTP*	45907904:	0.00000028	0.35	Adipose - Subcutaneous
ENSG0000	*PLTP*	45907904:	0.0000025	0.39	Breast - Mammary Tissue
ENSG0000	*PLTP*	45907904:	0.0000056	0.32	Skin - Sun Exposed (Lower leg)
ENSG0000	*PLTP*	45907904:	0.00001	0.33	Lung
ENSG0000	*PLTP*	45907904:	0.00000021	0.36	Adipose - Subcutaneous
ENSG0000	*PLTP*	45907904:	0.0000011	0.41	Breast - Mammary Tissue
ENSG0000	*PLTP*	45907904:	0.0000074	0.32	Skin - Sun Exposed (Lower leg)
ENSG0000	*PLTP*	45907904:	0.000012	0.33	Lung
ENSG0000	*PLTP*	45907904:	0.00000015	0.36	Adipose - Subcutaneous
ENSG0000	*PLTP*	45907904:	0.00000056	0.42	Breast - Mammary Tissue
ENSG0000	*PLTP*	45907904:	0.000011	0.31	Skin - Sun Exposed (Lower leg)
ENSG0000	*PLTP*	45907904:	0.00000012	0.37	Adipose - Subcutaneous
ENSG0000	*PLTP*	45907904:	0.00000014	0.44	Breast - Mammary Tissue
ENSG0000	*PLTP*	45907904:	0.00000012	0.37	Adipose - Subcutaneous
ENSG0000	*PLTP*	45907904:	0.00000039	0.43	Breast - Mammary Tissue
ENSG0000	*PLTP*	45907904:	0.00000012	0.37	Adipose - Subcutaneous
ENSG0000	*PLTP*	45907904:	0.00000039	0.43	Breast - Mammary Tissue
ENSG0000	*PLTP*	45907904:	0.00000012	0.37	Adipose - Subcutaneous
ENSG0000	*PLTP*	45907904:	0.00000039	0.43	Breast - Mammary Tissue
ENSG0000	*PLTP*	45907904:	0.00000012	0.37	Adipose - Subcutaneous
ENSG0000	*PLTP*	45907904:	0.00000039	0.43	Breast - Mammary Tissue
ENSG0000	*PLTP*	45907904:	0.00000012	0.37	Adipose - Subcutaneous

ENSG0000	*PLTP*	45907904:	0.00000014	0.44	Breast - Mammary Tissue
ENSG0000	*PLTP*	45907904:	0.00000012	0.37	Adipose - Subcutaneous
ENSG0000	*PLTP*	45907904:	0.00000014	0.44	Breast - Mammary Tissue
ENSG0000	*SLC12A5*	46021886:	0.0000000061	44562	Pituitary
ENSG0000	*CD40*	46126741:	).000000000096	0.47	Lung
ENSG0000	*CD40*	46126741:	).000000000096	0.47	Lung
ENSG0000	*CD40*	46126741:	0.0000000038	0.51	Thyroid
ENSG0000	*SLC12A5*	46021886:	0.000000018	0.78	Pituitary
ENSG0000	*CD40*	46126741:	0.00000018	0.51	Artery - Aorta
ENSG0000	*ACOT8*	45841956:	0.00000037	0.61	Heart - Left Ventricle
ENSG0000	*CD40*	46126741:	0.00000076	0.76	Cells - EBV-transformed lymphocytes
ENSG0000	*CD40*	46126741:	0.0000012	0.36	Adipose - Subcutaneous
ENSG0000	*CD40*	46126741:	0.0000017	0.41	Adipose - Visceral (Omentum)
ENSG0000	*CD40*	46126741:	0.0000017	0.41	Adipose - Visceral (Omentum)
ENSG0000	*CD40*	46126741:	0.0000035	0.39	Nerve - Tibial
ENSG0000	*CD40*	46126741:	).000000000096	0.47	Lung
ENSG0000	*CD40*	46126741:	).000000000096	0.47	Lung
ENSG0000	*CD40*	46126741:	0.0000000043	0.51	Thyroid
ENSG0000	*SLC12A5*	46021886:	0.000000018	0.78	Pituitary
ENSG0000	*CD40*	46126741:	0.00000018	0.51	Artery - Aorta
ENSG0000	*ACOT8*	45841956:	0.00000049	0.61	Heart - Left Ventricle
ENSG0000	*CD40*	46126741:	0.00000076	0.76	Cells - EBV-transformed lymphocytes
ENSG0000	*CD40*	46126741:	0.0000015	0.35	Adipose - Subcutaneous
ENSG0000	*CD40*	46126741:	0.0000017	0.41	Adipose - Visceral (Omentum)
ENSG0000	*CD40*	46126741:	0.0000017	0.41	Adipose - Visceral (Omentum)
ENSG0000	*CD40*	46126741:	0.0000035	0.39	Nerve - Tibial
ENSG0000	*CD40*	46126741:	).000000000028	0.52	Lung
ENSG0000	*CD40*	46126741:	).000000000028	0.52	Lung
ENSG0000	*CD40*	46126741:	0.0000000011	0.55	Thyroid
ENSG0000	*CD40*	46126741:	0.000000011	0.58	Artery - Aorta
ENSG0000	*ACOT8*	45841956:	0.000000024	0.69	Heart - Left Ventricle
ENSG0000	*SLC12A5*	46021886:	0.00000004	0.78	Pituitary
ENSG0000	*ACOT8*	45841956:	0.000000059	0.62	Heart - Atrial Appendage
ENSG0000	*CD40*	46126741:	0.000000062	0.88	Cells - EBV-transformed lymphocytes
ENSG0000	*CD40*	46126741:	0.00000046	0.45	Adipose - Visceral (Omentum)
ENSG0000	*CD40*	46126741:	0.00000046	0.45	Adipose - Visceral (Omentum)
ENSG0000	*CD40*	46126741:	0.00000062	0.38	Adipose - Subcutaneous
ENSG0000	*CD40*	46126741:	0.0000007	0.43	Nerve - Tibial
ENSG0000	*SLC12A5*	46023071:	0.0000045	-0.47	Lung
ENSG0000	*CD40*	46126741:	0.0000000022	0.51	Thyroid
ENSG0000	*CD40*	46126741:	0.0000000092	0.44	Lung
ENSG0000	*CD40*	46126741:	0.0000000092	0.44	Lung
ENSG0000	*SLC12A5*	46021886:	0.0000000098	0.79	Pituitary
ENSG0000	*CD40*	46126741:	0.00000049	0.48	Artery - Aorta
ENSG0000	*ACOT8*	45841956:	0.0000011	0.58	Heart - Left Ventricle

ENSG0000	*CD40*	46126741:	0.0000015	0.74	Cells - EBV-transformed lymphocytes
ENSG0000	*ACOT8*	45841956:	0.0000033	0.5	Heart - Atrial Appendage
ENSG0000	*CD40*	46126741:	0.0000000029	0.5	Thyroid
ENSG0000	*CD40*	46126741:	0.0000000067	0.45	Lung
ENSG0000	*CD40*	46126741:	0.0000000067	0.45	Lung
ENSG0000	*SLC12A5*	46021886:	0.0000000098	0.79	Pituitary
ENSG0000	*CD40*	46126741:	0.00000041	0.49	Artery - Aorta
ENSG0000	*ACOT8*	45841956:	0.0000011	0.58	Heart - Left Ventricle
ENSG0000	*CD40*	46126741:	0.0000015	0.74	Cells - EBV-transformed lymphocytes
ENSG0000	*ACOT8*	45841956:	0.0000041	0.49	Heart - Atrial Appendage
ENSG0000	*SLC12A5*	46021886:	).000000000026	0.86	Pituitary
ENSG0000	*CD40*	46126741:	0.0000000065	0.45	Lung
ENSG0000	*CD40*	46126741:	0.0000000065	0.45	Lung
ENSG0000	*CD40*	46126741:	0.000000015	0.47	Thyroid
ENSG0000	*CD40*	46126741:	0.0000002	0.5	Artery - Aorta
ENSG0000	*ACOT8*	45841956:	0.00000066	0.59	Heart - Left Ventricle
ENSG0000	*CD40*	46126741:	0.00000092	0.76	Cells - EBV-transformed lymphocytes
ENSG0000	*SLC12A5*	46023071:	0.0000012	-0.43	Brain - Cerebellar Hemisphere
ENSG0000	*ACOT8*	45841956:	0.0000025	0.49	Heart - Atrial Appendage
ENSG0000	*SLC12A5*	46021886:	0.0000000058	0.79	Pituitary
ENSG0000	*CD40*	46126741:	0.0000000067	0.45	Lung
ENSG0000	*CD40*	46126741:	0.0000000067	0.45	Lung
ENSG0000	*CD40*	46126741:	0.000000022	0.47	Thyroid
ENSG0000	*CD40*	46126741:	0.00000046	0.78	Cells - EBV-transformed lymphocytes
ENSG0000	*CD40*	46126741:	0.00000053	0.49	Artery - Aorta
ENSG0000	*ACOT8*	45841956:	0.0000015	0.58	Heart - Left Ventricle
ENSG0000	*ACOT8*	45841956:	0.0000039	0.49	Heart - Atrial Appendage
ENSG0000	*SLC12A5*	46023071:	0.000018	-0.42	Lung
ENSG0000	*SLC12A5*	46021886:	).000000000011	0.88	Pituitary
ENSG0000	*CD40*	46126741:	0.000000027	0.42	Lung
ENSG0000	*CD40*	46126741:	0.000000027	0.42	Lung
ENSG0000	*CD40*	46126741:	0.000000067	0.45	Thyroid
ENSG0000	*CD40*	46126741:	0.00000055	0.47	Artery - Aorta
ENSG0000	*ACOT8*	45841956:	0.00000085	0.58	Heart - Left Ventricle
ENSG0000	*SLC12A5*	46023071:	0.0000012	-0.43	Brain - Cerebellar Hemisphere
ENSG0000	*CD40*	46126741:	0.0000023	0.73	Cells - EBV-transformed lymphocytes
ENSG0000	*ACOT8*	45841956:	0.0000036	0.48	Heart - Atrial Appendage
ENSG0000	*SLC12A5*	46021886:	0.000000002	0.82	Pituitary
ENSG0000	*CD40*	46126741:	0.000000011	0.44	Lung
ENSG0000	*CD40*	46126741:	0.000000011	0.44	Lung
ENSG0000	*CD40*	46126741:	0.000000012	0.48	Thyroid
ENSG0000	*CD40*	46126741:	0.0000004	0.49	Artery - Aorta
ENSG0000	*CD40*	46126741:	0.00000092	0.76	Cells - EBV-transformed lymphocytes
ENSG0000	*ACOT8*	45841956:	0.0000012	0.58	Heart - Left Ventricle
ENSG0000	*ACOT8*	45841956:	0.0000018	0.5	Heart - Atrial Appendage
ENSG0000	*SLC12A5*	46023071:	0.00002	-0.41	Lung
ENSG0000	*SLC12A5*	46021886:	).000000000026	0.86	Pituitary

ENSG0000	*CD40*	46126741:	0.000000018	0.43	Lung
ENSG0000	*CD40*	46126741:	0.000000018	0.43	Lung
ENSG0000	*CD40*	46126741:	0.000000032	0.46	Thyroid
ENSG0000	*CD40*	46126741:	0.00000032	0.48	Artery - Aorta
ENSG0000	*ACOT8*	45841956:	0.0000007	0.58	Heart - Left Ventricle
ENSG0000	*CD40*	46126741:	0.00000092	0.76	Cells - EBV-transformed lymphocytes
ENSG0000	*SLC12A5*	46023071:	0.0000012	-0.43	Brain - Cerebellar Hemisphere
ENSG0000	*ACOT8*	45841956:	0.0000021	0.49	Heart - Atrial Appendage
ENSG0000	*SLC12A5*	46021886:	).0000000002(	0.86	Pituitary
ENSG0000	*CD40*	46126741:	0.0000000065	0.45	Lung
ENSG0000	*CD40*	46126741:	0.0000000065	0.45	Lung
ENSG0000	*CD40*	46126741:	0.000000032	0.46	Thyroid
ENSG0000	*CD40*	46126741:	0.00000038	0.49	Artery - Aorta
ENSG0000	*ACOT8*	45841956:	0.0000007	0.58	Heart - Left Ventricle
ENSG0000	*CD40*	46126741:	0.00000092	0.76	Cells - EBV-transformed lymphocytes
ENSG0000	*SLC12A5*	46023071:	0.0000012	-0.43	Brain - Cerebellar Hemisphere
ENSG0000	*ACOT8*	45841956:	0.0000021	0.49	Heart - Atrial Appendage
ENSG0000	*SLC12A5*	46021886:	00000000000(	0.98	Pituitary
ENSG0000	*SLC12A5*	46021886:	00000000000(	0.99	Brain - Cerebellum
ENSG0000	*SLC12A5*	46021886:	)00000000000	0.74	Testis
ENSG0000	*SLC12A5*	46023071:	)00000000000	-0.57	Brain - Cerebellar Hemisphere
ENSG0000	*SLC12A5*	46023071:	0.0000000017	-0.69	Brain - Cortex
ENSG0000	*SLC12A5*	46021886:	0.00000097	0.43	Adipose - Visceral (Omentum)
ENSG0000	*CD40*	46126741:	0.0000013	0.36	Thyroid
ENSG0000	*SLC35C2*	46357702:	0.0000024	0.61	Brain - Cerebellum
ENSG0000	*ACOT8*	45841956:	0.000003	0.48	Heart - Left Ventricle
ENSG0000	*SLC12A5*	46021886:	).0000000002(	0.86	Pituitary
ENSG0000	*CD40*	46126741:	0.0000000065	0.45	Lung
ENSG0000	*CD40*	46126741:	0.0000000065	0.45	Lung
ENSG0000	*CD40*	46126741:	0.000000032	0.46	Thyroid
ENSG0000	*CD40*	46126741:	0.00000038	0.49	Artery - Aorta
ENSG0000	*ACOT8*	45841956:	0.0000007	0.58	Heart - Left Ventricle
ENSG0000	*CD40*	46126741:	0.00000092	0.76	Cells - EBV-transformed lymphocytes
ENSG0000	*SLC12A5*	46023071:	0.0000012	-0.43	Brain - Cerebellar Hemisphere
ENSG0000	*ACOT8*	45841956:	0.0000021	0.49	Heart - Atrial Appendage
