# Supplementary material for: Matrix Metalloproteinase Gene Polymorphisms Are Associated with Breast Cancer in the Caucasian Women of Russia
Source: Int J Mol Sci. 2022 Oct 20;23(20):12638. doi: 10.3390/ijms232012638 (PMC9604098; doi:10.3390/ijms232012638)
Supplement: Supplementary file 1 [file ijms-23-12638-s001.zip › Suppl Table s15.pdf]

Supplementary table S15

The literature data about associations of the studied polymorphisms of the *MMP* genes with breast cancer

Chr	SNP	Gene	Number of publications in PubMed/PubMed Central	Phenotype	Association, significance (associated allele/genotype/ haplotype)	Reference
11	rs1940475	*MMP-8*	9/25	breast cancer	p>0.05	Pharoah et al., 2007
				breast cancer metastasis	OR=0.60, p=0.03 (T)	Decock et al., 2007
				breast cancer	p>0.05	Mavaddat et al., 2009
				breast cancer	p>0.05	Wang et al., 2018
				breast cancer	OR=1.23, p=0.048 (haplotype AGTCA rs3740938-rs2012390-rs1940475-rs11225394-rs11225395)	Wang et al., 2018
11	rs1799750	*MMP-1*	92/156	breast cancer	p>0.05	Biondi et al., 2000
				breast cancer metastasis	p>0.05	Ghilardi et al., 2002
				breast cancer metastasis	OR>1, p<0.001 (2G)	Przybylowska et al., 2004
				breast cancer metastasis	OR=2.14, p<0.05 (2G/2G)<br>OR=1.68, p<0.05 (2G)	Przybylowska et al., 2006
				breast cancer malignance	OR=2.58, p<0.05 (2G)	Przybylowska et al., 2006
				lymph node-positive breast cancer	OR=3.9, p<0.05 (2G/2G) (mixed ethnicities)<br>OR=2.6, p<0.05 (2G/2G) (caucasian)	Hughes et al., 2007
				reduced survival among breast cancer women	HR=3.1, p<0.05 (2G/2G)	Hughes et al., 2007
				breast cancer	p>0.05	Beeghly-Fadiel et al., 2009a
				breast cancer	p>0.05	McColgan et al., 2009
				breast cancer	p>0.05	Zhou et al., 2011
				breast cancer metastasis	OR=2.18, p<0.05 (2G/2G)<br>OR=1.59, p<0.05 (2G/2G + 1G/2G)	Liu et al., 2012

				breast cancer	p>0.05	Su et al., 2016
				breast cancer	p>0.05	Zhou et al., 2018
				breast cancer	OR>1, p<0.05 (2G)	Padala et al., 2017
				breast cancer	p>0.05	Hsiao et al., 2018
				breast cancer	p>0.05	Białkowska et al., 2020
				breast cancer	OR = 4.86, p = 0.0001 (2G2G *vs.* 1G1G)	Balkhi et al., 2020
				breast cancer	OR<1, p<0.05 (1G/2G)	Sui et al., 2021
11	rs679620	*MMP-3*	41/100	breast cancer	p>0.05	Beeghly-Fadiel et al., 2009a
				breast cancer (lymph node or distant metastasis)	OR<1, p=0.049 (AG)	Chan, 2013
16	rs243865	*MMP-2*	92/109	breast cancer	OR=0.46, p<0.05 (CT+ TT *vs.* CC)	Zhou et al., 2004
				poor survival among patients with ER negative of breast cancer	p < 0.001 (TT *vs.* CC +CT)	Grieu et al., 2004
				breast cancer	p>0.05	Lei et al., 2007
				breast cancer progesterone receptor status	OR 2.34, p<0.05 (combination TT rs243865 and CC rs3918242)	Lei et al., 2007
				breast cancer	p>0.05	Roehe et al., 2007
				breast cancer	OR=2.15, p=0.01 (CC)	Delgado-Enciso et al., 2008
				breast cancer among women younger than 50 years of age	OR=2.66, p<0.05 (CC)	Delgado-Enciso et al., 2008
				breast cancer	p>0.05	Beeghly-Fadiel et al., 2009b
				breast cancer	p>0.05	McColgan et al., 2009
				breast cancer	OR = 1.27, p = 0.001 (CC)	Zhou et al., 2011
				breast cancer metastasis	p>0.05	Liu et al., 2012
				breast cancer	OR=2.02, p=0.02 (CC)	Saeed et al., 2013
				breast cancer	p>0.05	Zagouri et al., 2013
				breast cancer	p>0.05	Slattery et al., 2013

				breast cancer	p>0.05	Ledwoń et al., 2013
				breast cancer	p>0.05	Shevchenko et al., 2014
				breast cancer (summary, European and Asian)	p>0.05	Yang et al., 2014
				breast cancer (Latin-Americans)	OR=0.61, p=0.02 (TT+TC vs CC)	Yang et al., 2014
				breast cancer	OR=0.49, p<0.05 (TT+TC vs CC)	Néjima et al., 2015
				breast cancer	p>0.05	Manshadi et al., 2018
				breast cancer	OR=0.001, p<0.05	Habel et al., 2019
				Histological type (ductal/lobular/mixed) of breast cancer	P=0.002	Habel et al., 2019
				breast cancer	OR=0.39-0.49, p<0.0025 (haplotypes GCTT and GTTC rs243866-rs243865- rs243864- rs2285053)	Habel et al., 2019
				breast cancer	p>0.05	Ou et al., 2020
				breast cancer	p>0.05	Białkowska et al., 2020
20	rs3918242	*MMP-9*	139/194	good prognosis (non-ductal type histology, positive ER status and the absence of TP53 mutation) among breast cancer patients	p < 0.05 (T)	Grieu et al., 2004
				breast cancer metastasis	p>0.05	Przybylowska et al., 2006
				breast cancer malignance	OR=2.61, p<0.05 (T)	Przybylowska et al., 2006
				breast cancer	p>0.05	Roehe et al., 2007
				breast cancer	p>0.05	Lei et al., 2007
				breast cancer progesterone receptor status	OR 2.34, p<0.05 (combination TT rs243865 and CC rs3918242)	Lei et al., 2007
				lymph node-positive	OR=3.6, p<0.05 (TT) (mixed	Hughes et al., 2007

				breast cancer	ethnicities)<br>OR=9.1, p<0.05 (TT) (caucasian)	
				breast cancer	OR=3.27, p=0.004 (T)	Sadeghi et al., 2009
				breast cancer	p>0.05	McColgan et al., 2009
				breast cancer	p>0.05	Beeghly-Fadiel et al., 2011
				breast cancer	p>0.05	Zhou et al., 2011
				breast cancer metastasis	p>0.05	Liu et al., 2012
				breast cancer	OR>1, p<0.001 (T)	Chiranjeevi et al., 2014
				breast cancer	OR=0.22, p=0.007 (TT)	Shevchenko et al., 2014
				breast cancer lymph node metastasis	OR=0.60, p=0.45 (CC)	Shevchenko et al., 2014
				breast cancer	OR=1.55, p=0.009 (TT *vs.* CT+CC)	Zhang et al., 2015
				breast cancer	OR=1.87, p=0.035(T)	Rahimi et al., 2015
				breast cancer	OR=1.8, p<0.05 (T)	AbdRaboh et al., 2016
				breast cancer	p>0.05	Bargostavan et al., 2016
				breast cancer	p>0.05	Toroghi et al., 2017
				breast cancer	OR>1, p<0.05 (T)	Padala et al., 2017
				breast cancer	OR=4.82, p<0.001 (T)	Manshadi et al., 2018
				breast cancer	p>0.05	Felizi et al., 2018
				breast cancer	p>0.05	Abd Elmaogoud Ragab Ibrahim et al., 2020
				breast cancer	OR=1.34, p=0.028 (T)	Xu et al., 2020
				breast cancer	OR=1.43, p=0.002 (T)	Yan et al., 2022
20	rs3918249	*MMP-9*	16/25	breast cancer	p>0.05	Beeghly-Fadiel et al., 2011
				breast cancer	p>0.05	Slattery et al., 2013
				ER-/PR- status of breast cancer	OR=1.18, p=0.048 and OR=0.73, p=0.016 (haplotype AT and GC rs3918261-rs3918249 respectively)	Slattery et al., 2013
				ER+/PR- status of breast cancer	OR=0.71, p=0.044 and OR=5.02, p=0.02 (haplotype GC and GT rs3918261-rs3918249 respectively)	Slattery et al., 2013

				breast cancer	p>0.05	Wang et al., 2018
				breast cancer	OR=1.37, p=0.019 (haplotype CCG rs3918249-rs3918254-rs3787268)	Wang et al., 2018
20	rs17576	*MMP-9*	94/127	breast cancer	p>0.05	Beeghly-Fadiel et al., 2011
				breast cancer	OR=1.21, p=0.03 (Q279R)	Resler et al., 2013
				breast cancer survival	p>0.05	Fu et al., 2013
				breast cancer	OR=4.73, p=0.006 (GG)	Chahil et al., 2015
				breast cancer	p>0.05	Zhang et al., 2015
				breast cancer	OR=13.13, p<0.003 (GG)	Oliveira et al., 2020
				breast cancer in premenopausal women	p>0.05	Oliveira et al., 2020
				breast cancer in postmenopausal women	OR>1, p<0.006 (GG)	Oliveira et al., 2020
				breast cancer	p>0.05	Xu et al., 2020
				breast cancer	p>0.05	Yan et al., 2022
20	rs3787268	*MMP-9*	20/33	breast cancer	p>0.05	Beeghly-Fadiel et al., 2011
				poor disease-free survival of breast cancer patients	p=0.045 (AA+GA)	Fu et al., 2013
				poor distance disease-free survival of breast cancer patients	p=0.028 (AA+GA)	Fu et al., 2013
				breast cancer	p>0.05	Slattery et al., 2013
				breast cancer (among women with the 71–100% Native American Ancestry)	OR=1.52, p=0.037 (AA+GA *vs.* GG)	Slattery et al., 2013
				breast cancer	p>0.05	Zhang et al., 2015
				breast cancer	OR=0.82, p=0.025 (A)	Wang et al., 2018
				breast cancer	OR=1.37, p=0.019 (haplotype CCG rs3918249-rs3918254-rs3787268)	Wang et al., 2018
				breast cancer	p>0.05	Xu et al., 2020

				breast cancer	OR=0.82, p=0.007 (A)	Yan et al., 2022
20	rs2250889	*MMP-9*	22/38	breast cancer	p>0.05	Beeghly-Fadiel et al., 2011
				breast cancer survival	p>0.05	Fu et al., 2013
				breast cancer	OR=10.84, p=0.007 (GG)	Chahil et al., 2015
				breast cancer	p>0.05	Zhang et al., 2015
				breast cancer	p>0.05	Al-Eitan et al., 2017
				breast cancer	OR=2.53, p=0.012 (GG+GC vs. CC)	Xu et al., 2020
				breast cancer	p>0.05	Yan et al., 2022
20	rs17577	*MMP-9*	28/30	poor disease-free survival among estrogen receptor (ER)+/epidermal growth receptor 2 (HER-2)−breast cancer patients	HR=2.59, p<0.05 (combination AA+GA rs3787268 and GG rs17577)	Fu et al., 2013
				poor distance disease-free survival among estrogen receptor (ER)+/epidermal growth receptor 2 (HER-2)−breast cancer patients	HR=3.25, p<0.05 (combination AA+GA rs3787268 and GG rs17577)	Fu et al., 2013

References

1. Abd Elmaogoud Ragab Ibrahim F, Essam Elfeky S, Haroun M, et al. Association of matrix metalloproteinases 3 and 9 single nucleotide polymorphisms with breast cancer risk: A case-control study. *Mol Clin Oncol*. 2020;13(1):54-62. doi:10.3892/mco.2020.2041
2. AbdRaboh, N.R., Bayoumi, F.A., 2016. Gene polymorphism of matrix metalloproteinases 3 and 9 in breast cancer. Gene Reports 5, 151–156.
3. Al-Eitan LN, Jamous RI, Khasawneh RH. Candidate Gene Analysis of Breast Cancer in the Jordanian Population of Arab Descent: A Case-Control Study. *Cancer Invest*. 2017;35(4):256-270. doi:10.1080/07357907.2017.1289217
4. Balkhi S, Mashayekhi F, Salehzadeh A, Saedi HS. Matrix metalloproteinase (MMP)-1 and MMP-3 gene variations affect MMP-1 and -3 serum concentration and associates with breast cancer. Mol Biol Rep. 2020 Dec;47(12):9637-9644. doi: 10.1007/s11033-020-05962-x.
5. Bargostavan, M.H., Eslami, G., Esfandiari, N., Shams, S.A., 2016. MMP9 Promoter Polymorphism (-1562 C/T) Does not Affect the Serum Levels of Soluble MICB and MICA in Breast Cancer. Iran. J. Immunol. 13 (1), 45–53.
6. Beeghly-Fadiel A, Cai Q, Lu W, Long J, Gao YT, Shu XO, et al. No association between matrix metalloproteinase-1 or matrix metalloproteinase-3 polymorphisms and breast cancer susceptibility: a report from the Shanghai Breast Cancer Study. Cancer Epidemiol Biomarkers Prev. 2009a;18:1324–1327.
7. Beeghly-Fadiel A, Lu W, Long JR, Shu XO, Zheng Y, et al. (2009b) Matrix metalloproteinase-2 polymorphisms and breast cancer susceptibility. *Cancer epidemiology, biomarkers & prevention : a publication of the American Association for Cancer Research, cosponsored by the American Society of Preventive Oncology* 18: 1770–1776.
8. Beeghly-Fadiel A, Lu W, Shu XO, Long J, Cai Q, Xiang Y, Gao YT, Zheng W. MMP9 polymorphisms and breast cancer risk: a report from the Shanghai Breast Cancer Genetics Study. Breast Cancer Res Treat. 2011 Apr;126(2):507-13. doi: 10.1007/s10549-010-1119-1.
9. Białkowska K, Marciniak W, Muszyńska M, Baszuk P, Gupta S, Jaworska-Bieniek K, Sukiennicki G, Durda K, Gromowski T, Lener M, Prajzendanc K, Łukomska A, Cybulski C, Huzarski T, Gronwald J, Dębniak T, Lubiński J, Jakubowska A. Polymorphisms in *MMP-1*, *MMP-2*, *MMP-7, MMP-13* and *MT2A* do not contribute to breast, lung and colon cancer risk in polish population. Hered Cancer Clin Pract. 2020;18:16. doi: 10.1186/s13053-020-00147-w.
10. Biondi ML, Turri O, Leviti S, Seminati R, Cecchini F, Bernini M, Ghilardi G, Guagnellini E. MMP1 and MMP3 polymorphisms in promoter regions and cancer. Clin Chem. 2000;46(12):2023-4.
11. Chahil JK, Munretnam K, Samsudin N, Lye SH, Hashim NA, Ramzi NH, Velapasamy S, Wee LL, Alex L. Genetic polymorphisms associated with breast cancer in malaysian cohort. Indian J Clin Biochem. 2015 Apr;30(2):134-9. doi: 10.1007/s12291-013-0414-0.
12. Chan SC. Identification and analysis of single nucleotide polymorphisms in matrix metallopeptidase 2 and 3 genes in Malaysian breast cancer patients [doctor's thesis] Serdang: Universiti Putra Malaysia; 2013.
13. Chiranjeevi P, Spurthi KM, Rani NS, Kumar GR, Aiyengar TM, Saraswati M, Srilatha G, Kumar GK, Sinha S, Kumari CS, Reddy BN, Vishnupriya S, Rani HS. Gelatinase B (-1562C/T) polymorphism in tumor progression and invasion of breast cancer. Tumour Biol. 2014 Feb;35(2):1351-6. doi: 10.1007/s13277-013-1181-5.

14. Decock J., Long J.R., Laxton R.C., Shu X.O., Hodgkinson C., Hendrickx W., Pearce E.G., Gao Y.T., Pereira A.C., Paridaens R., et al. Association of matrix metalloproteinase-8 gene variation with breast cancer prognosis. *Cancer Res.* 2007;67:10214–10221. doi: 10.1158/0008-5472.CAN-07-1683.

15. Delgado-Enciso I, Cepeda-Lopez FR, Monrroy-Guizar EA, Bautista-Lam JR, Andrade-Soto M, et al. (2008) Matrix metalloproteinase-2 promoter polymorphism is associated with breast cancer in a Mexican population. *Gynecologic and obstetric investigation* 65: 68–72.

16. Felizi RT, Veiga MG, Carelli Filho I, Souto RPD, Fernandes CE, Oliveira E. Association between Matrix Metallopeptidase 9 Polymorphism and Breast Cancer Risk. Rev Bras Ginecol Obstet. 2018 Oct;40(10):620-624. English. doi: 10.1055/s-0038-1673366.

17. Fu F, Wang C, Chen LM, Huang M, Huang HG. The influence of functional polymorphisms in matrix metalloproteinase 9 on survival of breast cancer patients in a Chinese population. DNA Cell Biol. 2013 May;32(5):274-82. doi: 10.1089/dna.2012.1928.

18. Ghilardi G, Biondi ML, Caputo M, Leviti S, DeMonti M, Guagnellini E, Scorza R. A single nucleotide polymorphism in the matrix metalloproteinase-3 promoter enhances breast cancer susceptibility. Clin Cancer Res. 2002;8(12):3820-3.

19. Grieu F, Li WQ, Iacopetta B (2004) Genetic polymorphisms in the MMP-2 and MMP-9 genes and breast cancer phenotype. *Breast Cancer Research and Treatment* 88: 197–204.

20. Habel AF, Ghali RM, Bouaziz H, Daldoul A, Hadj-Ahmed M, Mokrani A, Zaied S, Hechiche M, Rahal K, Yacoubi-Loueslati B, Almawi WY. Common matrix metalloproteinase-2 gene variants and altered susceptibility to breast cancer and associated features in Tunisian women. Tumour Biol. 2019 Apr;41(4):1010428319845749. doi: 10.1177/1010428319845749.

21. Hsiao CL, Liu LC, Shih TC, Lai YL, Hsu SW, Wang HC, Pan SY, Shen TC, Tsai CW, Chang WS, Su CH, Way TD, Chung JG, Bau DT. The Association of *Matrix Metalloproteinase-1* Promoter Polymorphisms with Breast Cancer. In Vivo. 2018;32(3):487-491. doi: 10.21873/invivo.11265.

22. Hughes S, Agbaje O, Bowen RL, Holliday DL, Shaw JA, Duffy S, Jones JL. Matrix metalloproteinase single-nucleotide polymorphisms and haplotypes predict breast cancer progression. Clin Cancer Res. 2007;13(22 Pt 1):6673-80. doi: 10.1158/1078-0432.CCR-07-0884.

23. Ledwoń JK, Hennig EE, Maryan N, Goryca K, Nowakowska D, Niwińska A, Ostrowski J. Common low-penetrance risk variants associated with breast cancer in Polish women. BMC Cancer. 2013 Oct 30;13:510. doi: 10.1186/1471-2407-13-510.

24. Lei H, Hemminki K, Altieri A, Johansson R, Enquist K, Hallmans G, Lenner P, Försti A. Promoter polymorphisms in matrix metalloproteinases and their inhibitors: few associations with breast cancer susceptibility and progression. Breast Cancer Res Treat. 2007;103(1):61-9. doi: 10.1007/s10549-006-9345-2.

25. Liu D, Guo H, Li Y, Xu X, Yang K, et al. (2012) Association between polymorphisms in the promoter regions of matrix metalloproteinases (MMPs) and risk of cancer metastasis: a meta-analysis. *PLoS One* 7: e31251.

26. Manshadi, Z.D., Hamid, M., Kosari, F., Tayebinia, H., Khodadadi, I., 2018. The relationship between matrix metalloproteinase gene polymorphisms and tumor type, tumor size, and metastasis in women with breast cancer in Central Iran. Middle East J. Cancer 9 (2), 123–131.

27. Mavaddat N, Dunning AM, Ponder BA, Easton DF, Pharoah PD. Common genetic variation in candidate genes and susceptibility to subtypes of breast cancer. *Cancer Epidemiol Biomarkers Prev*. 2009;18(1):255-259. doi:10.1158/1055-9965.EPI-08-0704

28. McColgan P, Sharma P. Polymorphisms of matrix metalloproteinases 1, 2, 3 and 9 and susceptibility to lung, breast and colorectal cancer in over 30,000 subjects. Int J Cancer. 2009 Sep 15;125(6):1473-8. doi: 10.1002/ijc.24441.

29. Néjima DB, Zarkouna YB, Gammoudi A, Manai M, Boussen H. Prognostic impact of polymorphism of matrix metalloproteinase-2 and metalloproteinase tissue inhibitor-2 promoters in breast cancer in tunisia: Case-control study. Tumour Biol 36(5): 3815-3822, 2015. DOI: 10.1007/s13277-014-3023-5

30. Oliveira VA, Chagas DC, Amorim JR, Pereira RO, Nogueira TA, Borges VML, Campos-Verde LM, Martins LM, Rodrigues GP, Nery Júnior EJ, Sampaio FA, Lopes-Costa PV, Sousa JMCE, Silva VC, Silva FCCD, Silva BBD. Association between matrix metalloproteinase-9 gene polymorphism and breast cancer in Brazilian women. Clinics (Sao Paulo). 2020 Oct 26;75:e1762. doi: 10.6061/clinics/2020/e1762.

31. Ou YX, Bi R. Meta-analysis on the relationship between the SNP of MMP-2-1306 C>T and susceptibility to breast cancer. Eur Rev Med Pharmacol Sci. 2020 Feb;24(3):1264-1270. doi: 10.26355/eurrev_202002_20181.

32. Padala C, Tupurani MA, Puranam K, et al. Synergistic effect of collagenase-1 (MMP1), stromelysin-1 (MMP3) and gelatinase-B (MMP9) gene polymorphisms in breast cancer. *PLoS One*. 2017;12(9):e0184448. doi:10.1371/journal.pone.0184448

33. Pharoah PD, Tyrer J, Dunning AM, Easton DF, Ponder BA; SEARCH Investigators. Association between common variation in 120 candidate genes and breast cancer risk. *PLoS Genet*. 2007;3(3):e42. doi:10.1371/journal.pgen.0030042

34. Przybylowska K, Kluczna A, Zadrozny M, Krawczyk T, Kulig A, Rykala J, Kolacinska A, Morawiec Z, Drzewoski J, Blasiak J. Polymorphisms of the promoter regions of matrix metalloproteinases genes MMP-1 and MMP-9 in breast cancer. Breast Cancer Res Treat. 2006;95(1):65-72. doi: 10.1007/s10549-005-9042-6.

35. Przybylowska K, Zielinska J, Zadrozny M, Krawczyk T, Kulig A, Wozniak P, Rykala J, Kolacinska A, Morawiec Z, Drzewoski J, Blasiak J. An association between the matrix metalloproteinase 1 promoter gene polymorphism and lymphnode metastasis in breast cancer. J Exp Clin Cancer Res. 2004;23(1):121-5.

36. Rahimi Z, Yari K, Rahimi Z. Matrix metalloproteinase-9 -1562T allele and its combination with MMP-2 -735 C allele are risk factors for breast cancer. Asian Pac J Cancer Prev. 2015;16(3):1175-9. doi: 10.7314/apjcp.2015.16.3.1175.

37. Resler AJ, Malone KE, Johnson LG, Malkki M, Petersdorf EW, McKnight B, Madeleine MM. Genetic variation in TLR or NFkappaB pathways and the risk of breast cancer: a case-control study. BMC Cancer. 2013 May 1;13:219. doi: 10.1186/1471-2407-13-219.

38. Roehe AV, Frazzon AP, Agnes G, Damin AP, Hartman AA, et al. (2007) Detection of polymorphisms in the promoters of matrix metalloproteinases 2 and 9 genes in breast cancer in South Brazil: preliminary results. *Breast Cancer Research and Treatment* 102: 123–124.

39. Sadeghi M, Motovali Bashi M, Hojati Z. MMP-9 promoter polymorphism associated with tumor progression of breast cancer in Iranian population. *International Journal of Integrative Biology*. 2009;6(1):33–37.

40. Saeed HM, Alanazi MS, Alshahrani O, et al. Matrix metalloproteinase-2 C(−1306) T promoter polymorphism and breast cancer risk in the Saudi population. *Acta Biochim Pol.* 2013;**60**(3):405–409.

41. Shevchenko AV, Konenkov VI, Garbukov EIu, Stakheeva MN. Associating of polymorphism in the promoter regions of genes of metalloproteinase (*MMP2, MMP3, MMP9*) with options of the clinical course of breast cancer in Russian women. *Vopr Onkol*. 2014;60(5):630-635.

42. Slattery M.L., John E., Torres-Mejia G., Stern M., Lundgreen A., Hines L., Giuliano A., Baumgartner K., Herrick J., Wolff R.K. Matrix metalloproteinase genes are associated with breast cancer risk and survival: The Breast Cancer Health Disparities Study. *PLoS ONE.* 2013;8:e63165. doi: 10.1371/journal.pone.0063165.

43. Su CH, Lane HY, Hsiao CL, Liu LC, Ji HX, Li HT, Yen ST, Su CH, Hsia TC, Chang WS, Tsai CW, Bau DT. Matrix Metalloproteinase-1 Genetic Polymorphism in Breast Cancer in Taiwanese. Anticancer Res. 2016 Jul;36(7):3341-5.

44. Sui J, Huang J, Zhang Y. The *MMP-1* Gene rs1799750 Polymorphism Is Associated with Breast Cancer Risk. Genet Test Mol Biomarkers. 2021 Jul;25(7):496-503. doi: 10.1089/gtmb.2021.0016.

45. Toroghi, F., Mashayekhi, F., Montazeri, V., Saedi, H.S., Salehi, Z., 2017. Association of MMP-9 promoter polymorphism and breast cancer among Iranian patients. Eur. J. Oncol. 1 (22), 38–42.

46. Wang K, Zhou Y, Li G, Wen X, Kou Y, Yu J, He H, Zhao Q, Xue F, Wang J, Zhao X. MMP8 and MMP9 gene polymorphisms were associated with breast cancer risk in a Chinese Han population. Sci Rep. 2018 Sep 7;8(1):13422. doi: 10.1038/s41598-018-31664-3.

47. Xu T, Zhang S, Qiu D, Li X, Fan Y. Association between matrix metalloproteinase 9 polymorphisms and breast cancer risk: An updated meta-analysis and trial sequential analysis. Gene. 2020 Oct 30;759:144972. doi: 10.1016/j.gene.2020.144972.

48. Yan C, Sun C, Lu D, Zhao T, Ding X, Zamir I, Tang M, Shao C, Zhang F. Estimation of associations between MMP9 gene polymorphisms and breast cancer: Evidence from a meta-analysis. Int J Biol Markers. 2022 Feb 14:17246008221076145. doi: 10.1177/17246008221076145.

49. Yang L, Li N, Wang S, Kong Y, Tang H, Xie X, Xie X. Lack of association between the matrix metalloproteinase-2 -1306C>T polymorphism and breast cancer susceptibility: a meta-analysis. Asian Pac J Cancer Prev. 2014;15(12):4823-7. doi: 10.7314/apjcp.2014.15.12.4823.

50. Zagouri F, Sergentanis TN, Gazouli M, et al. MMP-2 -1306C>T polymorphism in breast cancer: a case-control study in a south European population. *Mol Biol Rep.* 2013;**40**(8):5035–5040.

51. Zhang X, Jin G, Li J, Zhang L. Association between four MMP-9 polymorphisms and breast cancer risk: a meta-analysis. *Med Sci Monit*. 2015;21:1115-1123. doi:10.12659/MSM.893890

52. Zhou P, Du LF, Lv GQ, Yu XM, Gu YL, Li JP, Zhang C. Current evidence on the relationship between four polymorphisms in the matrix metalloproteinases (MMP) gene and breast cancer risk: a meta-analysis. Breast Cancer Res Treat. 2011 Jun;127(3):813-8. doi: 10.1007/s10549-010-1294-0.

53. Zhou Y, Yu C, Miao X, Tan W, Liang G, Xiong P, Sun T, Lin D. Substantial reduction in risk of breast cancer associated with genetic polymorphisms in the promoters of the

matrix metalloproteinase-2 and tissue inhibitor of metalloproteinase-2 genes. Carcinogenesis. 2004;25(3):399-404. doi: 10.1093/carcin/bgh020.

54. Zhou Z, Ma X, Wang F, Sun L, Zhang G. A Matrix Metalloproteinase-1 Polymorphism, *MMP1-1607* (1G>2G), Is Associated with Increased Cancer Risk: A Meta-Analysis Including 21,327 Patients. Dis Markers. 2018;2018:7565834. doi: 10.1155/2018/7565834.
