# Supplementary material for: Matrix Metalloproteinase Gene Polymorphisms Are Associated with Breast Cancer in the Caucasian Women of Russia
Source: Int J Mol Sci. 2022 Oct 20;23(20):12638. doi: 10.3390/ijms232012638 (PMC9604098; doi:10.3390/ijms232012638)
Supplement: Supplementary file 1 [file ijms-23-12638-s001.zip › Suppl table s2.pdf]

Supplementary table S2

Genotype combinations associated with breast cancer*

Model	N	Genotype combinations	*beta*	P	Risk, High/Low
Two-order interaction models					
1	1	**GA (rs17577)xCC (rs3918242)**	**1.54**	**0.0001**	**H**
	2	**GG (rs17577)xCT (rs3918242)**	**1.54**	**0.0001**	**H**
	3	GA (rs17577)xCT (rs3918242)	-0.43	0.007	L
2	4	1G1G (rs1799750)xAA (rs17576)	0.50	0.010	L
	5	1G1G (rs1799750)xAG (rs17576)	-0.61	0.004	H
	6	1G2G (rs1799750)xAG (rs17576)	0.34	0.026	H
3	7	GA (rs17577)xAA (rs17576)	2.14	0.001	H
4	8	**1G1G (rs1799750)xTT (rs679620)**	**-0.79**	**0.0004**	**L**
5	9	CG (rs2250889)xCC (rs243865)	-0.62	0.011	L
Three-order interaction models					
1	1	GG (rs17577)xCT (rs3918242)xCC (rs1940475)	1.69	0.047	H
	2	GA (rs17577)xCC (rs3918242)xCT (rs1940475)	2.44	0.002	H
	3	GG (rs17577)xCT (rs3918242)xCT (rs1940475)	1.41	0.011	H
2	4	GA (rs17577)xCC (rs3918242)xCC (rs243865)	1.28	0.013	H
	5	GG (rs17577)xCT (rs3918242)xCC (rs243865)	2.34	0.003	H
	6	GA (rs17577)xCC (rs3918242)xCT (rs243865)	1.82	0.003	H
	7	GG (rs17577)xCT (rs3918242)xCT (rs243865)	1.25	0.018	H
	8	GA (rs17577)xCT (rs3918242)xCT (rs243865)	-0.70	0.007	L
3	9	GA (rs17577)xGG (rs3787268)xCC (rs3918242)	1.12	0.021	H
	10	GA (rs17577)xGA (rs3787268)xCC (rs3918242)	2.12	0.008	H
	11	GG (rs17577)xGG (rs3787268)xCT (rs3918242)	1.72	0.003	H
4	12	GA (rs17577)x1G1G (rs1799750)xCC (rs3918242)	1.33	0.044	H
	13	GA (rs17577)x1G2G (rs1799750) xCC (rs3918242)	1.94	0.004	H
	14	GA (rs17577)x1G1G (rs1799750)xCT (rs3918242)	-0.93	0.003	L
	15	GG (rs17577)x1G2G (rs1799750)xCT (rs3918242)	1.31	0.013	H
	16	GG (rs17577)x2G2G (rs1799750)xCT (rs3918242)	1.62	0.049	H
	17	GA (rs17577)x2G2G (rs1799750)xCT (rs3918242)	-0.79	0.020	L
5	18	GA (rs17577)xCC (rs3918242)xCC (rs679620)	1.72	0.041	H
	19	GG (rs17577)xCT (rs3918242)xCC (rs679620)	1.91	0.004	H
	20	GG (rs17577)xCT (rs3918242)xCT (rs679620)	1.05	0.050	H
	21	GA (rs17577)xCC (rs3918242)xTT (rs679620)	1.83	0.007	H
	22	GA (rs17577)xCT (rs3918242)xTT (rs679620)	-1.18	0.001	L
6	**23**	**GA (rs17577)xCC (rs2250889)xCC (rs3918242)**	**1.54**	**0.0004**	**H**
	**24**	**GG (rs17577)xCC (rs2250889)xCT (rs3918242)**	**1.61**	**0.0002**	**H**
	25	GA (rs17577)xCC (rs2250889)xCT (rs3918242)	-0.35	0.039	L
Four-order interaction models					
1	1	GG (rs17577)xAA (rs3787268)xCC (rs3918242)xCC (rs243865)	0.79	0.048	H
	2	GG (rs17577)xGG (rs3787268)xCT (rs3918242)xCC (rs243865)	2.56	0.017	H

	3	GA (rs17577)xAA (rs3787268)xCC (rs3918242)xCT (rs243865)	1.41	0.034	H
	4	GA (rs17577)xAA (rs3787268)xCT (rs3918242)xCT (rs243865)	-0.57	0.050	L
2	5	1G1G (rs1799750)xTT (rs3918249)xCT (rs1940475)xCC (rs243865)	0.74	0.028	H
	6	1G2G (rs1799750)xCC (rs3918249)xCT (rs1940475)xCC (rs243865)	0.94	0.038	H
	7	1G2G (rs1799750)xTC (rs3918249)xTT (rs1940475)xCC (rs243865)	0.83	0.019	H
	8	1G2G (rs1799750)xTC (rs3918249)xCT (rs1940475)xCT (rs243865)	0.93	0.007	H
Five-order interaction models					
1	1	CC (rs2250889)x1G1G (rs1799750)xTT (rs3918249)xCT (rs1940475)xCC (rs243865)	1.39	0.002	H
	2	CC (rs2250889)x1G2G (rs1799750)xTC (rs3918249)xTT (rs1940475)xCC (rs243865)	0.91	0.012	H
	3	CC (rs2250889)x1G1G (rs1799750)xTT (rs3918249)xCT (rs1940475)xCT (rs243865)	1.08	0.040	H
	4	CC (rs2250889)x1G2G (rs1799750)xTC (rs3918249)xCT (rs1940475)xCT (rs243865)	1.01	0.005	H

***** Genotype combinations are derived from the interaction models obtained by the MB-MDR method and described in tables 4
