# Supplementary material for: Matrix Metalloproteinase Gene Polymorphisms Are Associated with Breast Cancer in the Caucasian Women of Russia
Source: Int J Mol Sci. 2022 Oct 20;23(20):12638. doi: 10.3390/ijms232012638 (PMC9604098; doi:10.3390/ijms232012638)
Supplement: Supplementary file 1 [file ijms-23-12638-s001.zip › Suppl table s3.pdf]

Supplementary Table S3. Non-synonymous BC-associated SNPs of the M
(predictive potential were analyzed using the SIFT tool (https://sift.bii.a

SNP	Amino acid change	PolyPh
		PolyPhen-2 scope
rs1940475, C>T	Lys87Glu MMP8	0.009
rs3765620, G>A (linked with rs1940475, r2=0.85 and D'=1.00)	Thr32Ile MMP8	0
rs679620, C>T	Lys45Glu MMP3	0
rs17576, A>G	Gln279Arg MMP9	0.004
rs2250889, C>G	Arg574Pro MMP9	0
rs17577, G>A	Arg668Gln MMP9	0.001

MMP genes

-star.edu.sg/) and PolyPhen-2 tool (http://genetics.bwh.harvard.edu/pph2/))

en-2 tool	SIFT tool	
PolyPhen-2prediction	SIFT scope	SIFT prediction
benign	0.12	tolerated
benign	0.33	tolerated
benign	1	tolerated
benign	0.29	tolerated
benign	1	tolerated
benign	0.65	tolerated
