# Supplementary material for: Matrix Metalloproteinase Gene Polymorphisms Are Associated with Breast Cancer in the Caucasian Women of Russia
Source: Int J Mol Sci. 2022 Oct 20;23(20):12638. doi: 10.3390/ijms232012638 (PMC9604098; doi:10.3390/ijms232012638)
Supplement: Supplementary file 1 [file ijms-23-12638-s001.zip › Suppl table s4.pdf]

Supplementary table S4 Regulatory effects of the ten BC-associated SNPs of the *M*

(HaploReg, v4.1, http://archive.broadinstitute.org/mammals/haploreg/haploreg.php)

Query SNP: rs1940475 and variants with r^2 >= 0.8

chr	pos (hg38)	LD (r²)	LD (D')	variant	Ref	Alt	AFR freq	AMR freq	ASN freq	EUR freq	GERP cons	SiPhy cons	Promoter histone marks
11	102721214	0.85	1	rs2186602	T	C	0.81	0.59	0.60	0.57			
11	102722311	0.85	1	rs948218	T	A	0.81	0.59	0.60	0.57			
11	102722322	1	1	rs1940474	G	T	0.65	0.54	0.59	0.53			
11	102722517	1	1	rs1940475	T	C	0.72	0.54	0.59	0.53			
11	102723248	0.85	1	rs7123658	A	T	0.81	0.59	0.60	0.57			
11	102723645	0.85	1	rs6590983	A	C	0.81	0.59	0.60	0.57			
11	102724404	1	1	rs1939012	T	C	0.76	0.54	0.59	0.53			
11	102724761	0.85	1	rs3765620	G	A	0.81	0.59	0.60	0.57			
11	102725749	0.84	1	rs11225395	A	G	0.81	0.59	0.60	0.57			
11	102726340	0.84	1	rs33952053	TG	T	0.82	0.59	0.60	0.57			
11	102727079	0.84	1	rs1939013	T	C	0.83	0.59	0.60	0.57			
11	102727181	0.84	1	rs1939014	A	T	0.81	0.59	0.60	0.57			
11	102727628	0.84	1	rs3758857	T	C	0.81	0.59	0.60	0.57			
11	102727726	0.84	1	rs3758858	G	A	0.81	0.59	0.60	0.57			BLD, MUS
11	102727749	0.99	1	rs3758859	C	A	0.65	0.54	0.59	0.53			BLD, MUS
11	102727924	0.84	1	rs6590985	T	C	0.81	0.59	0.60	0.57			BLD, MUS
11	102728128	0.84	1	rs6590986	C	T	0.81	0.59	0.60	0.57			
11	102729275	0.84	1	rs10750653	A	G	0.82	0.59	0.60	0.57			

Query SNP: rs1799750 and variants with r^2 >= 0.8

chr	pos (hg38)	LD (r²)	LD (D')	variant	Ref	Alt	AFR freq	AMR freq	ASN freq	EUR freq	GERP cons	SiPhy cons	Promoter histone marks
11	102799764	1	1	rs1799750	TC	T	0.55	0.44	0.33	0.49			

Query SNP: rs679620 and variants with r^2 >= 0.8

chr	pos (hg38)	LD (r²)	LD (D')	variant	Ref	Alt	AFR freq	AMR freq	ASN freq	EUR freq	GERP cons	SiPhy cons	Promoter histone marks
11	102807707	0.82	-0.92	rs526215	C	T	0.04	0.34	0.15	0.45			ESC, IPSC, LNG

11	102808321	0.81	-0.92	rs1144396	C	A	0.51	0.37	0.33	0.45	4 tissues
11	102808927	0.82	-0.92	rs565082	T	C	0.41	0.37	0.33	0.45	
11	102811772	0.81	-0.91	rs502174	A	C	0.42	0.37	0.32	0.45	
11	102816687	0.83	-0.93	rs10791596	T	C	0.41	0.36	0.33	0.45	
11	102819770	0.89	-0.96	rs2097248	C	T	0.41	0.37	0.33	0.47	
11	102819867	0.84	-0.92	rs200587503	T	TG	0.42	0.36	0.33	0.46	
11	102820431	0.9	-0.96	rs756544	G	C	0.34	0.37	0.33	0.46	
11	102820659	0.91	-0.97	rs12804136	G	A	0.41	0.37	0.33	0.47	
11	102820751	0.9	-0.97	rs11225434	T	C	0.39	0.37	0.33	0.47	
11	102821493	0.91	-0.97	rs7117013	A	G	0.43	0.38	0.33	0.47	
11	102822132	0.91	-0.97	rs10895364	C	T	0.43	0.39	0.33	0.47	
11	102822879	0.92	-0.98	rs12801529	T	A	0.40	0.38	0.33	0.47	
11	102823462	0.92	-0.98	rs10895365	C	T	0.39	0.37	0.33	0.47	
11	102825487	0.89	-0.96	rs6590993	G	T	0.39	0.37	0.33	0.47	
11	102825901	0.92	-0.98	rs6590994	C	T	0.34	0.37	0.33	0.47	
11	102826358	0.96	-0.98	rs10895366	G	A	0.03	0.34	0.15	0.46	
11	102826390	0.96	-0.98	rs7126392	G	A	0.38	0.36	0.32	0.46	
11	102826825	0.94	-0.97	rs2212637	C	T	0.41	0.37	0.33	0.46	
11	102827605	0.96	-0.98	rs12792871	C	G	0.03	0.35	0.15	0.46	
11	102827993	0.95	-0.98	rs7926920	G	A	0.40	0.37	0.33	0.46	
11	102828729	0.92	-0.96	rs7946057	A	T	0.40	0.37	0.33	0.46	
11	102830290	0.98	-1	rs1010698	G	T	0.40	0.37	0.33	0.46	
11	102831127	0.98	-1	rs2155013	T	C	0.40	0.37	0.33	0.46	FAT, GI
11	102831532	0.98	-1	rs4754884	G	A	0.40	0.37	0.33	0.46	22 tissues
11	102832897	0.99	1	rs635746	A	G	0.58	0.62	0.67	0.54	GI
11	102833340	0.99	1	rs633962	C	T	0.56	0.62	0.68	0.54	GI
11	102833449	0.99	1	rs666734	G	C	0.58	0.62	0.67	0.54	GI
11	102834166	0.99	1	rs595840	A	G	0.71	0.64	0.67	0.54	GI
11	102836608	0.99	1	rs639752	C	A	0.56	0.62	0.67	0.54	
11	102837183	0.98	0.99	rs575027	A	G	0.58	0.62	0.67	0.54	
11	102838694	0.98	0.99	rs520540	A	G	0.58	0.62	0.68	0.54	
11	102840607	1	1	rs591058	T	C	0.69	0.63	0.67	0.54	
11	102842734	1	1	rs602128	A	G	0.57	0.62	0.67	0.54	
11	102842889	1	1	rs679620	T	C	0.71	0.64	0.67	0.54	

11	102843046	1	1	rs678815	G	C	0.69	0.63	0.67	0.54			
11	102843985	0.99	0.99	rs617819	C	G	0.71	0.64	0.67	0.54			
11	102844950	0.99	0.99	rs632478	T	G	0.69	0.63	0.67	0.54			
11	102845590	0.99	0.99	rs645419	A	G	0.69	0.63	0.67	0.54			
11	102846249	0.95	0.99	rs573521	A	G	0.68	0.63	0.66	0.53			CRVX

Query SNP: rs243865 and variants with r^2 >= 0.8

chr	pos (hg38)	LD (r^2)	LD (D')	variant	Ref	Alt	AFR freq	AMR freq	ASN freq	EUR freq	GERP cons	SiPhy cons	Promoter histone marks
16	55477625	0.99	1	rs243866	G	A	0.01	0.19	0.09	0.28			5 tissues
16	55477894	1	1	rs243865	C	T	0.02	0.19	0.09	0.27			4 tissues
16	55478410	0.99	1	rs243864	T	G	0.02	0.19	0.09	0.28			5 tissues
16	55479711	0.92	1	rs243862	C	T	0.02	0.18	0.09	0.26			24 tissues

Query SNP: rs3918242 and variants with r^2 >= 0.8

chr	pos (hg38)	LD (r^2)	LD (D')	variant	Ref	Alt	AFR freq	AMR freq	ASN freq	EUR freq	GERP cons	SiPhy cons	Promoter histone marks
20	45986352	0.89	0.95	rs142450006	TTTTC	T	0.07	0.10	0.17	0.17			
20	45986358	0.86	0.95	rs201501785	T	TTC	0.07	0.10	0.17	0.17			
20	45995328	0.95	0.98	rs1888235	C	T	0.13	0.09	0.18	0.17			
20	46002014	0.95	0.98	rs6073984	A	G	0.13	0.09	0.17	0.17			
20	46003728	0.86	0.98	rs6065912	G	A	0.11	0.09	0.08	0.16			
20	46007096	1	1	rs3918241	T	A	0.13	0.09	0.17	0.17			
20	46007337	1	1	rs3918242	C	T	0.12	0.09	0.17	0.17			
20	46011053	0.99	1	rs2274755	G	T	0.16	0.10	0.16	0.18			15 tissues
20	46011936	0.99	1	rs2236416	A	G	0.12	0.09	0.16	0.18			15 tissues
20	46014472	0.98	1	rs17577	G	A,C	0.17	0.10	0.18	0.18			21 tissues
20	46014953	0.98	1	rs3918261	A	G	0.21	0.10	0.18	0.18			5 tissues
20	46016326	0.98	1	rs13925	G	A	0.15	0.10	0.18	0.18			
20	46016700	0.96	1	rs3918270	G	A	0.17	0.10	0.18	0.18			
20	46016954	0.93	0.97	rs3918271	G	A	0.17	0.10	0.18	0.18			

20	46020003	0.82	1	rs66553311	C	G	0.50	0.27	0.41	0.20			
20	46021679	0.89	0.98	rs10432735	A	T	0.18	0.11	0.18	0.19			

Query SNP: rs3918249 and variants with r^2 >= 0.8

chr	pos (hg38)	LD (r²)	LD (D')	variant	Ref	Alt	AFR freq	AMR freq	ASN freq	EUR freq	GERP cons	SiPhy cons	Promoter histone marks
20	45987758	0.83	0.94	rs6017721	A	G	0.71	0.34	0.75	0.41			SKIN
20	46005037	0.94	0.99	rs3848721	C	T	0.49	0.29	0.72	0.40			9 tissues
20	46005181	0.94	0.99	rs3848722	A	G	0.72	0.32	0.72	0.40			11 tissues
20	46005911	0.9	0.96	rs4810482	T	C	0.72	0.32	0.72	0.39			BRN, GI, ADRL
20	46006273	0.85	0.99	rs3761158	G	A	0.73	0.43	0.76	0.42			
20	46006318	0.95	0.99	rs3761159	C	T	0.43	0.29	0.72	0.40			
20	46006464	0.99	0.99	rs6065913	C	T	0.34	0.28	0.72	0.39			BLD
20	46006977	0.99	0.99	rs3918240	C	T	0.34	0.28	0.72	0.39			
20	46009497	1	1	rs3918249	T	C	0.56	0.31	0.72	0.39			4 tissues
20	46010142	0.83	0.92	rs3918251	A	G	0.52	0.30	0.64	0.40			4 tissues
20	46011586	0.99	0.99	rs17576	A	G	0.35	0.28	0.72	0.39			17 tissues
20	46014194	0.82	-0.98	rs13969	A	C	0.38	0.49	0.24	0.57			23 tissues

Query SNP: rs17576 and variants with r^2 >= 0.8

chr	pos (hg38)	LD (r²)	LD (D')	variant	Ref	Alt	AFR freq	AMR freq	ASN freq	EUR freq	GERP cons	SiPhy cons	Promoter histone marks
20	45987758	0.82	0.94	rs6017721	A	G	0.71	0.34	0.75	0.41			SKIN
20	46005037	0.92	0.99	rs3848721	C	T	0.49	0.29	0.72	0.40			9 tissues
20	46005181	0.92	0.99	rs3848722	A	G	0.72	0.32	0.72	0.40			11 tissues
20	46005911	0.89	0.96	rs4810482	T	C	0.72	0.32	0.72	0.39			BRN, GI, ADRL
20	46006273	0.84	0.99	rs3761158	G	A	0.73	0.43	0.76	0.42			
20	46006318	0.94	0.99	rs3761159	C	T	0.43	0.29	0.72	0.40			
20	46006464	0.98	0.99	rs6065913	C	T	0.34	0.28	0.72	0.39			BLD

20	46006977	0.98	0.99	rs3918240	C	T	0.34	0.28	0.72	0.39			
20	46009497	0.99	0.99	rs3918249	T	C	0.56	0.31	0.72	0.39			4 tissues
20	46010142	0.84	0.93	rs3918251	A	G	0.52	0.30	0.64	0.40			4 tissues
20	46011586	1	1	rs17576	A	G	0.35	0.28	0.72	0.39			17 tissues
20	46014194	0.83	-0.98	rs13969	A	C	0.38	0.49	0.24	0.57			23 tissues

Query SNP: rs3787268 and variants with r^2 >= 0.8

chr	pos (hg38)	LD (r^2)	LD (D')	variant	Ref	Alt	AFR freq	AMR freq	ASN freq	EUR freq	GERP cons	SiPhy cons	Promoter histone marks
20	45991015	0.87	0.94	rs35610751	C	G	0.10	0.17	0.35	0.21			
20	45991065	0.86	0.94	rs6073980	G	A	0.13	0.17	0.35	0.22			
20	46001252	0.89	0.98	rs6073983	A	T	0.09	0.16	0.36	0.22			FAT, BLD
20	46006183	0.98	0.99	rs3761157	C	T	0.09	0.16	0.36	0.21			
20	46013092	1	1	rs3787268	G	A	0.09	0.16	0.36	0.21			BLD, SKIN
20	46015131	1	1	rs3918262	A	G	0.09	0.16	0.36	0.21			LIV
20	46022571	0.95	0.98	rs6073989	G	T	0.09	0.16	0.36	0.21			24 tissues
20	46023675	0.95	0.98	rs16991010	A	C	0.09	0.16	0.36	0.21			
20	46024480	0.95	0.98	rs6130998	C	T	0.09	0.16	0.37	0.21			
20	46026853	0.95	0.98	rs6130999	G	A	0.09	0.16	0.37	0.21			
20	46027473	0.95	0.98	rs6073991	A	G	0.09	0.16	0.37	0.21			
20	46031067	0.91	0.97	rs13039389	G	C	0.08	0.16	0.37	0.22			ESDR, IPSC, BRN
20	46032717	0.94	0.98	rs6131001	C	T	0.08	0.16	0.37	0.21			BRN

Query SNP: rs2250889 and variants with r^2 >= 0.8

chr	pos (hg38)	LD (r^2)	LD (D')	variant	Ref	Alt	AFR freq	AMR freq	ASN freq	EUR freq	GERP cons	SiPhy cons	Promoter histone marks
20	46013767	1	1	rs2250889	G	C	0.82	0.80	0.76	0.95			24 tissues
20	46024910	0.92	0.97	rs6094239	G	A	0.82	0.80	0.77	0.95			

Query SNP: rs17577 and variants with r^2 >= 0.8

chr	pos (hg38)	LD (r²)	LD (D')	variant	Ref	Alt	AFR freq	AMR freq	ASN freq	EUR freq	GERP cons	SiPhy cons	Promoter histone marks
20	45986352	0.88	0.95	rs142450006	TTTTC	T	0.07	0.10	0.17	0.17			
20	45986358	0.84	0.95	rs201501785	T	TTC	0.07	0.10	0.17	0.17			
20	45995328	0.94	0.98	rs1888235	C	T	0.13	0.09	0.18	0.17			
20	46002014	0.94	0.98	rs6073984	A	G	0.13	0.09	0.17	0.17			
20	46003728	0.84	0.98	rs6065912	G	A	0.11	0.09	0.08	0.16			
20	46007096	0.98	1	rs3918241	T	A	0.13	0.09	0.17	0.17			
20	46007337	0.98	1	rs3918242	C	T	0.12	0.09	0.17	0.17			
20	46011053	0.99	1	rs2274755	G	T	0.16	0.10	0.16	0.18			15 tissues
20	46011936	0.99	1	rs2236416	A	G	0.12	0.09	0.16	0.18			15 tissues
20	46014472	1	1	rs17577	G	A,C	0.17	0.10	0.18	0.18			21 tissues
20	46014953	1	1	rs3918261	A	G	0.21	0.10	0.18	0.18			5 tissues
20	46016326	1	1	rs13925	G	A	0.15	0.10	0.18	0.18			
20	46016700	0.98	1	rs3918270	G	A	0.17	0.10	0.18	0.18			
20	46016954	0.95	0.97	rs3918271	G	A	0.17	0.10	0.18	0.18			
20	46020003	0.84	1	rs66553311	C	G	0.50	0.27	0.41	0.20			
20	46021679	0.91	0.98	rs10432735	A	T	0.18	0.11	0.18	0.19			

MP gene and SNPs in high LD (r2≥0.80)

Enhancer histone marks	DNAse	Proteins bound	Motifs changed	NHGRI/EBI GWAS hits	GRASP QTL hits	Selected eQTL hits	GENCODE genes	RefSeq genes
BLD			5 altered motifs			3 hits	MMP8	MMP8
BLD			TCF4			3 hits	MMP8	MMP8
BLD			Fox,Foxi1,Foxj2			2 hits	MMP8	MMP8
BLD	BLD,BLD		CIZ,Myc,NF-AT1		1 hit	2 hits	MMP8	MMP8
BLD						3 hits	MMP8	MMP8
BLD			27 altered motifs			3 hits	MMP8	MMP8
BLD			HNF1	1 hit		2 hits	MMP8	MMP8
BLD, SKIN	SKIN		Foxp1,Sox		1 hit	3 hits	MMP8	MMP8
10 tissues	SKIN					3 hits	MMP8	794bp 5' of MMP8
BLD, SKIN			Pax-4				MMP8	1.4kb 5' of MMP8
BLD, SPLN	KID		Foxa			3 hits	28bp 5' of MMP8	2.1kb 5' of MMP8
BLD, SPLN			4 altered motifs			3 hits	130bp 5' of MMP8	2.2kb 5' of MMP8
7 tissues	BLD		EWSR1-FLI1,Pax-5,STAT			3 hits	577bp 5' of MMP8	2.7kb 5' of MMP8
7 tissues	SKIN,BLD		4 altered motifs			3 hits	675bp 5' of MMP8	2.8kb 5' of MMP8
7 tissues	SKIN,BLD		HNF4,Ncx			2 hits	698bp 5' of MMP8	2.8kb 5' of MMP8
6 tissues	8 tissues		GR,NRSF			3 hits	873bp 5' of MMP8	3kb 5' of MMP8
6 tissues	20 tissues	4 bound proteins	19 altered motifs			3 hits	1.1kb 5' of MMP8	3.2kb 5' of MMP8
	ESDR		DMRT3,Maf			2 hits	2.2kb 5' of MMP8	4.3kb 5' of MMP8

Enhancer histone marks	DNAse	Proteins bound	Motifs changed	NHGRI/EBI GWAS hits	GRASP QTL hits	Selected eQTL hits	GENCODE genes	RefSeq genes
6 tissues	5 tissues	CFOS,GATA2	21 altered motifs			8 hits	1.6kb 5' of MMP1	LOC100288077

Enhancer histone marks	DNAse	Proteins bound	Motifs changed	NHGRI/EBI GWAS hits	GRASP QTL hits	Selected eQTL hits	GENCODE genes	RefSeq genes
ESC, IPSC						3 hits	9.5kb 5' of MMP1	LOC100288077

ESC, ESDR, IPSC	5 tissues	CTCF	NRSF,Sin3Ak-20		3 hits	10kb 5' of MMP1	LOC100288077
					3 hits	11kb 5' of MMP1	LOC100288077
GI			HDAC2,Irf,p300		3 hits	14kb 5' of MMP1	LOC100288077
			4 altered motifs		3 hits	19kb 5' of MMP1	LOC100288077
	OVRY,GI,LIV	FOXA1,FOXA2	4 altered motifs		3 hits	16kb 3' of MMP3	LOC100288077
			Ets,HNF4,NF-kappaB		3 hits	16kb 3' of MMP3	LOC100288077
	BLD		HNF1		3 hits	15kb 3' of MMP3	LOC100288077
			7 altered motifs		3 hits	15kb 3' of MMP3	LOC100288077
			Foxp3,Obox6	1 hit	3 hits	15kb 3' of MMP3	LOC100288077
			4 altered motifs		3 hits	14kb 3' of MMP3	LOC100288077
			EWSR1-FLI1		3 hits	14kb 3' of MMP3	LOC100288077
					3 hits	13kb 3' of MMP3	LOC100288077
	LNG		4 altered motifs		3 hits	12kb 3' of MMP3	LOC100288077
			5 altered motifs		3 hits	10kb 3' of MMP3	LOC100288077
					3 hits	9.9kb 3' of MMP3	LOC100288077
			Sin3Ak-20		3 hits	9.4kb 3' of MMP3	LOC100288077
			Glis2,MAZR,Zic		3 hits	9.4kb 3' of MMP3	LOC100288077
			ZBRK1,Zfx		3 hits	9kb 3' of MMP3	LOC100288077
			ATF3		3 hits	8.2kb 3' of MMP3	LOC100288077
			4 altered motifs		3 hits	7.8kb 3' of MMP3	LOC100288077
			7 altered motifs		3 hits	7.1kb 3' of MMP3	LOC100288077
	SKIN,BRST		4 altered motifs		3 hits	5.5kb 3' of MMP3	LOC100288077
FAT	KID		TCF4		3 hits	4.7kb 3' of MMP3	LOC100288077
ESC, IPSC, SKIN	31 tissues	CTCF,RAD21,SMC3	NF-kappaB		3 hits	4.3kb 3' of MMP3	LOC100288077
	GI		4 altered motifs		3 hits	2.9kb 3' of MMP3	LOC100288077
					3 hits	2.5kb 3' of MMP3	LOC100288077
			Gfi1,SIX5		3 hits	2.4kb 3' of MMP3	LOC100288077
			5 altered motifs		3 hits	1.6kb 3' of MMP3	LOC100288077
13 tissues	11 tissues	P300,TBP,STAT3			3 hits	MMP3	MMP3
10 tissues			Maf,PRDM1,Spz1		3 hits	MMP3	MMP3
					3 hits	MMP3	MMP3
			4 altered motifs		3 hits	MMP3	MMP3
			ATF3,PU.1		3 hits	MMP3	MMP3
			p300		3 hits	MMP3	MMP3

	LNG,SKIN,SKIN		Hoxd10,Pou3f2			3 hits	MMP3	MMP3
FAT, SKIN	SKIN		Mef2			3 hits	181bp 5' of MMP3	373bp 5' of MMP3
			ZEB1			3 hits	1.1kb 5' of MMP3	1.3kb 5' of MMP3
			Hsf,STAT			3 hits	1.8kb 5' of MMP3	2kb 5' of MMP3
BRST, SKIN		ZNF263	8 altered motifs	1 hit		3 hits	2.4kb 5' of MMP3	2.6kb 5' of MMP3

Enhancer histone marks	DNAse	Proteins bound	Motifs changed	NHGRI/EBI GWAS hits	GRASP QTL hits	Selected eQTL hits	GENCODE genes	RefSeq genes
18 tissues	MUS,MUS,SKIN		5 altered motifs		1 hit	1 hit	MMP2	1.5kb 5' of MMP2
15 tissues	ESDR,IPSC,MUS		Myf		1 hit	1 hit	MMP2	1.3kb 5' of MMP2
14 tissues	4 tissues		5 altered motifs		1 hit	1 hit	MMP2	757bp 5' of MMP2
	19 tissues		14 altered motifs				MMP2	MMP2

Enhancer histone marks	DNAse	Proteins bound	Motifs changed	NHGRI/EBI GWAS hits	GRASP QTL hits	Selected eQTL hits	GENCODE genes	RefSeq genes
			8 altered motifs			6 hits	14kb 5' of ZNF335	14kb 5' of ZNF335
			9 altered motifs			5 hits	14kb 5' of ZNF335	14kb 5' of ZNF335
10 tissues	CRVX	MAFK	8 altered motifs		4 hits	10 hits	14kb 5' of MMP9	14kb 5' of MMP9
BLD, THYM	ESDR				2 hits	6 hits	6.9kb 5' of MMP9	6.9kb 5' of MMP9
6 tissues	ESDR,BLD		Pax-5		1 hit	9 hits	5.2kb 5' of MMP9	5.2kb 5' of MMP9
BLD, THYM, SPLN			24 altered motifs		1 hit	9 hits	1.8kb 5' of MMP9	1.8kb 5' of MMP9
BLD, THYM, SPLN	IPSC		4 altered motifs		1 hit	6 hits	1.6kb 5' of MMP9	1.6kb 5' of MMP9
	7 tissues				2 hits	9 hits	MMP9	MMP9
BLD	33 tissues	6 bound proteins	NF-Y		1 hit	9 hits	MMP9	MMP9
SKIN	22 tissues	4 bound proteins			1 hit	6 hits	MMP9	MMP9
BLD	BLD,BRST		Foxo,GR		6 hits	9 hits	MMP9	MMP9
			4 altered motifs		1 hit	6 hits	MMP9	MMP9
			5 altered motifs		1 hit	9 hits	RP11-465L10.10	138bp 3' of MMP9
			8 altered motifs			6 hits	RP11-465L10.10	392bp 3' of MMP9

			HNF4,Zbtb3			7 hits	RP11-465L10.10	1.7kb 5' of SLC12A5
	21 tissues	7 bound proteins	Pax-4,ZNF219,Zfp281		1 hit	5 hits	RP11-465L10.10	9bp 5' of SLC12A5

Enhancer histone marks	DNAse	Proteins bound	Motifs changed	NHGRI/EBI GWAS hits	GRASP QTL hits	Selected eQTL hits	GENCODE genes	RefSeq genes
7 tissues	4 tissues		4 altered motifs		2 hits	20 hits	16kb 5' of ZNF335	16kb 5' of ZNF335
15 tissues	20 tissues		SP1,STAT,Zfp281			9 hits	3.9kb 5' of MMP9	3.9kb 5' of MMP9
14 tissues	19 tissues		4 altered motifs			9 hits	3.7kb 5' of MMP9	3.7kb 5' of MMP9
11 tissues	ESC,BLD		AP-1		2 hits	10 hits	3kb 5' of MMP9	3kb 5' of MMP9
6 tissues	BLD		11 altered motifs			11 hits	2.6kb 5' of MMP9	2.6kb 5' of MMP9
6 tissues	4 tissues					9 hits	2.6kb 5' of MMP9	2.6kb 5' of MMP9
4 tissues	BLD,BLD,BLD					11 hits	2.4kb 5' of MMP9	2.4kb 5' of MMP9
BLD, THYM, SPLN	BLD		6 altered motifs			11 hits	1.9kb 5' of MMP9	1.9kb 5' of MMP9
BLD	BLD,BLD		4 altered motifs			9 hits	MMP9	MMP9
	BLD,LIV		5 altered motifs			8 hits	MMP9	MMP9
4 tissues	ESC		Pax-4			12 hits	MMP9	MMP9
SKIN	24 tissues	7 bound proteins	4 altered motifs			7 hits	MMP9	MMP9

Enhancer histone marks	DNAse	Proteins bound	Motifs changed	NHGRI/EBI GWAS hits	GRASP QTL hits	Selected eQTL hits	GENCODE genes	RefSeq genes
7 tissues	4 tissues		4 altered motifs		2 hits	20 hits	16kb 5' of ZNF335	16kb 5' of ZNF335
15 tissues	20 tissues		SP1,STAT,Zfp281			9 hits	3.9kb 5' of MMP9	3.9kb 5' of MMP9
14 tissues	19 tissues		4 altered motifs			9 hits	3.7kb 5' of MMP9	3.7kb 5' of MMP9
11 tissues	ESC,BLD		AP-1		2 hits	10 hits	3kb 5' of MMP9	3kb 5' of MMP9
6 tissues	BLD		11 altered motifs			11 hits	2.6kb 5' of MMP9	2.6kb 5' of MMP9
6 tissues	4 tissues					9 hits	2.6kb 5' of MMP9	2.6kb 5' of MMP9
4 tissues	BLD,BLD,BLD					11 hits	2.4kb 5' of MMP9	2.4kb 5' of MMP9

BLD, THYM, SPLN	BLD		6 altered motifs			11 hits	1.9kb 5' of MMP9	1.9kb 5' of MMP9
BLD	BLD,BLD		4 altered motifs			9 hits	MMP9	MMP9
	BLD,LIV		5 altered motifs			8 hits	MMP9	MMP9
4 tissues	ESC		Pax-4			12 hits	MMP9	MMP9
SKIN	24 tissues	7 bound proteins	4 altered motifs			7 hits	MMP9	MMP9

**Enhancer histone marks**	**DNAse**	**Proteins bound**	**Motifs changed**	**NHGRI/EBI GWAS hits**	**GRASP QTL hits**	**Selected eQTL hits**	**GENCODE genes**	**RefSeq genes**
			Nkx3			2 hits	18kb 5' of MMP9	18kb 5' of MMP9
			BDP1,LUN-1			2 hits	18kb 5' of MMP9	18kb 5' of MMP9
9 tissues	BLD,BRN		Arid5b			4 hits	7.7kb 5' of MMP9	7.7kb 5' of MMP9
6 tissues	4 tissues		9 altered motifs			3 hits	2.7kb 5' of MMP9	2.7kb 5' of MMP9
BLD	BLD,BLD		6 altered motifs			4 hits	MMP9	MMP9
BLD	BLD,LIV	9 bound proteins	9 altered motifs			3 hits	MMP9	MMP9
	16 tissues		5 altered motifs			3 hits	RP11-465L10.7	SLC12A5
			Mrg			3 hits	RP11-465L10.7	SLC12A5
	BLD,BLD,LNG		CDP			3 hits	RP11-465L10.7	SLC12A5
		NRSF	EBF,SZF1-1			2 hits	RP11-465L10.7	SLC12A5
	49 tissues	NRSF,ZNF143,BCL3	SZF1-1,T3R		2 hits	3 hits	RP11-465L10.7	SLC12A5
			BAF155,TFIIA			2 hits	SLC12A5	SLC12A5
			4 altered motifs			2 hits	SLC12A5	SLC12A5

**Enhancer histone marks**	**DNAse**	**Proteins bound**	**Motifs changed**	**NHGRI/EBI GWAS hits**	**GRASP QTL hits**	**Selected eQTL hits**	**GENCODE genes**	**RefSeq genes**
	15 tissues	CTCF	NRSF				MMP9	MMP9
			7 altered motifs				RP11-465L10.7	SLC12A5

Enhancer histone marks	DNAse	Proteins bound	Motifs changed	NHGRI/EBI GWAS hits	GRASP QTL hits	Selected eQTL hits	GENCODE genes	RefSeq genes
			8 altered motifs			6 hits	14kb 5' of ZNF335	14kb 5' of ZNF335
			9 altered motifs			5 hits	14kb 5' of ZNF335	14kb 5' of ZNF335
10 tissues	CRVX	MAFK	8 altered motifs		4 hits	10 hits	14kb 5' of MMP9	14kb 5' of MMP9
BLD, THYM	ESDR				2 hits	6 hits	6.9kb 5' of MMP9	6.9kb 5' of MMP9
6 tissues	ESDR,BLD		Pax-5		1 hit	9 hits	5.2kb 5' of MMP9	5.2kb 5' of MMP9
BLD, THYM, SPLN			24 altered motifs		1 hit	9 hits	1.8kb 5' of MMP9	1.8kb 5' of MMP9
BLD, THYM, SPLN	IPSC		4 altered motifs		1 hit	6 hits	1.6kb 5' of MMP9	1.6kb 5' of MMP9
	7 tissues				2 hits	9 hits	MMP9	MMP9
BLD	33 tissues	6 bound proteins	NF-Y		1 hit	9 hits	MMP9	MMP9
SKIN	22 tissues	4 bound proteins			1 hit	6 hits	MMP9	MMP9
BLD	BLD,BRST		Foxo,GR		6 hits	9 hits	MMP9	MMP9
			4 altered motifs		1 hit	6 hits	MMP9	MMP9
			5 altered motifs		1 hit	9 hits	RP11-465L10.10	138bp 3' of MMP9
			8 altered motifs			6 hits	RP11-465L10.10	392bp 3' of MMP9
			HNF4,Zbtb3			7 hits	RP11-465L10.10	1.7kb 5' of SLC12A5
	21 tissues	7 bound proteins	Pax-4,ZNF219,Zfp281		1 hit	5 hits	RP11-465L10.10	9bp 5' of SLC12A5

dbSNP func annot
intronic
intronic
intronic
missense
intronic
intronic
intronic
missense

dbSNP func annot
intronic

dbSNP func annot
intronic

intronic
intronic
intronic
intronic
intronic
intronic
intronic
intronic
intronic
intronic
intronic
intronic
intronic
intronic
intronic
intronic
intronic
intronic
intronic
intronic
intronic
intronic
intronic
intronic
intronic
intronic
intronic
intronic
intronic
synonymous
intronic
synonymous
missense

intronic
**dbSNP**
**func annot**
intronic
**dbSNP**
**func annot**
intronic
intronic
missense
intronic
synonymous
intronic
intronic

intronic

**dbSNP**
**func annot**
intronic
intronic
missense
synonymous

**dbSNP**
**func annot**

intronic
intronic
missense
synonymous

**dbSNP**
**func annot**
intronic
intronic
intronic
intronic
intronic
intronic
intronic
intronic
intronic

**dbSNP**
**func annot**
missense
intronic

dbSNP func annot
intronic
intronic
missense
intronic
synonymous
intronic
intronic
intronic
