# Supplementary material for: Matrix Metalloproteinase Gene Polymorphisms Are Associated with Breast Cancer in the Caucasian Women of Russia
Source: Int J Mol Sci. 2022 Oct 20;23(20):12638. doi: 10.3390/ijms232012638 (PMC9604098; doi:10.3390/ijms232012638)
Supplement: Supplementary file 1 [file ijms-23-12638-s001.zip › Suppl table s5.pdf]

Supplementary table S5 Effect of the BC-associated *MMP* gene polymorphisms c

(HaploReg, v4.1, http://archive.broadinstitute.org/mammals/haploreg/haploreg.php)

SNPs	transcription factors	log-odds (LOD) коэфф.		ΔLOD
		reference (ref) allele	alternative (alt) allele	
rs1940475 (ref:T, alt:C)	CIZ	12.6	0.7	-11.9
	Myc	12.5	3.3	-9.2
	NF-AT1	0.7	12.6	11.9
rs1799750 (ref:2G, alt:1G)	AP-1	6.4	-5.5	-11.9
	CHX10	-16.3	-15.3	1.0
	DMRT2	-18.3	-10.9	7.4
	Dbx1	10.0	9.5	-0.5
	En-1	5.8	5.5	-0.3
	Ets	-28.5	-24.7	3.8
	Evi-1	13.6	6.2	-7.4
	GATA	10.2	10.8	0.6
	HMG-IY	3.1	-4.9	-8.0
	Hlx1	7.5	8.0	0.5
	Hoxb4	6.2	6.3	0.1
	Msx-1	11.8	12.2	0.4
	Ncx	9.5	11.0	1.5
	Nkx6-1	3.6	3.8	0.2
	PLZF	-2.6	5.9	8.5
	Pax-4	3.0	4.5	1.5
	Pax-6	8.0	8.4	0.4
	Pou2f2	11.6	12.5	0.9
	Pou3f2	10.3	9.4	-0.9
	Pou3f4	2.4	3.2	0.8
	Pou6f1	9.6	10.4	0.8
rs679620 (ref:T, alt:C)	p300	16.4	14.3	-2.0
rs243865 (ref:C, alt:T)	Myf	7.6	-4.3	-11.9
rs3918242 (ref:C, alt:T)	Ahr:Arnt	11.3	-0.6	-11.9
	E2F	6.2	10.2	4.0
	HIF1	7.2	-4.7	-11.9
	Myc	10.4	-0.9	-11.3
rs3918249 (ref:T, alt:C)	Arid3a	11.0	10.3	-0.7
	Hmx	9.7	11.6	1.9
	Hoxb8	9.5	12.4	2.9
	Pax-5	12.7	8.8	-3.9

rs17576 (ref:A, alt:G)	Pax-4	11.6	9.5	-2.1
rs3787268 (ref:G, alt:A)	HDAC2	11.6	13.2	1.6
	Mef2	-5.5	6.5	12.0
	Pou1f1	9.9	11.7	1.8
	Sox	5.9	14.2	8.3
	Zfp105	9.6	13.1	3.5
	p300	11.8	12.7	0.9
rs2250889 (ref:G, alt:C)	NRSF	6.5	11.1	4.6

Note: ΔLOD = LOD (alt) - LOD (ref). A negative value indicates the increased affinity of this

on affinity of the DNA regulatory motifs

increases (+) or decreases (-) affinity motifs
-
-
+
-
+
+
-
-
+
-
+
-
+
+
+
+
+
+
+
+
+
-
+
+
-
-
-
+
-
-
-
+
+
-

-
+
+
+
+
+
+
+

motif for the reference allele, while a positive value suggests the respective increase for the alternativ[illegible]

e allele.
