# Supplementary material for: Matrix Metalloproteinase Gene Polymorphisms Are Associated with Breast Cancer in the Caucasian Women of Russia
Source: Int J Mol Sci. 2022 Oct 20;23(20):12638. doi: 10.3390/ijms232012638 (PMC9604098; doi:10.3390/ijms232012638)
Supplement: Supplementary file 1 [file ijms-23-12638-s001.zip › Suppl table s6.pdf]

Supplementary table S6 cis-eQTL values of the SNPs in high LD (r2≥0.80) with BC-associated SNPs of the *M*
(according to Blood eQTL browser (http://genenetwork.nl/bloodeqtlbrowser/), FDR≤0.05)

Chr	Lead SNP	Position lead SNP(hg38)	Proxy SNP	Position proxy SNP (hg38)	Distance (bp) between proxy and lead SNP
16	rs243865	55477894	rs243866	55477625	-269
16	rs243865	55477894	rs243864	55478410	516
20	rs3918242	46007337	rs1888235	45995328	12009
20	rs3918242	46007337	rs6065912	46003728	3609
20	rs3918242	46007337	rs3918241	46007096	241
20	rs3918242	46007337	rs2274755	46011053	-3716
20	rs3918242	46007337	rs2236416	46011936	-4599
20	rs3918242	46007337	rs3918261	46014953	-7616
20	rs3918242	46007337	rs3918270	46016700	-9363
20	rs3787268	46013092	rs6073983	46001252	11840
20	rs3787268	46013092	rs6073991	46027473	-14381
20	rs17577	46014472	rs1888235	45995328	19144
20	rs17577	46014472	rs6065912	46003728	10744
20	rs17577	46014472	rs3918241	46007096	7376
20	rs17577	46014472	rs2274755	46011053	3419
20	rs17577	46014472	rs2236416	46011936	2536
20	rs17577	46014472	rs3918261	46014953	-481
20	rs17577	46014472	rs3918270	44645339	1369133

MP genes in blood

r2 between lead and proxy	D between lead and proxy	Gene Symbol	Z-score	p	FDR
0.99	1	*AYTL1*	-16.6	6.96E-62	0
0.99	1	*AYTL1*	-17.55	6.03E-69	0
0.95	0.98	*AL162458.10-3,MMP9*	11.09	1.15E-32	0
		DNTTIP1	-4.7	2.61E-06	0
0.86	0.98	*AL162458.10-3,MMP9*	12.83	2.59E-32	0
		DNTTIP1	-4.85	1.26E-06	0
1	1	*AL162458.10-3,MMP9*	12.33	6.62E-35	0
		DNTTIP1	-4.6	4.21E-06	0
0.99	1	*AL162458.10-3,MMP9*	12.57	3.19E-36	0
		DNTTIP1	-4.18	0.000029	0.01
0.99	1	*AL162458.10-3,MMP9*	12.59	2.33E-36	0
		DNTTIP1	-4.13	0.000035	0.02
0.98	1	*AL162458.10-3,MMP9*	12.62	1.64E-36	0
		DNTTIP1	-4.18	0.000029	0.01
0.96	1	*AL162458.10-3,MMP9*	12.62	1.64E-36	0
		DNTTIP1	-4.18	0.000028	0.01
0.89	0.98	*AL162458.10-3,MMP9*	-8.59	8.68E-18	0
0.95	0.98	*AL162458.10-3,MMP9*	-8.85	8.99E-19	0
0.94	0.98	*AL162458.10-3,MMP9*	11.09	1.15E-32	0
		DNTTIP1	-4.7	2.61E-06	0
0.84	0.98	*AL162458.10-3,MMP9*	11.83	2.59E-32	0
		DNTTIP1	-4.85	1.26E-06	0
0.98	1	*AL162458.10-3,MMP9*	12.33	6.62E-35	0
		DNTTIP1	-4.6	4.21E-06	0
0.99	1	*AL162458.10-3,MMP9*	12.57	3.19E-36	0
		DNTTIP1	-4.18	0.000029	0.01
0.99	1	*AL162458.10-3,MMP9*	12.59	2.33E-36	0
		DNTTIP1	-4.13	0.000035	0.02
1	1	*AL162458.10-3,MMP9*	12.62	1.64E-36	0
		DNTTIP1	-4.18	0.000029	0.01
0.98	1	*AL162458.10-3,MMP9*	12.62	1.64E-36	0
		DNTTIP1	-4.18	0.000028	0.01
