# Supplementary material for: Matrix Metalloproteinase Gene Polymorphisms Are Associated with Breast Cancer in the Caucasian Women of Russia
Source: Int J Mol Sci. 2022 Oct 20;23(20):12638. doi: 10.3390/ijms232012638 (PMC9604098; doi:10.3390/ijms232012638)
Supplement: Supplementary file 1 [file ijms-23-12638-s001.zip › Suppl table s7.pdf]

Supplementary table S7 eQTL values of the BC-associated SNPs of the MMP genes
(according to Genotype-Tissue Expression (GTEx) (http://www.gtexportal.org/)) ( p <8.0E5, FDR≤0.05)

| Chr | SNP | Gene Symbol | Variant Id | P-Value |
|---|---|---|---|---|
| 11 | rs1940475 | *MMP27* | chr11_102722517_T_C_b38 | 0.0000000000022 |
| | | *MMP27* | chr11_102722517_T_C_b38 | 0.000000000017 |
| | | *MMP27* | chr11_102722517_T_C_b38 | 0.000000006 |
| | | *RP11-817J15.3* | chr11_102722517_T_C_b38 | 0.000000055 |
| | | *MMP27* | chr11_102722517_T_C_b38 | 0.000000068 |
| | | *RP11-817J15.3* | chr11_102722517_T_C_b38 | 0.0000038 |
| | | *MMP27* | chr11_102722517_T_C_b38 | 0.000011 |
| | | | | |
| 11 | rs1799750 | *MMP1* | chr11_102799764_TC_T_b38 | 9.6E-84 |
| | | *MMP1* | chr11_102799764_TC_T_b38 | 1.3E-25 |
| | | *MMP1* | chr11_102799764_TC_T_b38 | 1.9E-25 |
| | | *MMP1* | chr11_102799764_TC_T_b38 | 5.8E-23 |
| | | *MMP1* | chr11_102799764_TC_T_b38 | .000000000000000001 |
| | | *MMP1* | chr11_102799764_TC_T_b38 | 0.0000000000000066 |
| | | *MMP1* | chr11_102799764_TC_T_b38 | 0.000000000022 |
| | | *MMP1* | chr11_102799764_TC_T_b38 | 0.000000013 |
| | | *MMP1* | chr11_102799764_TC_T_b38 | 0.000000015 |
| | | *MMP1* | chr11_102799764_TC_T_b38 | 0.000000024 |
| | | *MMP1* | chr11_102799764_TC_T_b38 | 0.00000038 |
| | | *MMP10* | chr11_102799764_TC_T_b38 | 0.0000025 |
| | | *MMP1* | chr11_102799764_TC_T_b38 | 0.0000028 |
| | | *MMP1* | chr11_102799764_TC_T_b38 | 0.0000075 |
| | | *WTAPP1* | chr11_102799764_TC_T_b38 | 0.00004 |
| | | | | |
| 11 | rs679620 | *WTAPP1* | 11_102713620_T_C_b37 | 0.0000000045 |
| | | *MMP1* | 11_102713620_T_C_b37 | 0.0000000047 |
| | | *MMP1* | 11_102713620_T_C_b37 | 0.000023 |
| | | | | |
| 16 | rs243865 | *RP11-212I21.2* | chr16_55477894_C_T_b38 | 0.000061 |
| | | | | |
| 20 | rs3918242 | *SLC12A5* | chr20_46007337_C_T_b38 | 4.6E-26 |
| | | *SLC12A5* | chr20_46007337_C_T_b38 | 2.7E-24 |
| | | *SLC12A5* | chr20_46007337_C_T_b38 | 8.5E-23 |
| | | *SLC12A5* | chr20_46007337_C_T_b38 | 0.0000000000002 |
| | | *SLC12A5* | chr20_46007337_C_T_b38 | 0.00000000003 |
| | | *SLC12A5* | chr20_46007337_C_T_b38 | 0.0000000002 |
| | | *SLC12A5* | chr20_46007337_C_T_b38 | 0.00000000028 |
| | | *SLC12A5* | chr20_46007337_C_T_b38 | 0.0000000015 |
| | | *SLC12A5* | chr20_46007337_C_T_b38 | 0.00000003 |
| | | *SLC12A5* | chr20_46007337_C_T_b38 | 0.000000049 |
| | | *SLC12A5* | chr20_46007337_C_T_b38 | 0.00000016 |
| | | *PLTP* | chr20_46007337_C_T_b38 | 0.00000026 |

| | | | | |
|---|---|---|---|---|
| | | *SLC12A5* | chr20_46007337_C_T_b38 | 0.00000079 |
| | | *PLTP* | chr20_46007337_C_T_b38 | 0.0000019 |
| | | *SLC12A5* | chr20_46007337_C_T_b38 | 0.0000025 |
| | | *SLC12A5* | chr20_46007337_C_T_b38 | 0.0000035 |
| | | *RPL13P2* | chr20_46007337_C_T_b38 | 0.0000052 |
| | | *MMP9* | chr20_46007337_C_T_b38 | 0.0000087 |
| | | *SNX21* | chr20_46007337_C_T_b38 | 0.000011 |
| | | *SLC12A5* | chr20_46007337_C_T_b38 | 0.000019 |
| | | *WFDC3* | chr20_46007337_C_T_b38 | 0.000028 |
| | | *PLTP* | chr20_46007337_C_T_b38 | 0.00003 |
| | | *PLTP* | chr20_46007337_C_T_b38 | 0.000033 |
| | | *SLC12A5* | chr20_46007337_C_T_b38 | 0.000035 |
| | | *DNTTIP1* | chr20_46007337_C_T_b38 | 0.000053 |
| | | *MMP9* | chr20_46007337_C_T_b38 | 0.000064 |
| | | *PLTP* | chr20_46007337_C_T_b38 | 0.000075 |
| | | *WFDC3* | chr20_46007337_C_T_b38 | 0.000085 |
| | | *SYS1* | chr20_46007337_C_T_b38 | 0.000096 |
| | | *PLTP* | chr20_46007337_C_T_b38 | 0.00011 |
| | | *WFDC3* | chr20_46007337_C_T_b38 | 0.00015 |
| | | *PLTP* | chr20_46007337_C_T_b38 | 0.00016 |
| | | | | |
| 20 | rs3918249 | *SLC12A5* | chr20_46009497_T_C_b38 | 0.000000000000055 |
| | | *PLTP* | chr20_46009497_T_C_b38 | 0.0000000000015 |
| | | *PLTP* | chr20_46009497_T_C_b38 | 0.000000000013 |
| | | *NEURL2* | chr20_46009497_T_C_b38 | 0.000000000043 |
| | | *MMP9* | chr20_46009497_T_C_b38 | 0.00000000065 |
| | | *PLTP* | chr20_46009497_T_C_b38 | 0.0000000012 |
| | | *RP3-337O18.9* | chr20_46009497_T_C_b38 | 0.000000012 |
| | | *NEURL2* | chr20_46009497_T_C_b38 | 0.000000014 |
| | | *ZSWIM1* | chr20_46009497_T_C_b38 | 0.000000018 |
| | | *ZNF335* | chr20_46009497_T_C_b38 | 0.000000026 |
| | | *PLTP* | chr20_46009497_T_C_b38 | 0.000000053 |
| | | *PLTP* | chr20_46009497_T_C_b38 | 0.00000015 |
| | | *PLTP* | chr20_46009497_T_C_b38 | 0.00000016 |
| | | *RP3-337O18.9* | chr20_46009497_T_C_b38 | 0.00000023 |
| | | *ZNF335* | chr20_46009497_T_C_b38 | 0.00000028 |
| | | *PLTP* | chr20_46009497_T_C_b38 | 0.00000029 |
| | | *MMP9* | chr20_46009497_T_C_b38 | 0.00000034 |
| | | *RP11-465L10.10* | chr20_46009497_T_C_b38 | 0.00000041 |
| | | *SPATA25* | chr20_46009497_T_C_b38 | 0.00000056 |
| | | *PLTP* | chr20_46009497_T_C_b38 | 0.00000057 |
| | | *RP3-337O18.9* | chr20_46009497_T_C_b38 | 0.00000063 |
| | | *NEURL2* | chr20_46009497_T_C_b38 | 0.0000011 |
| | | *PLTP* | chr20_46009497_T_C_b38 | 0.0000016 |
| | | *PLTP* | chr20_46009497_T_C_b38 | 0.0000023 |
| | | *RP3-337O18.9* | chr20_46009497_T_C_b38 | 0.0000035 |
| | | *NEURL2* | chr20_46009497_T_C_b38 | 0.0000036 |

| | | | |
|---|---|---|---|
| | *PCIF1* | chr20_46009497_T_C_b38 | 0.0000056 |
| | *PLTP* | chr20_46009497_T_C_b38 | 0.000012 |
| | *CD40* | chr20_46009497_T_C_b38 | 0.000013 |
| | *PLTP* | chr20_46009497_T_C_b38 | 0.000016 |
| | *CD40* | chr20_46009497_T_C_b38 | 0.000026 |
| | *DNTTIP1* | chr20_46009497_T_C_b38 | 0.000029 |
| | *MMP9* | chr20_46009497_T_C_b38 | 0.000031 |
| | *DNTTIP1* | chr20_46009497_T_C_b38 | 0.000041 |
| | *NEURL2* | chr20_46009497_T_C_b38 | 0.000049 |
| | *PLTP* | chr20_46009497_T_C_b38 | 0.000049 |
| | *CD40* | chr20_46009497_T_C_b38 | 0.000057 |
| | *PLTP* | chr20_46009497_T_C_b38 | 0.000062 |
| | *WFDC3* | chr20_46009497_T_C_b38 | 0.000062 |
| | *RP3-337O18.9* | chr20_46009497_T_C_b38 | 0.000083 |
| | *DNTTIP1* | chr20_46009497_T_C_b38 | 0.00012 |
| | *SNX21* | chr20_46009497_T_C_b38 | 0.00014 |
| | *WFDC10B* | chr20_46009497_T_C_b38 | 0.00019 |
| | *SPATA25* | chr20_46009497_T_C_b38 | 0.0002 |
| 20 rs17576 | *PLTP* | chr20_46011586_A_G_b38 | 0.000000000000015 |
| | *SLC12A5* | chr20_46011586_A_G_b38 | 0.0000000000073 |
| | *NEURL2* | chr20_46011586_A_G_b38 | 0.000000000073 |
| | *PLTP* | chr20_46011586_A_G_b38 | 0.00000000019 |
| | *PLTP* | chr20_46011586_A_G_b38 | 0.00000000038 |
| | *RP3-337O18.9* | chr20_46011586_A_G_b38 | 0.0000000011 |
| | *NEURL2* | chr20_46011586_A_G_b38 | 0.0000000022 |
| | *PLTP* | chr20_46011586_A_G_b38 | 0.0000000031 |
| | *ZSWIM1* | chr20_46011586_A_G_b38 | 0.0000000034 |
| | *PLTP* | chr20_46011586_A_G_b38 | 0.0000000038 |
| | *PLTP* | chr20_46011586_A_G_b38 | 0.0000000089 |
| | *MMP9* | chr20_46011586_A_G_b38 | 0.000000019 |
| | *RP3-337O18.9* | chr20_46011586_A_G_b38 | 0.000000047 |
| | *PLTP* | chr20_46011586_A_G_b38 | 0.00000005 |
| | *RP3-337O18.9* | chr20_46011586_A_G_b38 | 0.000000069 |
| | *SPATA25* | chr20_46011586_A_G_b38 | 0.000000082 |
| | *NEURL2* | chr20_46011586_A_G_b38 | 0.000000092 |
| | *PLTP* | chr20_46011586_A_G_b38 | 0.0000001 |
| | *PLTP* | chr20_46011586_A_G_b38 | 0.00000013 |
| | *NEURL2* | chr20_46011586_A_G_b38 | 0.00000021 |
| | *RP11-465L10.10* | chr20_46011586_A_G_b38 | 0.00000041 |
| | *PLTP* | chr20_46011586_A_G_b38 | 0.00000056 |
| | *ZNF335* | chr20_46011586_A_G_b38 | 0.00000077 |
| | *PLTP* | chr20_46011586_A_G_b38 | 0.0000019 |
| | *PLTP* | chr20_46011586_A_G_b38 | 0.0000025 |
| | *PLTP* | chr20_46011586_A_G_b38 | 0.0000027 |
| | *PLTP* | chr20_46011586_A_G_b38 | 0.000003 |
| | *RP3-337O18.9* | chr20_46011586_A_G_b38 | 0.0000058 |

| | | | |
|---|---|---|---|
| | *ZNF335* | chr20_46011586_A_G_b38 | 0.0000062 |
| | *PCIF1* | chr20_46011586_A_G_b38 | 0.0000076 |
| | *PLTP* | chr20_46011586_A_G_b38 | 0.0000089 |
| | *NEURL2* | chr20_46011586_A_G_b38 | 0.000013 |
| | *RP3-337O18.9* | chr20_46011586_A_G_b38 | 0.000018 |
| | *NEURL2* | chr20_46011586_A_G_b38 | 0.000018 |
| | *SLC12A5* | chr20_46011586_A_G_b38 | 0.000023 |
| | *NEURL2* | chr20_46011586_A_G_b38 | 0.000035 |
| | *MMP9* | chr20_46011586_A_G_b38 | 0.000036 |
| | *CD40* | chr20_46011586_A_G_b38 | 0.000041 |
| | *NEURL2* | chr20_46011586_A_G_b38 | 0.000052 |
| | *CD40* | chr20_46011586_A_G_b38 | 0.000069 |
| | *RP3-337O18.9* | chr20_46011586_A_G_b38 | 0.000074 |
| | *WFDC3* | chr20_46011586_A_G_b38 | 0.000099 |
| | *CD40* | chr20_46011586_A_G_b38 | 0.000099 |
| | *DNTTIP1* | chr20_46011586_A_G_b38 | 0.00012 |
| | *RP3-337O18.9* | chr20_46011586_A_G_b38 | 0.00013 |
| | *SLC12A5* | chr20_46011586_A_G_b38 | 0.00014 |
| | | | |
| 20 rs3787268 | *NEURL2* | chr20_46013092_G_A_b38 | 0.000000000059 |
| | *NEURL2* | chr20_46013092_G_A_b38 | 0.000000005 |
| | *RP3-337O18.9* | chr20_46013092_G_A_b38 | 0.000000011 |
| | *SLC12A5* | chr20_46013092_G_A_b38 | 0.00000012 |
| | *SPATA25* | chr20_46013092_G_A_b38 | 0.00000015 |
| | *RP3-337O18.9* | chr20_46013092_G_A_b38 | 0.00000018 |
| | *ZNF335* | chr20_46013092_G_A_b38 | 0.00000045 |
| | *PLTP* | chr20_46013092_G_A_b38 | 0.0000005 |
| | *ZSWIM1* | chr20_46013092_G_A_b38 | 0.00000066 |
| | *PLTP* | chr20_46013092_G_A_b38 | 0.000002 |
| | *PLTP* | chr20_46013092_G_A_b38 | 0.0000022 |
| | *RP3-337O18.9* | chr20_46013092_G_A_b38 | 0.0000035 |
| | *RP3-337O18.9* | chr20_46013092_G_A_b38 | 0.000014 |
| | *NEURL2* | chr20_46013092_G_A_b38 | 0.000017 |
| | *SLC12A5* | chr20_46013092_G_A_b38 | 0.00002 |
| | *PLTP* | chr20_46013092_G_A_b38 | 0.000021 |
| | *CD40* | chr20_46013092_G_A_b38 | 0.000022 |
| | *NEURL2* | chr20_46013092_G_A_b38 | 0.00003 |
| | *PLTP* | chr20_46013092_G_A_b38 | 0.000036 |
| | *PLTP* | chr20_46013092_G_A_b38 | 0.000075 |
| | *PLTP* | chr20_46013092_G_A_b38 | 0.000076 |
| | *CD40* | chr20_46013092_G_A_b38 | 0.000098 |
| | *ZNF335* | chr20_46013092_G_A_b38 | 0.00015 |
| | | | |
| 20 rs2250889 | *PLTP* | chr20_46013767_G_C_b38 | 0.00000000000075 |
| | *PLTP* | chr20_46013767_G_C_b38 | 0.000000000062 |
| | *PLTP* | chr20_46013767_G_C_b38 | 0.00000000068 |
| | *PCIF1* | chr20_46013767_G_C_b38 | 0.000065 |

| | | | |
|---|---|---|---|
| | *NEURL2* | chr20_46013767_G_C_b38 | 0.00012 |
| 20 rs17577 | *SLC12A5* | chr20_46014472_G_A_b38 | 2.1E-30 |
| | *SLC12A5* | chr20_46014472_G_A_b38 | 5.9E-30 |
| | *SLC12A5* | chr20_46014472_G_A_b38 | 8.00E-24 |
| | *SLC12A5* | chr20_46014472_G_A_b38 | 0.00000000000000042 |
| | *SLC12A5* | chr20_46014472_G_A_b38 | 0.0000000000057 |
| | *SLC12A5* | chr20_46014472_G_A_b38 | 0.000000000019 |
| | *SLC12A5* | chr20_46014472_G_A_b38 | 0.000000000059 |
| | *SLC12A5* | chr20_46014472_G_A_b38 | 0.00000000018 |
| | *SLC12A5* | chr20_46014472_G_A_b38 | 0.0000000034 |
| | *SLC12A5* | chr20_46014472_G_A_b38 | 0.000000031 |
| | *PLTP* | chr20_46014472_G_A_b38 | 0.00000026 |
| | *SLC12A5* | chr20_46014472_G_A_b38 | 0.00000033 |
| | *SLC12A5* | chr20_46014472_G_A_b38 | 0.00000072 |
| | *SLC12A5* | chr20_46014472_G_A_b38 | 0.0000011 |
| | *SLC12A5* | chr20_46014472_G_A_b38 | 0.0000062 |
| | *SLC12A5* | chr20_46014472_G_A_b38 | 0.0000064 |
| | *PLTP* | chr20_46014472_G_A_b38 | 0.000011 |
| | *SLC12A5* | chr20_46014472_G_A_b38 | 0.000011 |
| | *SYS1* | chr20_46014472_G_A_b38 | 0.000034 |
| | *SNX21* | chr20_46014472_G_A_b38 | 0.000046 |
| | *RPL13P2* | chr20_46014472_G_A_b38 | 0.000057 |
| | *DNTTIP1* | chr20_46014472_G_A_b38 | 0.00006 |
| | *PLTP* | chr20_46014472_G_A_b38 | 0.000062 |
| | *MMP9* | chr20_46014472_G_A_b38 | 0.00008 |
| | *RP11-465L10.10* | chr20_46014472_G_A_b38 | 0.00013 |

| NES | Tissue |
|---|---|
| 0.24 | Whole Blood |
| -0.17 | Skin - Sun Exposed (Lower leg) |
| -0.19 | Skin - Not Sun Exposed (Suprapubic) |
| 0.17 | Whole Blood |
| -0.15 | Adipose - Subcutaneous |
| -0.44 | Liver |
| -0.17 | Muscle - Skeletal |
| | |
| -0.66 | Cells - Cultured fibroblasts |
| -0.52 | Thyroid |
| -0.42 | Lung |
| -0.58 | Heart - Atrial Appendage |
| -0.45 | Adipose - Visceral (Omentum) |
| -0.46 | Heart - Left Ventricle |
| -0.36 | Nerve - Tibial |
| -0.32 | Esophagus - Muscularis |
| -0.35 | Artery - Aorta |
| -0.28 | Adipose - Subcutaneous |
| -0.22 | Artery - Tibial |
| -0.19 | Lung |
| -0.3 | Esophagus - Gastroesophageal Junction |
| -0.28 | Breast - Mammary Tissue |
| -0.15 | Testis |
| | |
| 0.28 | Testis |
| 0.31 | Cells - Transformed fibroblasts |
| 0.3 | Heart - Left Ventricle |
| | |
| 0.27 | Artery - Aorta |
| | |
| 0.64 | Lung |
| 0.72 | Adipose - Subcutaneous |
| 0.75 | Adipose - Visceral (Omentum) |
| 0.7 | Breast - Mammary Tissue |
| 0.67 | Artery - Aorta |
| 0.79 | Artery - Coronary |
| 0.48 | Thyroid |
| 0.69 | Spleen |
| 0.43 | Nerve - Tibial |
| 0.41 | Skin - Sun Exposed (Lower leg) |
| 0.46 | Esophagus - Muscularis |
| -0.31 | Heart - Atrial Appendage |

| | |
|---|---|
| 0.38 | Artery - Tibial |
| -0.24 | Lung |
| -0.49 | Adrenal Gland |
| 0.41 | Skin - Not Sun Exposed (Suprapubic) |
| -0.47 | Testis |
| -0.25 | Cells - Cultured fibroblasts |
| 0.17 | Muscle - Skeletal |
| 0.63 | Small Intestine - Terminal Ileum |
| -0.31 | Thyroid |
| -0.19 | Esophagus - Muscularis |
| -0.31 | Pituitary |
| 0.38 | Colon - Transverse |
| -0.17 | Artery - Tibial |
| 0.35 | Testis |
| -0.16 | Artery - Tibial |
| -0.27 | Skin - Sun Exposed (Lower leg) |
| -0.12 | Whole Blood |
| -0.17 | Thyroid |
| -0.25 | Artery - Tibial |
| -0.18 | Nerve - Tibial |
| | |
| -0.52 | Adrenal Gland |
| -0.25 | Lung |
| -0.28 | Colon - Sigmoid |
| -0.26 | Adipose - Subcutaneous |
| -0.23 | Cells - Cultured fibroblasts |
| -0.17 | Adipose - Subcutaneous |
| -0.21 | Adipose - Subcutaneous |
| -0.24 | Adipose - Visceral (Omentum) |
| -0.24 | Adipose - Visceral (Omentum) |
| 0.12 | Skin - Not Sun Exposed (Suprapubic) |
| -0.18 | Nerve - Tibial |
| -0.15 | Artery - Tibial |
| -0.15 | Adipose - Visceral (Omentum) |
| -0.22 | Lung |
| 0.098 | Whole Blood |
| -0.16 | Thyroid |
| 0.26 | Skin - Not Sun Exposed (Suprapubic) |
| -0.41 | Adrenal Gland |
| -0.25 | Adipose - Visceral (Omentum) |
| -0.21 | Heart - Left Ventricle |
| -0.22 | Adipose - Visceral (Omentum) |
| -0.22 | Lung |
| -0.17 | Colon - Transverse |
| -0.12 | Muscle - Skeletal |
| -0.28 | Colon - Sigmoid |
| -0.2 | Thyroid |

| | |
|---|---|
| 0.31 | Adrenal Gland |
| -0.22 | Pituitary |
| 0.19 | Adipose - Visceral (Omentum) |
| -0.19 | Heart - Atrial Appendage |
| 0.15 | Skin - Sun Exposed (Lower leg) |
| -0.14 | Spleen |
| 0.26 | Testis |
| -0.12 | Artery - Tibial |
| -0.22 | Colon - Transverse |
| -0.15 | Artery - Aorta |
| 0.15 | Adipose - Subcutaneous |
| -0.13 | Esophagus - Muscularis |
| -0.18 | Artery - Tibial |
| -0.24 | Heart - Atrial Appendage |
| -0.049 | Whole Blood |
| 0.1 | Muscle - Skeletal |
| 0.14 | Skin - Sun Exposed (Lower leg) |
| -0.19 | Nerve - Tibial |
| | |
| -0.27 | Lung |
| -0.49 | Adrenal Gland |
| -0.26 | Adipose - Subcutaneous |
| -0.28 | Colon - Sigmoid |
| -0.17 | Adipose - Subcutaneous |
| -0.23 | Adipose - Subcutaneous |
| -0.25 | Adipose - Visceral (Omentum) |
| -0.2 | Nerve - Tibial |
| -0.26 | Adipose - Visceral (Omentum) |
| -0.17 | Adipose - Visceral (Omentum) |
| -0.16 | Artery - Tibial |
| -0.22 | Cells - Cultured fibroblasts |
| -0.24 | Adipose - Visceral (Omentum) |
| -0.17 | Thyroid |
| -0.23 | Lung |
| -0.26 | Adipose - Visceral (Omentum) |
| -0.24 | Lung |
| -0.22 | Heart - Left Ventricle |
| -0.13 | Muscle - Skeletal |
| -0.22 | Thyroid |
| -0.42 | Adrenal Gland |
| -0.18 | Colon - Transverse |
| 0.11 | Skin - Not Sun Exposed (Suprapubic) |
| -0.18 | Artery - Aorta |
| -0.21 | Heart - Atrial Appendage |
| -0.15 | Esophagus - Muscularis |
| -0.25 | Adrenal Gland |
| -0.28 | Colon - Sigmoid |

| | |
|---|---|
| 0.088 | Whole Blood |
| 0.31 | Adrenal Gland |
| -0.22 | Pituitary |
| -0.23 | Colon - Transverse |
| -0.26 | Heart - Atrial Appendage |
| -0.25 | Esophagus - Gastroesophageal Junction |
| 0.19 | Lung |
| -0.22 | Artery - Aorta |
| 0.21 | Skin - Not Sun Exposed (Suprapubic) |
| 0.15 | Skin - Sun Exposed (Lower leg) |
| -0.18 | Skin - Sun Exposed (Lower leg) |
| 0.17 | Adipose - Visceral (Omentum) |
| -0.23 | Esophagus - Gastroesophageal Junction |
| -0.18 | Artery - Tibial |
| 0.14 | Adipose - Subcutaneous |
| -0.12 | Artery - Tibial |
| -0.18 | Nerve - Tibial |
| 0.2 | Adipose - Subcutaneous |
| | |
| -0.31 | Adipose - Subcutaneous |
| -0.32 | Adipose - Visceral (Omentum) |
| -0.32 | Adipose - Visceral (Omentum) |
| -0.4 | Adipose - Visceral (Omentum) |
| -0.33 | Adipose - Visceral (Omentum) |
| -0.23 | Adipose - Subcutaneous |
| 0.12 | Whole Blood |
| -0.22 | Lung |
| -0.28 | Adipose - Visceral (Omentum) |
| -0.2 | Nerve - Tibial |
| -0.31 | Adrenal Gland |
| -0.24 | Lung |
| -0.5 | Vagina |
| -0.23 | Esophagus - Muscularis |
| -0.26 | Adipose - Subcutaneous |
| -0.14 | Adipose - Subcutaneous |
| 0.18 | Adipose - Subcutaneous |
| -0.22 | Lung |
| -0.14 | Brain - Frontal Cortex (BA9) |
| -0.22 | Colon - Sigmoid |
| -0.17 | Artery - Aorta |
| 0.17 | Skin - Sun Exposed (Lower leg) |
| 0.1 | Skin - Not Sun Exposed (Suprapubic) |
| | |
| 0.49 | Esophagus - Mucosa |
| 0.42 | Skin - Sun Exposed (Lower leg) |
| 0.45 | Skin - Not Sun Exposed (Suprapubic) |
| -0.12 | Whole Blood |

| | |
|---|---|
| 0.35 | Nerve - Tibial |
| | |
| 0.69 | Lung |
| 0.79 | Adipose - Subcutaneous |
| 0.76 | Adipose - Visceral (Omentum) |
| 0.75 | Breast - Mammary Tissue |
| 0.75 | Spleen |
| 0.68 | Artery - Aorta |
| 0.79 | Artery - Coronary |
| 0.48 | Thyroid |
| 0.46 | Nerve - Tibial |
| 0.41 | Skin - Sun Exposed (Lower leg) |
| -0.31 | Heart - Atrial Appendage |
| 0.38 | Artery - Tibial |
| 0.45 | Colon - Transverse |
| 0.43 | Esophagus - Muscularis |
| 0.39 | Skin - Not Sun Exposed (Suprapubic) |
| 0.86 | Uterus |
| -0.23 | Lung |
| 0.63 | Small Intestine - Terminal Ileum |
| -0.13 | Whole Blood |
| 0.15 | Muscle - Skeletal |
| -0.42 | Testis |
| -0.17 | Artery - Tibial |
| -0.18 | Esophagus - Muscularis |
| 0.35 | Testis |
| -0.27 | Skin - Not Sun Exposed (Suprapubic) |
