# Supplementary material for: Matrix Metalloproteinase Gene Polymorphisms Are Associated with Breast Cancer in the Caucasian Women of Russia
Source: Int J Mol Sci. 2022 Oct 20;23(20):12638. doi: 10.3390/ijms232012638 (PMC9604098; doi:10.3390/ijms232012638)
Supplement: Supplementary file 1 [file ijms-23-12638-s001.zip › Suppl table s8.pdf]

Supplementary table S8 Effect of SNPs in high LD (r2≥0.80) with the BC-associated polymorphisms of the *MM*
(according to Genotype-Tissue Expression (GTEx) (http://www.gtexportal.org/)) (p <8.0E5, FDR≤0.05)

Chr	Lead SNP	Position lead SNP(hg38)	Proxy SNP	Position proxy SNP (hg38)	Distance (bp) between proxy and lead SNP
11	rs1940475	102722517	rs2186602	102721214	1303
11	rs1940475	102722517	rs948218	102722311	206
11	rs1940475	102722517	rs1940474	102722322	195
11	rs1940475	102722517	rs7123658	102723248	-731
11	rs1940475	102722517	rs6590983	102723645	-1128

11	rs1940475	102722517	rs1939012	102724404	-1887
11	rs1940475	102722517	rs3765620	102724761	-2244
11	rs1940475	102722517	rs11225395	102725749	-3232
11	rs1940475	102722517	rs33952053	102726340	-3823
11	rs1940475	102722517	rs1939013	102727079	-4562

11	rs1940475	102722517	rs1939014	102727181	-4664
11	rs1940475	102722517	rs3758857	102727628	-5111
11	rs1940475	102722517	rs3758858	102727726	-5209
11	rs1940475	102722517	rs3758859	102727749	-5232
11	rs1940475	102722517	rs6590985	102727924	-5407

11	rs1940475	102722517	rs6590986	102728128	-5611
11	rs1940475	102722517	rs10750653	102729275	-6758
11	rs679620	102827993	rs526215	102807707	20286
11	rs679620	102827993	rs1144396	102808321	19672
11	rs679620	102827993	rs565082	102808927	19066
11	rs679620	102827993	rs502174	102811772	16221
11	rs679620	102827993	rs10791596	102816687	11306
11	rs679620	102827993	rs2097248	102819770	8223

11	rs679620	102827993	rs756544	102820431	7562
11	rs679620	102827993	rs12804136	102820659	7334
11	rs679620	102827993	rs11225434	102820751	7242
11	rs679620	102827993	rs7117013	102821493	6500
11	rs679620	102827993	rs10895364	102822132	5861
11	rs679620	102827993	rs12801529	102822879	5114
11	rs679620	102827993	rs10895365	102823462	4531
11	rs679620	102827993	rs6590993	102825487	2506
11	rs679620	102827993	rs6590994	102825901	2092
11	rs679620	102827993	rs10895366	102826358	1635
11	rs679620	102827993	rs7126392	102826390	1603
11	rs679620	102827993	rs2212637	102826825	1168
11	rs679620	102827993	rs12792871	102827605	388
11	rs679620	102827993	rs1010698	102830290	-2297

11	rs679620	102827993	rs2155013	102831127	-3134
11	rs679620	102827993	rs4754884	102831532	-3539
11	rs679620	102827993	rs635746	102832897	-4904
11	rs679620	102827993	rs633962	102833340	-5347
11	rs679620	102827993	rs666734	102833449	-5456
11	rs679620	102827993	rs595840	102834166	-6173
11	rs679620	102827993	rs639752	102836608	-8615
11	rs679620	102827993	rs575027	102837183	-9190
11	rs679620	102827993	rs520540	102838694	-10701
11	rs679620	102827993	rs591058	102840607	-12614
11	rs679620	102827993	rs602128	102842734	-14741
11	rs679620	102827993	rs679620	102842889	-14896
11	rs679620	102827993	rs678815	102843046	-15053
11	rs679620	102827993	rs617819	102843985	-15992
11	rs679620	102827993	rs632478	102844950	-16957
11	rs679620	102827993	rs645419	102845590	-17597

16	rs243865	55477894	rs243866	55477625	269
20	rs3918242	46007337	rs1888235	45995328	12009
20	rs3918242	46007337	rs6073984	46002014	5323
20	rs3918242	46007337	rs6065912	46003728	3609
20	rs3918242	46007337	rs3918241	46007096	241

20	rs3918242	46007337 rs2274755	46011053	-3716
20	rs3918242	46007337 rs2236416	46011936	-4599
20	rs3918242	46007337 rs17577	46014472	-7135

20	rs3918242	46007337	rs3918261	46014953	-7616
20	rs3918242	46007337	rs13925	46016326	-8989
20	rs3918242	46007337	rs3918270	46016700	-9363
20	rs3918242	46007337	rs3918271	46016954	-9617

20	rs3918242	46007337	rs66553311	46020003	-12666
20	rs3918242	46007337	rs10432735	46021679	-14342
20	rs3918249	46009497	rs6017721	45987758	21739

20	rs3918249	46009497 rs3848721	46005037	4460
20	rs3918249	46009497 rs3848722	46005181	4316

20	rs3918249	46009497	rs4810482	46005911	3586
20	rs3918249	46009497	rs3761158	46006273	3224
20	rs3918249	46009497	rs3761159	46006318	3179

20	rs3918249	46009497	rs6065913	46006464	3033
20	rs3918249	46009497	rs3918240	46006977	2520

20	rs3918249	46009497	rs3918251	46010142	-645
20	rs3918249	46009497	rs17576	46011586	-2089

20	rs3918249	46009497	rs13969	46014194	-4697
20	rs17576	46011586	rs6017721	45987758	23828

20	rs17576	46011586	rs3848721	46005037	6549
20	rs17576	46011586	rs3848722	46005181	6405

20	rs17576	46011586	rs4810482	46005911	5675
20	rs17576	46011586	rs3761158	46006273	5313

20	rs17576	46011586	rs3761159	46006318	5268
20	rs17576	46011586	rs6065913	46006464	5122

20	rs17576	46011586	rs3918240	46006977	4609
20	rs17576	46011586	rs3918249	46009497	2089

20	rs17576	46011586	rs3918251	46010142	1444
20	rs17576	46011586	rs13969	46014194	-2608

20	rs3787268	46013092	rs35610751	45991015	22077
20	rs3787268	46013092	rs6073980	45991065	22027
20	rs3787268	46013092	rs6073983	46001252	11840
20	rs3787268	46013092	rs3761157	46006183	6909

20	rs3787268	46013092	rs3918262	46015131	-2039
20	rs3787268	46013092	rs6073989	46022571	-9479
20	rs3787268	46013092	rs16991010	46023675	-10583
20	rs3787268	46013092	rs6130998	46024480	-11388
20	rs3787268	46013092	rs6130999	46026853	-13761
20	rs3787268	46013092	rs6073991	46027473	-14381
20	rs3787268	46013092	rs13039389	46031067	-17975

20	rs3787268	46013092	rs6131001	46032717	-19625
20	rs2250889	46013767	rs6094239	46024910	-11143
20	rs17577	46014472	rs1888235	45995328	19144
20	rs17577	46014472	rs6073984	46002014	12458
20	rs17577	46014472	rs6065912	46003728	10744

20	rs17577	46014472	rs3918241	46007096	7376
20	rs17577	46014472	rs3918242	46007337	7135
20	rs17577	46014472	rs2274755	46011053	3419

20	rs17577	46014472	rs2236416	46011936	2536
20	rs17577	46014472	rs3918261	46014953	-481
20	rs17577	46014472	rs13925	46016326	-1854
20	rs17577	46014472	rs3918270	46016700	-2228

20	rs17577	46014472	rs3918271	46016954	-2482
20	rs17577	46014472	rs66553311	46020003	-5531
20	rs17577	46014472	rs10432735	46021679	-7207

1P genes on gene expression level

r2 between lead and proxy	D between lead and proxy	Gene Symbol	p	Effect Size (β)	Tissue
0.85	1	*MMP27*	0.000000000000000035	0.29	Whole Bloc
		MMP27	0.0000000000000008	-0.21	Skin - Sun E
		MMP27	0.000000000019	-0.2	Adipose - S
		MMP27	0.000000000091	-0.21	Skin - Not S
		MMP27	0.000000051	-0.22	Muscle - Sk
		RP11-817J15.3	0.0000015	0.16	Whole Bloc
		RP11-817J15.3	0.0000021	-0.47	Liver
		MMP27	0.0000085	0.31	Spleen
		MMP27	0.00005	-0.13	Cells - Cultu
		MMP27	0.000091	-0.17	Adipose - V
0.85	1	*MMP27*	0.000000000000000035	0.29	Whole Bloc
		MMP27	0.0000000000000008	-0.21	Skin - Sun E
		MMP27	0.000000000019	-0.2	Adipose - S
		MMP27	0.000000000091	-0.21	Skin - Not S
		MMP27	0.000000051	-0.22	Muscle - Sk
		RP11-817J15.3	0.0000015	0.16	Whole Bloc
		RP11-817J15.3	0.0000021	-0.47	Liver
		MMP27	0.0000085	0.31	Spleen
		MMP27	0.00005	-0.13	Cells - Cultu
		MMP27	0.000091	-0.17	Adipose - V
1	1	*MMP27*	0.0000000000011	0.24	Whole Bloc
		MMP27	0.000000000014	-0.17	Skin - Sun E
		MMP27	0.000000043	-0.18	Skin - Not S
		RP11-817J15.3	0.000000073	0.17	Whole Bloc
		MMP27	0.00000012	-0.15	Adipose - S
		RP11-817J15.3	0.0000053	-0.43	Liver
		MMP27	0.000007	-0.18	Muscle - Sk
		RP11-817J15.3	0.000046	-0.36	Small Intes
0.85	1	*MMP27*	0.000000000000000035	0.29	Whole Bloc
		MMP27	0.0000000000000008	-0.21	Skin - Sun E
		MMP27	0.000000000019	-0.2	Adipose - S
		MMP27	0.000000000091	-0.21	Skin - Not S
		MMP27	0.000000051	-0.22	Muscle - Sk
		RP11-817J15.3	0.0000015	0.16	Whole Bloc
		RP11-817J15.3	0.0000021	-0.47	Liver
		MMP27	0.0000085	0.31	Spleen
		MMP27	0.00005	-0.13	Cells - Cultu
		MMP27	0.000091	-0.17	Adipose - V
0.85	1	*MMP27*	0.000000000000000035	0.29	Whole Bloc
		MMP27	0.0000000000000008	-0.21	Skin - Sun E
		MMP27	0.000000000019	-0.2	Adipose - S

		MMP27	0.000000000091	-0.21	Skin - Not S
		MMP27	0.000000051	-0.22	Muscle - Sk
		RP11-817J15.3	0.0000015	0.16	Whole Bloo
		RP11-817J15.3	0.0000021	-0.47	Liver
		MMP27	0.0000085	0.31	Spleen
		MMP27	0.00005	-0.13	Cells - Cultu
		MMP27	0.000091	-0.17	Adipose - V
1	1	*MMP27*	0.0000000000013	0.24	Whole Bloo
		MMP27	0.0000000000022	-0.18	Skin - Sun E
		MMP27	0.0000000045	-0.19	Skin - Not S
		MMP27	0.000000071	-0.15	Adipose - S
		RP11-817J15.3	0.000000078	0.17	Whole Bloo
		RP11-817J15.3	0.0000031	-0.44	Liver
		MMP27	0.000011	-0.17	Muscle - Sk
0.85	1	*MMP27*	0.0000000000000000035	0.29	Whole Bloo
		MMP27	0.0000000000000008	-0.21	Skin - Sun E
		MMP27	0.000000000019	-0.2	Adipose - S
		MMP27	0.000000000091	-0.21	Skin - Not S
		MMP27	0.000000051	-0.22	Muscle - Sk
		RP11-817J15.3	0.0000015	0.16	Whole Bloo
		RP11-817J15.3	0.0000021	-0.47	Liver
		MMP27	0.0000085	0.31	Spleen
		MMP27	0.00005	-0.13	Cells - Cultu
		MMP27	0.000091	-0.17	Adipose - V
0.84	1	*MMP27*	0.0000000000000000035	0.29	Whole Bloo
		MMP27	0.0000000000000008	-0.21	Skin - Sun E
		MMP27	0.000000000019	-0.2	Adipose - S
		MMP27	0.000000000091	-0.21	Skin - Not S
		MMP27	0.000000051	-0.22	Muscle - Sk
		RP11-817J15.3	0.0000015	0.16	Whole Bloo
		RP11-817J15.3	0.0000021	-0.47	Liver
		MMP27	0.0000085	0.31	Spleen
		MMP27	0.00005	-0.13	Cells - Cultu
		MMP27	0.000091	-0.17	Adipose - V
0.84	1	*MMP27*	0.0000000000000000035	0.29	Whole Bloo
		MMP27	0.0000000000000008	-0.21	Skin - Sun E
		MMP27	0.000000000019	-0.2	Adipose - S
		MMP27	0.000000000091	-0.21	Skin - Not S
		MMP27	0.000000051	-0.22	Muscle - Sk
		RP11-817J15.3	0.0000015	0.16	Whole Bloo
		RP11-817J15.3	0.0000021	-0.47	Liver
		MMP27	0.0000085	0.31	Spleen
		MMP27	0.00005	-0.13	Cells - Cultu
		MMP27	0.000091	-0.17	Adipose - V
0.84	1	*MMP27*	0.0000000000000000049	0.29	Whole Bloo
		MMP27	0.0000000000000012	-0.21	Skin - Sun E
		MMP27	0.000000000012	-0.2	Adipose - S

		MMP27	0.00000000012	-0.21	Skin - Not S
		MMP27	0.000000052	-0.22	Muscle - Sk
		RP11-817J15.3	0.0000018	0.15	Whole Bloo
		RP11-817J15.3	0.0000035	-0.46	Liver
		MMP27	0.000016	0.3	Spleen
		MMP27	0.000056	-0.13	Cells - Cultu
		MMP27	0.000091	-0.17	Adipose - V
0.84	1	*MMP27*	0.0000000000000000035	0.29	Whole Bloo
		MMP27	0.0000000000000008	-0.21	Skin - Sun E
		MMP27	0.000000000019	-0.2	Adipose - S
		MMP27	0.00000000012	-0.21	Skin - Not S
		MMP27	0.000000051	-0.22	Muscle - Sk
		RP11-817J15.3	0.0000015	0.16	Whole Bloo
		RP11-817J15.3	0.0000035	-0.46	Liver
		MMP27	0.0000085	0.31	Spleen
		MMP27	0.00005	-0.13	Cells - Cultu
		MMP27	0.000091	-0.17	Adipose - V
0.84	1	*MMP27*	0.0000000000000000035	0.29	Whole Bloo
		MMP27	0.0000000000000008	-0.21	Skin - Sun E
		MMP27	0.000000000019	-0.2	Adipose - S
		MMP27	0.00000000012	-0.21	Skin - Not S
		MMP27	0.000000051	-0.22	Muscle - Sk
		RP11-817J15.3	0.0000015	0.16	Whole Bloo
		RP11-817J15.3	0.0000035	-0.46	Liver
		MMP27	0.0000085	0.31	Spleen
		MMP27	0.00005	-0.13	Cells - Cultu
		MMP27	0.000091	-0.17	Adipose - V
0.84	1	*MMP27*	0.0000000000000000035	0.29	Whole Bloo
		MMP27	0.0000000000000008	-0.21	Skin - Sun E
		MMP27	0.000000000019	-0.2	Adipose - S
		MMP27	0.00000000012	-0.21	Skin - Not S
		MMP27	0.000000051	-0.22	Muscle - Sk
		RP11-817J15.3	0.0000015	0.16	Whole Bloo
		RP11-817J15.3	0.0000035	-0.46	Liver
		MMP27	0.0000085	0.31	Spleen
		MMP27	0.00005	-0.13	Cells - Cultu
		MMP27	0.000091	-0.17	Adipose - V
0.99	1	*MMP27*	0.0000000000011	0.24	Whole Bloo
		MMP27	0.000000000014	-0.17	Skin - Sun E
		MMP27	0.000000054	-0.18	Skin - Not S
		RP11-817J15.3	0.000000073	0.17	Whole Bloo
		MMP27	0.00000012	-0.15	Adipose - S
		MMP27	0.000007	-0.18	Muscle - Sk
		RP11-817J15.3	0.0000086	-0.42	Liver
		RP11-817J15.3	0.000046	-0.36	Small Intes
0.84	1	*MMP27*	0.0000000000000000035	0.29	Whole Bloo
		MMP27	0.0000000000000008	-0.21	Skin - Sun E

		MMP27	0.000000000019	-0.2	Adipose - S
		MMP27	0.00000000012	-0.21	Skin - Not S
		MMP27	0.000000051	-0.22	Muscle - Sk
		RP11-817J15.3	0.0000015	0.16	Whole Bloo
		RP11-817J15.3	0.0000035	-0.46	Liver
		MMP27	0.0000085	0.31	Spleen
		MMP27	0.00005	-0.13	Cells - Cultu
		MMP27	0.000091	-0.17	Adipose - V
0.84	1	*MMP27*	0.0000000000000000035	0.29	Whole Bloo
		MMP27	0.0000000000000008	-0.21	Skin - Sun E
		MMP27	0.000000000019	-0.2	Adipose - S
		MMP27	0.00000000012	-0.21	Skin - Not S
		MMP27	0.000000051	-0.22	Muscle - Sk
		RP11-817J15.3	0.0000015	0.16	Whole Bloo
		RP11-817J15.3	0.0000035	-0.46	Liver
		MMP27	0.0000085	0.31	Spleen
		MMP27	0.00005	-0.13	Cells - Cultu
		MMP27	0.000091	-0.17	Adipose - V
0.84	1	*MMP27*	0.0000000000000000018	0.29	Whole Bloo
		MMP27	0.0000000000000023	-0.2	Skin - Sun E
		MMP27	0.000000000015	-0.2	Adipose - S
		MMP27	0.00000000062	-0.2	Skin - Not S
		MMP27	0.000000061	-0.22	Muscle - Sk
		RP11-817J15.3	0.0000026	0.15	Whole Bloo
		RP11-817J15.3	0.000004	-0.45	Liver
		MMP27	0.000007	0.31	Spleen
		MMP27	0.000066	-0.19	Colon - Tra
		MMP27	0.000075	-0.13	Cells - Cultu
		MMP27	0.000098	-0.17	Adipose - V
0.82	-0.92	*WTAPP1*	0.0000000021	-0.28	Testis
		MMP1	0.0000000022	-0.34	Cells - Tran
		MMP1	0.0000088	-0.33	Heart - Left
		MMP1	0.000038	-0.31	Nerve - Tib
0.81	-0.92	*WTAPP1*	0.00000000097	-0.28	Testis
		MMP1	0.0000000016	-0.32	Cells - Tran
		MMP1	0.000004	-0.33	Heart - Left
0.82	-0.92	*MMP1*	0.00000000039	-0.33	Cells - Tran
		WTAPP1	0.00000000075	-0.29	Testis
		MMP1	0.0000016	-0.35	Heart - Left
0.81	-0.91	*MMP1*	0.00000000055	-0.33	Cells - Tran
		WTAPP1	0.00000000075	-0.29	Testis
		MMP1	0.0000032	-0.34	Heart - Left
0.83	-0.93	*MMP1*	0.00000000017	-0.34	Cells - Tran
		WTAPP1	0.0000000024	-0.28	Testis
		MMP1	0.0000029	-0.34	Heart - Left
0.89	-0.96	*WTAPP1*	0.00000000032	-0.3	Testis

		MMP1	0.0000000018	-0.32	Cells - Tran
		MMP1	0.000004	-0.33	Heart - Left
0.9	-0.96	*WTAPP1*	0.00000000018	-0.3	Testis
		MMP1	0.00000000091	-0.33	Cells - Tran
		MMP1	0.0000098	-0.32	Heart - Left
		MMP1	0.000042	-0.24	Artery - Tib
0.91	-0.97	*MMP1*	0.00000000059	-0.33	Cells - Tran
		WTAPP1	0.0000000017	-0.29	Testis
		MMP1	0.0000026	-0.35	Heart - Left
		MMP1	0.000048	-0.24	Artery - Tib
0.9	-0.97	*MMP1*	0.00000000083	-0.33	Cells - Tran
		WTAPP1	0.0000000012	-0.29	Testis
		MMP1	0.0000019	-0.35	Heart - Left
0.91	-0.97	*MMP1*	0.0000000015	-0.32	Cells - Tran
		WTAPP1	0.0000000038	-0.28	Testis
		MMP1	0.0000072	-0.32	Heart - Left
0.91	-0.97	*MMP1*	0.0000000015	-0.32	Cells - Tran
		WTAPP1	0.0000000038	-0.28	Testis
		MMP1	0.000007	-0.32	Heart - Left
0.92	-0.98	*WTAPP1*	0.000000000029	-0.31	Testis
		MMP1	0.00000000064	-0.33	Cells - Tran
		MMP1	0.000002	-0.34	Heart - Left
		MMP1	0.00007	-0.23	Artery - Tib
0.92	-0.98	*WTAPP1*	0.000000000026	-0.31	Testis
		MMP1	0.00000000064	-0.33	Cells - Tran
		MMP1	0.0000021	-0.34	Heart - Left
		MMP1	0.00007	-0.23	Artery - Tib
0.89	-0.96	*WTAPP1*	0.000000000031	-0.32	Testis
		MMP1	0.00000000064	-0.33	Cells - Tran
		MMP1	0.0000021	-0.34	Heart - Left
0.92	-0.98	*WTAPP1*	0.00000000018	-0.3	Testis
		MMP1	0.00000000035	-0.34	Cells - Tran
		MMP1	0.00001	-0.32	Heart - Left
		MMP1	0.000063	-0.23	Artery - Tib
0.96	-0.98	*WTAPP1*	0.0000000057	-0.28	Testis
		MMP1	0.00000004	-0.31	Cells - Tran
		MMP1	0.000029	-0.31	Heart - Left
0.96	-0.98	*WTAPP1*	0.000000000054	-0.31	Testis
		MMP1	0.0000000037	-0.31	Cells - Tran
		MMP1	0.0000019	-0.34	Heart - Left
0.94	-0.97	*WTAPP1*	0.00000000067	-0.29	Testis
		MMP1	0.0000000065	-0.31	Cells - Tran
		MMP1	0.0000036	-0.33	Heart - Left
0.96	-0.98	*WTAPP1*	0.0000000057	-0.28	Testis
		MMP1	0.00000004	-0.31	Cells - Tran
0.98	-1	*WTAPP1*	0.000000000024	-0.31	Testis
		MMP1	0.0000000035	-0.32	Cells - Tran

		MMP1	0.0000029	-0.33	Heart - Left
0.98	-1	*WTAPP1*	0.00000000026	-0.3	Testis
		MMP1	0.0000000036	-0.31	Cells - Tran
		MMP1	0.0000039	-0.33	Heart - Left
0.98	-1	*WTAPP1*	0.000000000064	-0.31	Testis
		MMP1	0.0000000055	-0.31	Cells - Tran
		MMP1	0.0000029	-0.33	Heart - Left
0.99	1	*WTAPP1*	0.00000000084	0.29	Testis
		MMP1	0.0000000035	0.31	Cells - Tran
		MMP1	0.000018	0.31	Heart - Left
0.99	1	*MMP1*	0.0000000065	0.31	Cells - Tran
		WTAPP1	0.000000015	0.27	Testis
		MMP1	0.000029	0.3	Heart - Left
0.99	1	*WTAPP1*	0.00000000084	0.29	Testis
		MMP1	0.0000000035	0.31	Cells - Tran
		MMP1	0.000019	0.31	Heart - Left
0.99	1	*WTAPP1*	0.00000000023	0.29	Testis
		MMP1	0.0000000031	0.32	Cells - Tran
		MMP1	0.000014	0.31	Heart - Left
0.99	1	*MMP1*	0.0000000065	0.31	Cells - Tran
		WTAPP1	0.000000015	0.27	Testis
		MMP1	0.00003	0.3	Heart - Left
0.98	0.99	*WTAPP1*	0.00000000084	0.29	Testis
		MMP1	0.0000000064	0.31	Cells - Tran
		MMP1	0.000013	0.31	Heart - Left
0.98	0.99	*WTAPP1*	0.00000000084	0.29	Testis
		MMP1	0.0000000036	0.31	Cells - Tran
		MMP1	0.000018	0.31	Heart - Left
1	1	*WTAPP1*	0.0000000039	0.28	Testis
		MMP1	0.000000005	0.31	Cells - Tran
		MMP1	0.000023	0.3	Heart - Left
1	1	*MMP1*	0.0000000055	0.31	Cells - Tran
		WTAPP1	0.000000017	0.27	Testis
		MMP1	0.000033	0.3	Heart - Left
1	1	*WTAPP1*	0.0000000045	0.28	Testis
		MMP1	0.0000000047	0.31	Cells - Tran
		MMP1	0.000023	0.3	Heart - Left
1	1	*MMP1*	0.0000000047	0.32	Cells - Tran
		WTAPP1	0.0000000054	0.28	Testis
		MMP1	0.000022	0.3	Heart - Left
0.99	0.99	*WTAPP1*	0.0000000045	0.28	Testis
		MMP1	0.0000000049	0.31	Cells - Tran
		MMP1	0.000023	0.3	Heart - Left
0.99	0.99	*MMP1*	0.000000005	0.31	Cells - Tran
		WTAPP1	0.0000000054	0.28	Testis
		MMP1	0.000023	0.3	Heart - Left
0.99	0.99	*MMP1*	0.0000000048	0.31	Cells - Tran

		WTAPP1	0.0000000054	0.28	Testis
		MMP1	0.000023	0.3	Heart - Left
0.99	1	*RP11-212I21.2*	0.000061	0.27	Artery - Ao
0.95	0.98	*SLC12A5*	0.000034	0.38	Skin - Sun E
		PLTP	0.000031	-0.24	Nerve - Tib
		SLC12A5	0.000022	0.46	Skin - Not S
		SLC12A5	0.00001	0.4	Nerve - Tib
		SLC12A5	0.0000026	0.46	Thyroid
		SLC12A5	0.0000006	0.21	Muscle - Sk
		SLC12A5	0.00000046	0.74	Spleen
		SNX21	0.00000033	0.66	Breast - Ma
		SLC12A5	0.00000015	0.63	Artery - Ao
		SLC12A5	0.00000012	-0.62	Adrenal Gla
		SLC12A5	0.0000000053	0.54	Adipose - S
		SLC12A5	0.00000000000022	0.75	Adipose - V
		SLC12A5	0.0000000000000019	0.58	Lung
0.95	0.98	*SLC12A5*	0.000034	0.38	Skin - Sun E
		PLTP	0.000031	-0.24	Nerve - Tib
		SLC12A5	0.000028	0.46	Skin - Not S
		SLC12A5	0.00001	0.4	Nerve - Tib
		SLC12A5	0.000007	0.44	Thyroid
		SNX21	0.00000025	0.77	Spleen
		SLC12A5	0.00000023	0.67	Breast - Ma
		SLC12A5	0.00000015	0.63	Artery - Ao
		SLC12A5	0.000000098	-0.63	Adrenal Gla
		SLC12A5	0.0000000053	0.54	Adipose - S
		SLC12A5	0.00000000000022	0.75	Adipose - V
		SLC12A5	0.0000000000000019	0.58	Lung
0.86	0.98	*SLC12A5*	0.000000000000033	0.8	Adipose - V
		SLC12A5	0.00000000000026	0.54	Lung
		SLC12A5	0.00000004	0.84	Spleen
		SLC12A5	0.00000012	0.51	Adipose - S
		SLC12A5	0.00000026	0.64	Artery - Ao
		SNX21	0.00000039	0.23	Muscle - Sk
		SLC12A5	0.0000046	-0.57	Adrenal Gla
		SLC12A5	0.000014	0.41	Nerve - Tib
		PLTP	0.000018	-0.26	Nerve - Tib
		PLTP	0.000019	-0.28	Lung
		SLC12A5	0.000021	0.59	Breast - Ma
		SLC12A5	0.000025	0.4	Skin - Sun E
		SLC12A5	0.000028	0.42	Thyroid
		SLC12A5	0.000036	0.47	Skin - Not S
		WFDC3	0.000058	-0.4	Nerve - Tib
1	1	*SLC12A5*	0.0000000000000000059	0.61	Lung

		SLC12A5	0.0000000000000016	0.8	Adipose - V
		SLC12A5	0.00000000002	0.61	Adipose - S
		SLC12A5	0.000000045	0.69	Breast - Ma
		SLC12A5	0.000000082	0.63	Artery - Ao
		SLC12A5	0.00000018	0.78	Spleen
		SNX21	0.00000098	0.21	Muscle - Sk
		SLC12A5	0.0000023	-0.56	Adrenal Gla
		SLC12A5	0.0000024	0.43	Nerve - Tib
		SLC12A5	0.0000024	0.45	Thyroid
		SLC12A5	0.0000035	43466.00	Uterus
		SLC12A5	0.0000054	0.41	Skin - Sun E
		SLC12A5	0.00001	0.48	Skin - Not S
		PLTP	0.000056	-0.24	Nerve - Tib
0.99	1	*SLC12A5*	8.9E-22	0.66	Lung
		SLC12A5	0.0000000000000002	0.81	Adipose - V
		SLC12A5	0.00000000000003	0.68	Adipose - S
		SLC12A5	0.00000000011	0.77	Breast - Ma
		SLC12A5	0.00000000015	0.88	Spleen
		SLC12A5	0.000000023	0.67	Artery - Ao
		SLC12A5	0.00000014	0.49	Thyroid
		SLC12A5	0.00000038	43466.00	Uterus
		SLC12A5	0.0000029	0.44	Nerve - Tib
		SNX21	0.0000032	0.2	Muscle - Sk
		SLC12A5	0.0000058	0.41	Skin - Sun E
		SLC12A5	0.000014	0.66	Artery - Co
		SLC12A5	0.000031	0.44	Skin - Not S
0.99	1	*SLC12A5*	0.0000000000000000005	0.63	Lung
		SLC12A5	0.0000000000000034	0.79	Adipose - V
		SLC12A5	0.0000000000083	0.62	Adipose - S
		SLC12A5	0.000000012	0.71	Breast - Ma
		SLC12A5	0.000000055	0.78	Spleen
		SLC12A5	0.00000006	0.65	Artery - Ao
		SLC12A5	0.00000095	0.45	Skin - Sun E
		SLC12A5	0.0000015	0.46	Nerve - Tib
		SLC12A5	0.0000016	0.46	Thyroid
		SLC12A5	0.000002	0.51	Skin - Not S
		SLC12A5	0.0000021	-0.57	Adrenal Gla
		SLC12A5	0.0000038	43466.00	Uterus
		SNX21	0.0000049	0.2	Muscle - Sk
0.98	1	*SLC12A5*	8.9E-22	0.66	Lung
		SLC12A5	0.000000000000000086	0.82	Adipose - V
		SLC12A5	0.0000000000000019	0.71	Adipose - S
		SLC12A5	0.000000000032	0.79	Breast - Ma
		SLC12A5	0.000000000046	0.89	Spleen
		SLC12A5	0.000000033	0.66	Artery - Ao
		SLC12A5	0.00000029	0.48	Thyroid
		SLC12A5	0.00000038	43466.00	Uterus

		SLC12A5	0.00000053	0.47	Nerve - Tib
		SLC12A5	0.0000011	0.44	Skin - Sun E
		SNX21	0.0000065	0.19	Muscle - Sk
		SLC12A5	0.000014	0.66	Artery - Co
		SLC12A5	0.000031	0.44	Skin - Not S
0.98	1	*SLC12A5*	7.5E-22	0.65	Lung
		SLC12A5	0.000000000000000087	0.81	Adipose - V
		SLC12A5	0.000000000000001	0.71	Adipose - S
		SLC12A5	0.000000000032	0.79	Breast - Ma
		SLC12A5	0.00000000004	0.87	Spleen
		SLC12A5	0.000000033	0.66	Artery - Ao
		SLC12A5	0.00000029	0.48	Thyroid
		SLC12A5	0.00000038	43466.00	Uterus
		SLC12A5	0.00000053	0.47	Nerve - Tib
		SLC12A5	0.0000012	0.43	Skin - Sun E
		SNX21	0.0000028	0.2	Muscle - Sk
		SLC12A5	0.000014	0.66	Artery - Co
		SLC12A5	0.000031	0.44	Skin - Not S
0.98	1	*SLC12A5*	3E-21	0.66	Lung
		SLC12A5	0.0000000000000004	0.8	Adipose - V
		SLC12A5	0.000000000000007	0.69	Adipose - S
		SLC12A5	0.000000000099	0.88	Spleen
		SLC12A5	0.00000000036	0.76	Breast - Ma
		SLC12A5	0.0000002	0.49	Thyroid
		SLC12A5	0.00000021	0.62	Artery - Ao
		SLC12A5	0.00000024	0.48	Nerve - Tib
		SLC12A5	0.00000026	43466.00	Uterus
		SLC12A5	0.00000034	0.46	Skin - Sun E
		SLC12A5	0.0000046	0.49	Skin - Not S
		SLC12A5	0.0000056	0.7	Artery - Co
		SNX21	0.000013	0.18	Muscle - Sk
		SLC12A5	0.000019	-0.51	Adrenal Gla
0.96	1	*SLC12A5*	1.7E-21	0.65	Lung
		SLC12A5	0.00000000000000016	0.81	Adipose - V
		SLC12A5	0.0000000000000025	0.7	Adipose - S
		SLC12A5	0.00000000004	0.87	Spleen
		SLC12A5	0.000000000069	0.79	Breast - Ma
		SLC12A5	0.000000056	0.65	Artery - Ao
		SLC12A5	0.00000017	0.5	Thyroid
		SLC12A5	0.00000038	43466.00	Uterus
		SLC12A5	0.00000055	0.47	Nerve - Tib
		SLC12A5	0.0000013	0.43	Skin - Sun E
		SNX21	0.000003	0.2	Muscle - Sk
		SLC12A5	0.000014	0.66	Artery - Co
0.93	0.97	*SLC12A5*	2.1E-21	0.66	Lung
		SLC12A5	0.0000000000000000035	0.81	Adipose - V
		SLC12A5	0.0000000000000039	0.7	Adipose - S

		SLC12A5	0.00000000042	0.76	Breast - Ma
		SLC12A5	0.00000000078	0.87	Spleen
		SLC12A5	0.000000015	0.67	Artery - Ao
		SLC12A5	0.000000095	0.5	Thyroid
		SLC12A5	0.00000061	0.47	Nerve - Tib
		SLC12A5	0.0000016	0.43	Skin - Sun E
		SLC12A5	0.0000024	1.00	Uterus
		SNX21	0.0000031	0.2	Muscle - Sk
		PLTP	0.000017	-0.43	Pituitary
0.82	1	*SLC12A5*	0.0000000000018	0.45	Lung
		SLC12A5	0.000000000023	0.62	Adipose - V
		SLC12A5	0.00000000036	0.51	Adipose - S
		SLC12A5	0.00000028	0.53	Breast - Ma
		SLC12A5	0.000003	0.61	Spleen
		RPL13P2	0.000013	-0.47	Testis
0.89	0.98	*SLC12A5*	8.9E-22	0.66	Lung
		SLC12A5	0.000000000000000086	0.82	Adipose - V
		SLC12A5	0.0000000000000019	0.71	Adipose - S
		SLC12A5	0.000000000032	0.79	Breast - Ma
		SLC12A5	0.000000000046	0.89	Spleen
		SLC12A5	0.000000033	0.66	Artery - Ao
		SLC12A5	0.00000029	0.48	Thyroid
		SLC12A5	0.00000038	43466.00	Uterus
		SLC12A5	0.00000053	0.47	Nerve - Tib
		SLC12A5	0.0000011	0.44	Skin - Sun E
		SNX21	0.0000065	0.19	Muscle - Sk
		SLC12A5	0.000014	0.66	Artery - Co
		SLC12A5	0.000031	0.44	Skin - Not S
0.83	0.94	*SLC12A5*	0.00000000085	-0.48	Adrenal Gla
		NEURL2	0.0000000018	-0.28	Adipose - S
		NEURL2	0.000000097	-0.3	Adipose - V
		ZSWIM1	0.00000023	-0.28	Adipose - V
		PLTP	0.00000072	-0.19	Lung
		RP3-337O18.9	0.00000072	-0.22	Adipose - S
		PLTP	0.000001	-0.18	Artery - Tib
		PLTP	0.0000015	-0.2	Nerve - Tib
		PLTP	0.0000023	-0.25	Heart - Left
		PCIF1	0.0000051	0.32	Adrenal Gla
		SPATA25	0.0000078	-0.28	Adipose - V
		PLTP	0.0000079	-0.27	Colon - Sign
		WFDC3	0.0000087	-0.25	Artery - Tib
		PLTP	0.0000095	-0.17	Thyroid
		CD40	0.000011	0.25	Adipose - V
		PLTP	0.000012	-0.16	Adipose - S
		PLTP	0.000013	-0.16	Adipose - V
		SNX21	0.000029	0.13	Muscle - Sk

		ZNF335	0.000033	0.1	Whole Bloo
		MMP9	0.000045	-0.2	Cells - Tran
0.94	0.99	*SLC12A5*	0.0000000000045	-0.53	Adrenal Gla
		PLTP	0.000000026	-0.23	Lung
		PLTP	0.000000038	-0.23	Nerve - Tib
		PLTP	0.00000025	-0.19	Artery - Tib
		NEURL2	0.00000069	-0.24	Adipose - S
		PCIF1	0.00000087	0.35	Adrenal Gla
		NEURL2	0.0000019	-0.27	Adipose - V
		PLTP	0.0000025	-0.27	Heart - Left
		SNX21	0.0000027	0.15	Muscle - Sk
		PLTP	0.0000048	-0.25	Brain - Hipp
		WFDC3	0.0000083	-0.25	Artery - Tib
		PLTP	0.0000083	-0.31	Adrenal Gla
		PLTP	0.000012	-0.17	Thyroid
		PLTP	0.000014	-0.23	Artery - Ao
		WFDC10B	0.000014	0.39	Testis
		PLTP	0.000016	-0.16	Adipose - S
		RP3-337O18.9	0.000021	-0.2	Adipose - S
		ZSWIM1	0.000025	-0.24	Adipose - V
		WFDC3	0.00003	-0.32	Testis
		NEURL2	0.000032	-0.22	Thyroid
		ZNF335	0.000033	0.1	Whole Bloo
		WFDC3	0.000054	-0.25	Adipose - S
0.94	0.99	*SLC12A5*	0.000000000031	-0.51	Adrenal Gla
		NEURL2	0.000000013	-0.32	Adipose - V
		NEURL2	0.000000052	-0.26	Adipose - S
		ZSWIM1	0.00000019	-0.29	Adipose - V
		PLTP	0.0000004	-0.21	Nerve - Tib
		PCIF1	0.00000079	0.34	Adrenal Gla
		PLTP	0.00000089	-0.2	Lung
		PLTP	0.0000014	-0.18	Artery - Tib
		RP3-337O18.9	0.0000016	-0.22	Adipose - S
		SNX21	0.0000016	0.15	Muscle - Sk
		PLTP	0.0000038	-0.17	Adipose - V
		CD40	0.0000038	0.27	Adipose - V
		PLTP	0.0000096	-0.24	Brain - Hipp
		PLTP	0.00001	-0.24	Heart - Left
		PLTP	0.000012	-0.27	Colon - Sigr
		SPATA25	0.000013	-0.28	Adipose - V
		PLTP	0.000014	-0.16	Adipose - S
		RP3-337O18.9	0.000019	-0.24	Adipose - V
		ZNF335	0.000034	0.1	Whole Bloo
		WFDC3	0.000036	-0.23	Artery - Tib
		WFDC10B	0.00004	0.36	Testis
		MMP9	0.000045	-0.21	Cells - Tran
		PLTP	0.000063	-0.16	Thyroid

0.9	0.96	*SLC12A5*	0.00000000031	-0.49	Adrenal Gla
		NEURL2	0.000000018	-0.32	Adipose - V
		NEURL2	0.00000012	-0.26	Adipose - S
		ZSWIM1	0.00000038	-0.29	Adipose - V
		RP3-337O18.9	0.00000049	-0.23	Adipose - S
		PLTP	0.00000064	-0.21	Nerve - Tib
		PLTP	0.0000013	-0.19	Lung
		PCIF1	0.0000014	0.34	Adrenal Gla
		SNX21	0.0000017	0.15	Muscle - Sk
		PLTP	0.0000023	-0.17	Artery - Tib
		PLTP	0.0000043	-0.17	Adipose - S
		PLTP	0.0000053	-0.17	Adipose - V
		PLTP	0.0000063	-0.28	Colon - Sigr
		WFDC3	0.0000066	-0.26	Artery - Tib
		SPATA25	0.0000077	-0.29	Adipose - V
		PLTP	0.0000079	-0.32	Pituitary
		CD40	0.000022	0.25	Adipose - V
		WFDC10B	0.000031	0.37	Testis
		NEURL2	0.000033	-0.27	Artery - Ao
		RP3-337O18.9	0.000034	-0.23	Adipose - V
		PLTP	0.000035	-0.16	Thyroid
		WFDC3	0.000037	-0.31	Testis
		MMP9	0.000042	-0.21	Cells - Tran
0.85	0.99	*SLC12A5*	0.00000000032	-0.48	Adrenal Gla
		NEURL2	0.000000016	-0.27	Adipose - S
		PLTP	0.00000026	-0.2	Lung
		ZSWIM1	0.00000031	-0.28	Adipose - V
		NEURL2	0.00000054	-0.28	Adipose - V
		PLTP	0.00000075	-0.18	Artery - Tib
		PLTP	0.00000083	-0.27	Heart - Left
		RP3-337O18.9	0.0000014	-0.22	Adipose - S
		PLTP	0.0000021	-0.2	Nerve - Tib
		SPATA25	0.0000036	-0.29	Adipose - V
		PLTP	0.0000037	-0.28	Colon - Sigr
		PLTP	0.0000038	-0.18	Thyroid
		PCIF1	0.0000094	0.31	Adrenal Gla
		CD40	0.000013	0.25	Adipose - V
		PLTP	0.000013	-0.16	Adipose - S
		PLTP	0.000015	-0.15	Adipose - V
		PLTP	0.00002	-0.29	Pituitary
		SNX21	0.000024	0.13	Muscle - Sk
		WFDC10B	0.000025	0.36	Testis
		MMP9	0.00003	-0.21	Cells - Tran
		WFDC3	0.000031	-0.23	Artery - Tib
0.95	0.99	*SLC12A5*	0.00000000004	-0.52	Adrenal Gla
		PLTP	0.00000001	-0.24	Lung
		PLTP	0.000000061	-0.23	Nerve - Tib

		PLTP	0.00000032	-0.19	Artery - Tib
		PCIF1	0.00000099	0.35	Adrenal Gla
		NEURL2	0.0000014	-0.28	Adipose - V
		PLTP	0.0000025	-0.27	Heart - Left
		NEURL2	0.0000026	-0.23	Adipose - S
		SNX21	0.0000034	0.15	Muscle - Sk
		PLTP	0.0000035	-0.22	Brain - Fron
		PLTP	0.0000046	-0.17	Adipose - S
		PLTP	0.0000064	-0.25	Brain - Hipp
		WFDC3	0.0000064	-0.26	Artery - Tib
		PLTP	0.000012	-0.17	Thyroid
		PLTP	0.000014	-0.23	Artery - Ao
		WFDC10B	0.000014	0.39	Testis
		PLTP	0.000019	-0.3	Adrenal Gla
		ZSWIM1	0.000022	-0.24	Adipose - V
		PLTP	0.000026	-0.16	Adipose - V
		NEURL2	0.000029	-0.22	Thyroid
		WFDC3	0.00003	-0.32	Testis
		RP3-337O18.9	0.000033	-0.2	Adipose - S
		NEURL2	0.000043	-0.21	Lung
0.99	0.99	*SLC12A5*	0.00000000000061	-0.57	Adrenal Gla
		PLTP	0.00000000013	-0.26	Lung
		PLTP	0.000000048	-0.24	Nerve - Tib
		PLTP	0.00000028	-0.29	Heart - Left
		PLTP	0.0000003	-0.19	Adipose - S
		PLTP	0.00000052	-0.19	Artery - Tib
		NEURL2	0.0000014	-0.24	Adipose - S
		PLTP	0.0000016	-0.19	Thyroid
		PLTP	0.0000016	-0.23	Brain - Fron
		NEURL2	0.0000019	-0.28	Adipose - V
		PLTP	0.000002	-0.25	Artery - Ao
		PLTP	0.0000032	-0.26	Brain - Hipp
		NEURL2	0.0000046	-0.24	Lung
		PLTP	0.0000047	-0.29	Colon - Sigr
		PCIF1	0.0000073	0.33	Adrenal Gla
		NEURL2	0.0000092	-0.29	Artery - Ao
		RP3-337O18.9	0.0000099	-0.21	Lung
		PLTP	0.000013	-0.31	Adrenal Gla
		ZSWIM1	0.000017	-0.25	Adipose - V
		SNX21	0.00002	0.13	Muscle - Sk
		PLTP	0.000021	-0.16	Adipose - V
		NEURL2	0.000022	-0.23	Thyroid
		ZNF335	0.000025	0.1	Whole Bloo
		RP3-337O18.9	0.000027	-0.2	Adipose - S
0.99	0.99	*SLC12A5*	0.00000000000061	-0.57	Adrenal Gla
		PLTP	0.00000000013	-0.26	Lung
		PLTP	0.000000048	-0.24	Nerve - Tib

		PLTP	0.00000028	-0.29	Heart - Left
		PLTP	0.0000003	-0.19	Adipose - S
		PLTP	0.00000052	-0.19	Artery - Tib
		NEURL2	0.0000014	-0.24	Adipose - S
		PLTP	0.0000016	-0.19	Thyroid
		PLTP	0.0000016	-0.23	Brain - Fron
		NEURL2	0.0000019	-0.28	Adipose - V
		PLTP	0.000002	-0.25	Artery - Ao
		PLTP	0.0000032	-0.26	Brain - Hipp
		NEURL2	0.0000046	-0.24	Lung
		PLTP	0.0000047	-0.29	Colon - Sign
		PCIF1	0.0000073	0.33	Adrenal Gla
		NEURL2	0.0000092	-0.29	Artery - Ao
		RP3-337O18.9	0.0000099	-0.21	Lung
		PLTP	0.000013	-0.31	Adrenal Gla
		ZSWIM1	0.000017	-0.25	Adipose - V
		SNX21	0.00002	0.13	Muscle - Sk
		PLTP	0.000021	-0.16	Adipose - V
		NEURL2	0.000022	-0.23	Thyroid
		ZNF335	0.000025	0.1	Whole Bloo
		RP3-337O18.9	0.000027	-0.2	Adipose - S
0.83	0.92	*PLTP*	0.0000000076	-0.24	Lung
		SLC12A5	0.0000000082	-0.48	Adrenal Gla
		PLTP	0.00000003	-0.2	Artery - Tib
		PLTP	0.000000057	-0.23	Nerve - Tib
		NEURL2	0.0000015	-0.27	Adipose - V
		PLTP	0.0000015	-0.17	Adipose - V
		PLTP	0.0000018	-0.26	Heart - Left
		PCIF1	0.0000021	0.35	Adrenal Gla
		NEURL2	0.0000022	-0.23	Adipose - S
		PLTP	0.000004	-0.24	Artery - Ao
		PLTP	0.0000059	-0.17	Adipose - S
		PLTP	0.0000066	-0.24	Brain - Hipp
		NEURL2	0.00001	-0.29	Artery - Ao
		ZSWIM1	0.000012	-0.25	Adipose - V
		RP3-337O18.9	0.000012	-0.21	Lung
		PLTP	0.000019	-0.27	Colon - Sign
		PLTP	0.00002	-0.31	Pituitary
		WFDC3	0.000024	-0.24	Artery - Tib
		NEURL2	0.000024	-0.22	Lung
		SNX21	0.000028	0.13	Muscle - Sk
		RP3-337O18.9	0.00004	-0.19	Adipose - S
		PLTP	0.000041	-0.16	Thyroid
		NEURL2	0.000044	-0.22	Thyroid
0.99	0.99	*SLC12A5*	0.0000000000043	-0.57	Adrenal Gla
		PLTP	0.00000000033	-0.26	Lung
		PLTP	0.000000026	-0.32	Heart - Left

		PLTP	0.000000034	-0.24	Nerve - Tib
		PLTP	0.00000021	-0.2	Adipose - S
		NEURL2	0.00000026	-0.3	Adipose - V
		PLTP	0.00000046	-0.2	Thyroid
		PLTP	0.00000055	-0.19	Artery - Tib
		PLTP	0.00000066	-0.37	Adrenal Gla
		NEURL2	0.0000015	-0.24	Adipose - S
		PLTP	0.000002	-0.25	Artery - Ao
		NEURL2	0.0000021	-0.31	Artery - Ao
		PLTP	0.0000024	-0.18	Adipose - V
		PLTP	0.0000025	-0.3	Colon - Sigi
		PLTP	0.0000033	-0.22	Brain - Fror
		PCIF1	0.0000041	0.35	Adrenal Gla
		ZSWIM1	0.0000068	-0.26	Adipose - V
		RP3-337O18.9	0.0000076	-0.22	Lung
		PLTP	0.0000091	-0.32	Pituitary
		SNX21	0.000012	0.14	Muscle - Sk
		RP3-337O18.9	0.000028	-0.2	Adipose - S
		NEURL2	0.000036	-0.22	Lung
		NEURL2	0.000073	-0.22	Thyroid
0.82	-0.98	*SLC12A5*	0.000000000012	0.5	Adrenal Gla
		PLTP	0.000000000041	0.28	Nerve - Tib
		NEURL2	0.000000000047	0.35	Adipose - V
		PLTP	0.00000000011	0.23	Artery - Tib
		PLTP	0.0000000028	0.24	Lung
		NEURL2	0.000000004	0.27	Adipose - S
		PLTP	0.0000000059	0.35	Colon - Sigi
		ZSWIM1	0.0000000059	0.31	Adipose - V
		PLTP	0.000000034	0.2	Adipose - S
		RP3-337O18.9	0.000000051	0.29	Adipose - V
		RP3-337O18.9	0.00000013	0.24	Lung
		SPATA25	0.00000014	0.32	Adipose - V
		RP3-337O18.9	0.00000019	0.23	Adipose - S
		PLTP	0.00000023	0.18	Adipose - V
		PLTP	0.00000091	0.25	Artery - Ao
		SNX21	0.000001	-0.15	Muscle - Sk
		CD40	0.0000017	-0.27	Adipose - V
		NEURL2	0.0000044	0.23	Lung
		NEURL2	0.0000055	0.29	Artery - Ao
		PLTP	0.000013	0.24	Stomach
		PCIF1	0.000013	-0.3	Adrenal Gla
		SNX21	0.000022	0.17	Thyroid
		PLTP	0.000024	0.16	Thyroid
		CD40	0.000047	-0.19	Skin - Sun E
0.82	0.94	*SLC12A5*	0.00000000085	-0.48	Adrenal Gla
		NEURL2	0.0000000018	-0.28	Adipose - S

		NEURL2	0.000000097	-0.3	Adipose - V
		ZSWIM1	0.00000023	-0.28	Adipose - V
		PLTP	0.00000072	-0.19	Lung
		RP3-337O18.9	0.00000072	-0.22	Adipose - S
		PLTP	0.000001	-0.18	Artery - Tib
		PLTP	0.0000015	-0.2	Nerve - Tib
		PLTP	0.0000023	-0.25	Heart - Left
		PCIF1	0.0000051	0.32	Adrenal Gla
		SPATA25	0.0000078	-0.28	Adipose - V
		PLTP	0.0000079	-0.27	Colon - Sign
		WFDC3	0.0000087	-0.25	Artery - Tib
		PLTP	0.0000095	-0.17	Thyroid
		CD40	0.000011	0.25	Adipose - V
		PLTP	0.000012	-0.16	Adipose - S
		PLTP	0.000013	-0.16	Adipose - V
		SNX21	0.000029	0.13	Muscle - Sk
		ZNF335	0.000033	0.1	Whole Bloo
		MMP9	0.000045	-0.2	Cells - Tran
0.92	0.99	*SLC12A5*	0.0000000000045	-0.53	Adrenal Gla
		PLTP	0.000000026	-0.23	Lung
		PLTP	0.000000038	-0.23	Nerve - Tib
		PLTP	0.00000025	-0.19	Artery - Tib
		NEURL2	0.00000069	-0.24	Adipose - S
		PCIF1	0.00000087	0.35	Adrenal Gla
		NEURL2	0.0000019	-0.27	Adipose - V
		PLTP	0.0000025	-0.27	Heart - Left
		SNX21	0.0000027	0.15	Muscle - Sk
		PLTP	0.0000048	-0.25	Brain - Hipp
		WFDC3	0.0000083	-0.25	Artery - Tib
		PLTP	0.0000083	-0.31	Adrenal Gla
		PLTP	0.000012	-0.17	Thyroid
		PLTP	0.000014	-0.23	Artery - Ao
		WFDC10B	0.000014	0.39	Testis
		PLTP	0.000016	-0.16	Adipose - S
		RP3-337O18.9	0.000021	-0.2	Adipose - S
		ZSWIM1	0.000025	-0.24	Adipose - V
		WFDC3	0.00003	-0.32	Testis
		NEURL2	0.000032	-0.22	Thyroid
		ZNF335	0.000033	0.1	Whole Bloo
		WFDC3	0.000054	-0.25	Adipose - S
0.92	0.99	*SLC12A5*	0.000000000031	-0.51	Adrenal Gla
		NEURL2	0.000000013	-0.32	Adipose - V
		NEURL2	0.000000052	-0.26	Adipose - S
		ZSWIM1	0.00000019	-0.29	Adipose - V
		PLTP	0.0000004	-0.21	Nerve - Tib
		PCIF1	0.00000079	0.34	Adrenal Gla
		PLTP	0.00000089	-0.2	Lung

		PLTP	0.0000014	-0.18	Artery - Tib
		RP3-337O18.9	0.0000016	-0.22	Adipose - S
		SNX21	0.0000016	0.15	Muscle - Sk
		PLTP	0.0000038	-0.17	Adipose - V
		CD40	0.0000038	0.27	Adipose - V
		PLTP	0.0000096	-0.24	Brain - Hipp
		PLTP	0.00001	-0.24	Heart - Left
		PLTP	0.000012	-0.27	Colon - Sigr
		SPATA25	0.000013	-0.28	Adipose - V
		PLTP	0.000014	-0.16	Adipose - S
		RP3-337O18.9	0.000019	-0.24	Adipose - V
		ZNF335	0.000034	0.1	Whole Bloo
		WFDC3	0.000036	-0.23	Artery - Tib
		WFDC10B	0.00004	0.36	Testis
		MMP9	0.000045	-0.21	Cells - Tran
		PLTP	0.000063	-0.16	Thyroid
0.89	0.96	*SLC12A5*	0.000000000031	-0.51	Adrenal Gla
		NEURL2	0.000000013	-0.32	Adipose - V
		NEURL2	0.000000052	-0.26	Adipose - S
		ZSWIM1	0.00000019	-0.29	Adipose - V
		PLTP	0.0000004	-0.21	Nerve - Tib
		PCIF1	0.00000079	0.34	Adrenal Gla
		PLTP	0.00000089	-0.2	Lung
		PLTP	0.0000014	-0.18	Artery - Tib
		RP3-337O18.9	0.0000016	-0.22	Adipose - S
		SNX21	0.0000016	0.15	Muscle - Sk
		PLTP	0.0000038	-0.17	Adipose - V
		CD40	0.0000038	0.27	Adipose - V
		PLTP	0.0000096	-0.24	Brain - Hipp
		PLTP	0.00001	-0.24	Heart - Left
		PLTP	0.000012	-0.27	Colon - Sigr
		SPATA25	0.000013	-0.28	Adipose - V
		PLTP	0.000014	-0.16	Adipose - S
		RP3-337O18.9	0.000019	-0.24	Adipose - V
		ZNF335	0.000034	0.1	Whole Bloo
		WFDC3	0.000036	-0.23	Artery - Tib
		WFDC10B	0.00004	0.36	Testis
		MMP9	0.000045	-0.21	Cells - Tran
		PLTP	0.000063	-0.16	Thyroid
0.84	0.99	*SLC12A5*	0.00000000032	-0.48	Adrenal Gla
		NEURL2	0.000000016	-0.27	Adipose - S
		PLTP	0.00000026	-0.2	Lung
		ZSWIM1	0.00000031	-0.28	Adipose - V
		NEURL2	0.00000054	-0.28	Adipose - V
		PLTP	0.00000075	-0.18	Artery - Tib
		PLTP	0.00000083	-0.27	Heart - Left
		RP3-337O18.9	0.0000014	-0.22	Adipose - S

		PLTP	0.0000021	-0.2	Nerve - Tib
		SPATA25	0.0000036	-0.29	Adipose - V
		PLTP	0.0000037	-0.28	Colon - Sigr
		PLTP	0.0000038	-0.18	Thyroid
		PCIF1	0.0000094	0.31	Adrenal Gla
		CD40	0.000013	0.25	Adipose - V
		PLTP	0.000013	-0.16	Adipose - S
		PLTP	0.000015	-0.15	Adipose - V
		PLTP	0.00002	-0.29	Pituitary
		SNX21	0.000024	0.13	Muscle - Sk
		WFDC10B	0.000025	0.36	Testis
		MMP9	0.00003	-0.21	Cells - Tran
		WFDC3	0.000031	-0.23	Artery - Tib
0.94	0.99	*SLC12A5*	0.00000000004	-0.52	Adrenal Gla
		PLTP	0.00000001	-0.24	Lung
		PLTP	0.000000061	-0.23	Nerve - Tib
		PLTP	0.00000032	-0.19	Artery - Tib
		PCIF1	0.00000099	0.35	Adrenal Gla
		NEURL2	0.0000014	-0.28	Adipose - V
		PLTP	0.0000025	-0.27	Heart - Left
		NEURL2	0.0000026	-0.23	Adipose - S
		SNX21	0.0000034	0.15	Muscle - Sk
		PLTP	0.0000035	-0.22	Brain - Fror
		PLTP	0.0000046	-0.17	Adipose - S
		PLTP	0.0000064	-0.25	Brain - Hipp
		WFDC3	0.0000064	-0.26	Artery - Tib
		PLTP	0.000012	-0.17	Thyroid
		PLTP	0.000014	-0.23	Artery - Ao
		WFDC10B	0.000014	0.39	Testis
		PLTP	0.000019	-0.3	Adrenal Gla
		ZSWIM1	0.000022	-0.24	Adipose - V
		PLTP	0.000026	-0.16	Adipose - V
		NEURL2	0.000029	-0.22	Thyroid
		WFDC3	0.00003	-0.32	Testis
		RP3-337O18.9	0.000033	-0.2	Adipose - S
		NEURL2	0.000043	-0.21	Lung
0.99	0.98	*SLC12A5*	0.00000000000061	-0.57	Adrenal Gla
		PLTP	0.00000000013	-0.26	Lung
		PLTP	0.000000048	-0.24	Nerve - Tib
		PLTP	0.00000028	-0.29	Heart - Left
		PLTP	0.0000003	-0.19	Adipose - S
		PLTP	0.00000052	-0.19	Artery - Tib
		NEURL2	0.0000014	-0.24	Adipose - S
		PLTP	0.0000016	-0.19	Thyroid
		PLTP	0.0000016	-0.23	Brain - Fror
		NEURL2	0.0000019	-0.28	Adipose - V
		PLTP	0.000002	-0.25	Artery - Ao

		PLTP	0.0000032	-0.26	Brain - Hipp
		NEURL2	0.0000046	-0.24	Lung
		PLTP	0.0000047	-0.29	Colon - Sigr
		PCIF1	0.0000073	0.33	Adrenal Gla
		NEURL2	0.0000092	-0.29	Artery - Ao
		RP3-337O18.9	0.0000099	-0.21	Lung
		PLTP	0.000013	-0.31	Adrenal Gla
		ZSWIM1	0.000017	-0.25	Adipose - V
		SNX21	0.00002	0.13	Muscle - Sk
		PLTP	0.000021	-0.16	Adipose - V
		NEURL2	0.000022	-0.23	Thyroid
		ZNF335	0.000025	0.1	Whole Bloo
		RP3-337O18.9	0.000027	-0.2	Adipose - S
0.99	0.98	*SLC12A5*	0.00000000000061	-0.57	Adrenal Gla
		PLTP	0.00000000013	-0.26	Lung
		PLTP	0.000000048	-0.24	Nerve - Tib
		PLTP	0.00000028	-0.29	Heart - Left
		PLTP	0.0000003	-0.19	Adipose - S
		PLTP	0.00000052	-0.19	Artery - Tib
		NEURL2	0.0000014	-0.24	Adipose - S
		PLTP	0.0000016	-0.19	Thyroid
		PLTP	0.0000016	-0.23	Brain - Fron
		NEURL2	0.0000019	-0.28	Adipose - V
		PLTP	0.000002	-0.25	Artery - Ao
		PLTP	0.0000032	-0.26	Brain - Hipp
		NEURL2	0.0000046	-0.24	Lung
		PLTP	0.0000047	-0.29	Colon - Sigr
		PCIF1	0.0000073	0.33	Adrenal Gla
		NEURL2	0.0000092	-0.29	Artery - Ao
		RP3-337O18.9	0.0000099	-0.21	Lung
		PLTP	0.000013	-0.31	Adrenal Gla
		ZSWIM1	0.000017	-0.25	Adipose - V
		SNX21	0.00002	0.13	Muscle - Sk
		PLTP	0.000021	-0.16	Adipose - V
		NEURL2	0.000022	-0.23	Thyroid
		ZNF335	0.000025	0.1	Whole Bloo
		RP3-337O18.9	0.000027	-0.2	Adipose - S
0.99	0.99	*SLC12A5*	0.00000000000012	-0.57	Adrenal Gla
		NEURL2	0.000000015	-0.32	Adipose - V
		PLTP	0.000000025	-0.23	Lung
		PLTP	0.000000076	-0.2	Adipose - S
		NEURL2	0.000000093	-0.26	Adipose - S
		PLTP	0.00000015	-0.23	Nerve - Tib
		PLTP	0.00000017	-0.32	Colon - Sigr
		ZSWIM1	0.00000025	-0.29	Adipose - V
		CD40	0.00000082	0.29	Adipose - V
		PLTP	0.000001	-0.18	Artery - Tib

		PLTP	0.000001	-0.27	Heart - Left
		PLTP	0.0000015	-0.18	Adipose - V
		NEURL2	0.0000018	-0.31	Artery - Ao
		SNX21	0.000003	0.15	Muscle - Sk
		PCIF1	0.0000041	0.33	Adrenal Gla
		PLTP	0.0000062	-0.18	Thyroid
		RP3-337O18.9	0.0000072	-0.21	Adipose - S
		PLTP	0.0000088	-0.32	Pituitary
		PLTP	0.0000089	-0.21	Brain - Fron
		RP3-337O18.9	0.0000089	-0.21	Lung
		PLTP	0.00001	-0.23	Artery - Ao
		ZNF335	0.000011	0.11	Whole Bloo
		RP3-337O18.9	0.000015	-0.25	Adipose - V
		PLTP	0.000017	-0.25	Stomach
		SPATA25	0.000019	-0.28	Adipose - V
		NEURL2	0.000043	-0.21	Lung
0.84	0.93	*PLTP*	0.0000000076	-0.24	Lung
		SLC12A5	0.0000000082	-0.48	Adrenal Gla
		PLTP	0.00000003	-0.2	Artery - Tib
		PLTP	0.000000057	-0.23	Nerve - Tib
		NEURL2	0.0000015	-0.27	Adipose - V
		PLTP	0.0000015	-0.17	Adipose - V
		PLTP	0.0000018	-0.26	Heart - Left
		PCIF1	0.0000021	0.35	Adrenal Gla
		NEURL2	0.0000022	-0.23	Adipose - S
		PLTP	0.000004	-0.24	Artery - Ao
		PLTP	0.0000059	-0.17	Adipose - S
		PLTP	0.0000066	-0.24	Brain - Hipp
		NEURL2	0.00001	-0.29	Artery - Ao
		ZSWIM1	0.000012	-0.25	Adipose - V
		RP3-337O18.9	0.000012	-0.21	Lung
		PLTP	0.000019	-0.27	Colon - Sigr
		PLTP	0.00002	-0.31	Pituitary
		WFDC3	0.000024	-0.24	Artery - Tib
		NEURL2	0.000024	-0.22	Lung
		SNX21	0.000028	0.13	Muscle - Sk
		RP3-337O18.9	0.00004	-0.19	Adipose - S
		PLTP	0.000041	-0.16	Thyroid
		NEURL2	0.000044	-0.22	Thyroid
0.83	-0.98	*SLC12A5*	0.000000000012	0.5	Adrenal Gla
		PLTP	0.000000000041	0.28	Nerve - Tib
		NEURL2	0.000000000047	0.35	Adipose - V
		PLTP	0.00000000011	0.23	Artery - Tib
		PLTP	0.0000000028	0.24	Lung
		NEURL2	0.000000004	0.27	Adipose - S
		PLTP	0.0000000059	0.35	Colon - Sigr
		ZSWIM1	0.0000000059	0.31	Adipose - V

		PLTP	0.000000034	0.2	Adipose - S
		RP3-337O18.9	0.000000051	0.29	Adipose - V
		RP3-337O18.9	0.00000013	0.24	Lung
		SPATA25	0.00000014	0.32	Adipose - V
		RP3-337O18.9	0.00000019	0.23	Adipose - S
		PLTP	0.00000023	0.18	Adipose - V
		PLTP	0.00000091	0.25	Artery - Ao
		SNX21	0.000001	-0.15	Muscle - Sk
		CD40	0.0000017	-0.27	Adipose - V
		NEURL2	0.0000044	0.23	Lung
		NEURL2	0.0000055	0.29	Artery - Ao
		PLTP	0.000013	0.24	Stomach
		PCIF1	0.000013	-0.3	Adrenal Gla
		SNX21	0.000022	0.17	Thyroid
		PLTP	0.000024	0.16	Thyroid
		CD40	0.000047	-0.19	Skin - Sun E
0.87	0.94	*NEURL2*	0.0000011	-0.28	Adipose - S
		RP3-337O18.9	0.0000024	-0.27	Lung
		PLTP	0.0000041	-0.39	Adrenal Gla
		PLTP	0.0000058	-0.22	Lung
		SLC12A5	0.0000061	-0.44	Adipose - V
		NEURL2	0.000012	-0.31	Adipose - V
		RP3-337O18.9	0.000019	-0.3	Adipose - V
		SPATA25	0.000029	-0.33	Adipose - V
		PLTP	0.00005	-0.19	Thyroid
0.86	0.94	*NEURL2*	0.0000009	-0.28	Adipose - S
		RP3-337O18.9	0.0000017	-0.27	Lung
		SLC12A5	0.0000044	-0.44	Adipose - V
		PLTP	0.0000048	-0.38	Adrenal Gla
		PLTP	0.0000053	-0.22	Lung
		NEURL2	0.000012	-0.31	Adipose - V
		RP3-337O18.9	0.000021	-0.29	Adipose - V
		PLTP	0.000026	-0.25	Artery - Ao
		SPATA25	0.000026	-0.33	Adipose - V
		PLTP	0.000046	-0.17	Artery - Tib
0.89	0.98	*NEURL2*	0.00000085	-0.29	Adipose - S
		SLC12A5	0.0000019	-0.46	Adipose - V
		RP3-337O18.9	0.0000024	-0.27	Lung
		PLTP	0.0000024	-0.23	Lung
		PLTP	0.000016	-0.35	Adrenal Gla
		PLTP	0.000055	-0.17	Artery - Tib
0.98	0.99	*NEURL2*	0.00000085	-0.28	Adipose - S
		SLC12A5	0.0000019	-0.46	Adipose - V
		RP3-337O18.9	0.0000024	-0.28	Lung
		PLTP	0.0000024	-0.24	Lung
		PLTP	0.000016	-0.37	Adrenal Gla

1	1	*SLC12A5*	0.00000022	-0.5	Adipose - V
		PLTP	0.0000014	-0.24	Lung
		NEURL2	0.0000021	-0.28	Adipose - S
		PLTP	0.0000038	-0.39	Adrenal Gla
		RP3-337O18.9	0.0000044	-0.26	Lung
		CD40	0.000025	0.24	Skin - Sun E
		PLTP	0.000036	-0.18	Artery - Tib
0.95	0.98	*SLC12A5*	0.00000022	-0.5	Adipose - V
		RP3-337O18.9	0.0000037	-0.27	Lung
		PLTP	0.0000039	-0.23	Lung
		PLTP	0.0000051	-0.39	Adrenal Gla
		NEURL2	0.0000066	-0.27	Adipose - S
		CD40	0.00003	0.24	Skin - Sun E
		PLTP	0.00005	-0.18	Artery - Tib
0.95	0.98	*SLC12A5*	0.00000022	-0.5	Adipose - V
		PLTP	0.0000023	-0.24	Lung
		RP3-337O18.9	0.0000032	-0.27	Lung
		PLTP	0.0000038	-0.39	Adrenal Gla
		NEURL2	0.0000066	-0.27	Adipose - S
		CD40	0.00003	0.24	Skin - Sun E
		PLTP	0.00005	-0.18	Artery - Tib
0.95	0.98	*SLC12A5*	0.00000022	-0.5	Adipose - V
		PLTP	0.0000023	-0.24	Lung
		RP3-337O18.9	0.0000032	-0.27	Lung
		PLTP	0.0000038	-0.39	Adrenal Gla
		NEURL2	0.0000066	-0.27	Adipose - S
		CD40	0.00003	0.24	Skin - Sun E
		PLTP	0.00005	-0.18	Artery - Tib
0.95	0.98	*SLC12A5*	0.00000022	-0.5	Adipose - V
		PLTP	0.0000023	-0.24	Lung
		RP3-337O18.9	0.0000032	-0.27	Lung
		PLTP	0.0000038	-0.39	Adrenal Gla
		NEURL2	0.0000066	-0.27	Adipose - S
		CD40	0.00003	0.24	Skin - Sun E
		PLTP	0.00005	-0.18	Artery - Tib
0.95	0.98	*SLC12A5*	0.00000022	-0.5	Adipose - V
		PLTP	0.0000023	-0.24	Lung
		RP3-337O18.9	0.0000032	-0.27	Lung
		PLTP	0.0000038	-0.39	Adrenal Gla
		NEURL2	0.0000066	-0.27	Adipose - S
		CD40	0.00003	0.24	Skin - Sun E
		PLTP	0.00005	-0.18	Artery - Tib
0.91	0.97	*SLC12A5*	0.00000022	-0.5	Adipose - V
		RP3-337O18.9	0.0000037	-0.27	Lung
		PLTP	0.0000039	-0.23	Lung
		PLTP	0.0000051	-0.39	Adrenal Gla
		NEURL2	0.0000066	-0.27	Adipose - S

		CD40	0.00003	0.24	Skin - Sun E
		PLTP	0.00005	-0.18	Artery - Tib
0.94	0.98	*SLC12A5*	0.00000036	-0.49	Adipose - V
		PLTP	0.0000033	-0.23	Lung
		PLTP	0.0000051	-0.39	Adrenal Gla
		NEURL2	0.0000066	-0.27	Adipose - S
		RP3-337O18.9	0.000011	-0.26	Lung
		PLTP	0.000029	-0.26	Artery - Ao
		PLTP	0.000057	-0.18	Artery - Tib
0.92	0.97	*PLTP*	0.000016	0.39	Esophagus
		PLTP	0.000035	0.33	Skin - Sun E
0.94	0.98	*SLC12A5*	0.0000000000000019	0.58	Lung
		SLC12A5	0.00000000000022	0.75	Adipose - V
		SLC12A5	0.0000000053	0.54	Adipose - S
		SLC12A5	0.00000012	-0.62	Adrenal Gla
		SLC12A5	0.00000015	0.63	Artery - Ao
		SLC12A5	0.00000033	0.66	Breast - Ma
		SLC12A5	0.00000046	0.74	Spleen
		SNX21	0.0000006	0.21	Muscle - Sk
		SLC12A5	0.0000026	0.46	Thyroid
		SLC12A5	0.00001	0.4	Nerve - Tib
		SLC12A5	0.000022	0.46	Skin - Not S
		PLTP	0.000031	-0.24	Nerve - Tib
		SLC12A5	0.000034	0.38	Skin - Sun E
0.94	0.98	*SLC12A5*	0.0000000000000019	0.58	Lung
		SLC12A5	0.00000000000022	0.75	Adipose - V
		SLC12A5	0.0000000053	0.54	Adipose - S
		SLC12A5	0.000000098	-0.63	Adrenal Gla
		SLC12A5	0.00000015	0.63	Artery - Ao
		SLC12A5	0.00000023	0.67	Breast - Ma
		SLC12A5	0.00000025	0.77	Spleen
		SNX21	0.0000006	0.21	Muscle - Sk
		SLC12A5	0.000007	0.44	Thyroid
		SLC12A5	0.00001	0.4	Nerve - Tib
		SLC12A5	0.000028	0.46	Skin - Not S
		PLTP	0.000031	-0.24	Nerve - Tib
		SLC12A5	0.000034	0.38	Skin - Sun E
0.84	0.98	*SLC12A5*	0.000000000000033	0.8	Adipose - V
		SLC12A5	0.00000000000026	0.54	Lung
		SLC12A5	0.00000004	0.84	Spleen
		SLC12A5	0.00000012	0.51	Adipose - S
		SLC12A5	0.00000026	0.64	Artery - Ao
		SNX21	0.00000039	0.23	Muscle - Sk
		SLC12A5	0.0000046	-0.57	Adrenal Gla
		SLC12A5	0.000014	0.41	Nerve - Tib

		PLTP	0.000018	-0.26	Nerve - Tib
		PLTP	0.000019	-0.28	Lung
		SLC12A5	0.000021	0.59	Breast - Ma
		SLC12A5	0.000025	0.4	Skin - Sun E
		SLC12A5	0.000028	0.42	Thyroid
		SLC12A5	0.000036	0.47	Skin - Not S
		WFDC3	0.000058	-0.4	Nerve - Tib
0.98	1	*SLC12A5*	0.0000000000000000059	0.61	Lung
		SLC12A5	0.0000000000000016	0.8	Adipose - V
		SLC12A5	0.00000000002	0.61	Adipose - S
		SLC12A5	0.000000045	0.69	Breast - Ma
		SLC12A5	0.000000082	0.63	Artery - Ao
		SLC12A5	0.00000018	0.78	Spleen
		SNX21	0.00000098	0.21	Muscle - Sk
		SLC12A5	0.0000023	-0.56	Adrenal Gla
		SLC12A5	0.0000024	0.43	Nerve - Tib
		SLC12A5	0.0000024	0.45	Thyroid
		SLC12A5	0.0000035	43466.00	Uterus
		SLC12A5	0.0000054	0.41	Skin - Sun E
		SLC12A5	0.00001	0.48	Skin - Not S
		PLTP	0.000056	-0.24	Nerve - Tib
0.98	1	*SLC12A5*	0.0000000000000000059	0.61	Lung
		SLC12A5	0.0000000000000016	0.8	Adipose - V
		SLC12A5	0.000000000045	0.6	Adipose - S
		SLC12A5	0.000000045	0.69	Breast - Ma
		SLC12A5	0.000000082	0.63	Artery - Ao
		SLC12A5	0.00000018	0.78	Spleen
		SLC12A5	0.00000019	-0.61	Adrenal Gla
		SNX21	0.00000099	0.21	Muscle - Sk
		SLC12A5	0.0000029	0.45	Thyroid
		SLC12A5	0.0000037	0.43	Nerve - Tib
		SLC12A5	0.0000038	43466.00	Uterus
		SLC12A5	0.0000053	0.41	Skin - Sun E
		SLC12A5	0.00001	0.48	Skin - Not S
		PLTP	0.000028	-0.25	Nerve - Tib
0.99	1	*SLC12A5*	8.9E-22	0.66	Lung
		SLC12A5	0.0000000000000002	0.81	Adipose - V
		SLC12A5	0.00000000000003	0.68	Adipose - S
		SLC12A5	0.00000000011	0.77	Breast - Ma
		SLC12A5	0.00000000015	0.88	Spleen
		SLC12A5	0.000000023	0.67	Artery - Ao
		SLC12A5	0.00000014	0.49	Thyroid
		SLC12A5	0.00000038	43466.00	Uterus
		SLC12A5	0.0000029	0.44	Nerve - Tib
		SNX21	0.0000032	0.2	Muscle - Sk
		SLC12A5	0.0000058	0.41	Skin - Sun E
		SLC12A5	0.000014	0.66	Artery - Co

		SLC12A5	0.000031	0.44	Skin - Not S
0.99	1	*SLC12A5*	0.0000000000000000005	0.63	Lung
		SLC12A5	0.0000000000000034	0.79	Adipose - V
		SLC12A5	0.0000000000083	0.62	Adipose - S
		SLC12A5	0.000000012	0.71	Breast - Ma
		SLC12A5	0.000000055	0.78	Spleen
		SLC12A5	0.00000006	0.65	Artery - Ao
		SLC12A5	0.00000095	0.45	Skin - Sun E
		SLC12A5	0.0000015	0.46	Nerve - Tib
		SLC12A5	0.0000016	0.46	Thyroid
		SLC12A5	0.000002	0.51	Skin - Not S
		SLC12A5	0.0000021	-0.57	Adrenal Gla
		SLC12A5	0.0000038	43466.00	Uterus
		SNX21	0.0000049	0.2	Muscle - Sk
1	1	*SLC12A5*	7.5E-22	0.65	Lung
		SLC12A5	0.000000000000000087	0.81	Adipose - V
		SLC12A5	0.000000000000001	0.71	Adipose - S
		SLC12A5	0.00000000032	0.79	Breast - Ma
		SLC12A5	0.00000000004	0.87	Spleen
		SLC12A5	0.000000033	0.66	Artery - Ao
		SLC12A5	0.00000029	0.48	Thyroid
		SLC12A5	0.00000038	43466.00	Uterus
		SLC12A5	0.00000053	0.47	Nerve - Tib
		SLC12A5	0.0000012	0.43	Skin - Sun E
		SNX21	0.0000028	0.2	Muscle - Sk
		SLC12A5	0.000014	0.66	Artery - Co
		SLC12A5	0.000031	0.44	Skin - Not S
1	1	*SLC12A5*	3E-21	0.66	Lung
		SLC12A5	0.0000000000000004	0.8	Adipose - V
		SLC12A5	0.000000000000007	0.69	Adipose - S
		SLC12A5	0.000000000099	0.88	Spleen
		SLC12A5	0.00000000036	0.76	Breast - Ma
		SLC12A5	0.0000002	0.49	Thyroid
		SLC12A5	0.00000021	0.62	Artery - Ao
		SLC12A5	0.00000024	0.48	Nerve - Tib
		SLC12A5	0.00000026	43466.00	Uterus
		SLC12A5	0.00000034	0.46	Skin - Sun E
		SLC12A5	0.0000046	0.49	Skin - Not S
		SLC12A5	0.0000056	0.7	Artery - Co
		SNX21	0.000013	0.18	Muscle - Sk
		SLC12A5	0.000019	-0.51	Adrenal Gla
0.98	1	*SLC12A5*	1.7E-21	0.65	Lung
		SLC12A5	0.00000000000000016	0.81	Adipose - V
		SLC12A5	0.0000000000000025	0.7	Adipose - S
		SLC12A5	0.00000000004	0.87	Spleen
		SLC12A5	0.000000000069	0.79	Breast - Ma
		SLC12A5	0.000000056	0.65	Artery - Ao

		SLC12A5	0.00000017	0.5	Thyroid
		SLC12A5	0.00000038	43466.00	Uterus
		SLC12A5	0.00000055	0.47	Nerve - Tib
		SLC12A5	0.0000013	0.43	Skin - Sun E
		SNX21	0.000003	0.2	Muscle - Sk
		SLC12A5	0.000014	0.66	Artery - Co
0.95	0.97	*SLC12A5*	2.1E-21	0.66	Lung
		SLC12A5	0.00000000000000035	0.81	Adipose - V
		SLC12A5	0.0000000000000039	0.7	Adipose - S
		SLC12A5	0.00000000042	0.76	Breast - Ma
		SLC12A5	0.00000000078	0.87	Spleen
		SLC12A5	0.000000015	0.67	Artery - Ao
		SLC12A5	0.000000095	0.5	Thyroid
		SLC12A5	0.00000061	0.47	Nerve - Tib
		SLC12A5	0.0000016	0.43	Skin - Sun E
		SLC12A5	0.0000024	1.00	Uterus
		SNX21	0.0000031	0.2	Muscle - Sk
		PLTP	0.000017	-0.43	Pituitary
0.84	1	*SLC12A5*	0.0000000000018	0.45	Lung
		SLC12A5	0.000000000023	0.62	Adipose - V
		SLC12A5	0.00000000036	0.51	Adipose - S
		SLC12A5	0.00000028	0.53	Breast - Ma
		SLC12A5	0.000003	0.61	Spleen
		RPL13P2	0.000013	-0.47	Testis
0.91	0.98	*SLC12A5*	8.9E-22	0.66	Lung
		SLC12A5	0.000000000000000086	0.82	Adipose - V
		SLC12A5	0.0000000000000019	0.71	Adipose - S
		SLC12A5	0.000000000032	0.79	Breast - Ma
		SLC12A5	0.000000000046	0.89	Spleen
		SLC12A5	0.000000033	0.66	Artery - Ao
		SLC12A5	0.00000029	0.48	Thyroid
		SLC12A5	0.00000038	43466.00	Uterus
		SLC12A5	0.00000053	0.47	Nerve - Tib
		SLC12A5	0.0000011	0.44	Skin - Sun E
		SNX21	0.0000065	0.19	Muscle - Sk
		SLC12A5	0.000014	0.66	Artery - Co
		SLC12A5	0.000031	0.44	Skin - Not S
