# Supplementary material for: Matrix Metalloproteinase Gene Polymorphisms Are Associated with Breast Cancer in the Caucasian Women of Russia
Source: Int J Mol Sci. 2022 Oct 20;23(20):12638. doi: 10.3390/ijms232012638 (PMC9604098; doi:10.3390/ijms232012638)
Supplement: Supplementary file 1 [file ijms-23-12638-s001.zip › Suppl table s9.pdf]

Supplementary table S9 sQTL values of the BC-associated SNPs of
(according to Genotype-Tissue Expression (GTEx) (http://www.gt

Chr	SNP	Gene Symbol	Variant Id
11	rs1799750	*WTAPP1*	chr11_102799764_TC_T_b38
11	rs679620	*WTAPP1*	chr11_102842889_T_C_b38
20	rs3918242	*CD40*	chr20_46007337_C_T_b38
		CD40	chr20_46007337_C_T_b38
		CD40	chr20_46007337_C_T_b38
		SLC12A5	chr20_46007337_C_T_b38
		CD40	chr20_46007337_C_T_b38
		ACOT8	chr20_46007337_C_T_b38
		CD40	chr20_46007337_C_T_b38
		ACOT8	chr20_46007337_C_T_b38
20	rs3918249	*SLC12A5*	chr20_46009497_T_C_b38
		SLC12A5	chr20_46009497_T_C_b38
		SLC12A5	chr20_46009497_T_C_b38
		SLC12A5	chr20_46009497_T_C_b38
		SLC12A5	chr20_46009497_T_C_b38
		SLC12A5	chr20_46009497_T_C_b38
		SLC12A5	chr20_46009497_T_C_b38
20	rs17576	*SLC12A5*	chr20_46011586_A_G_b38
		SLC12A5	chr20_46011586_A_G_b38
		SLC12A5	chr20_46011586_A_G_b38
		SLC12A5	chr20_46011586_A_G_b38
20	rs3787268	*PLTP*	chr20_46013092_G_A_b38
		PLTP	chr20_46013092_G_A_b38
		PLTP	chr20_46013092_G_A_b38
20	rs2250889	*SLC12A5*	chr20_46013767_G_C_b38
20	rs17577	*SLC12A5*	chr20_46014472_G_A_b38
		CD40	chr20_46014472_G_A_b38
		CD40	chr20_46014472_G_A_b38
		CD40	chr20_46014472_G_A_b38
		CD40	chr20_46014472_G_A_b38
		CD40	chr20_46014472_G_A_b38
		SLC12A5	chr20_46014472_G_A_b38
		ACOT8	chr20_46014472_G_A_b38
		ACOT8	chr20_46014472_G_A_b38
		SLC12A5	chr20_46014472_G_A_b38

the *MMP* genes
exportal.org/)) (p <8.0E5. FDR≤0.05)

Phenotype Id	Intron Id	p
chr11:102832906:102833452:clu_16168:ENSG0(	102832906:102833452:clu_16168	0.000000000065
chr11:102832906:102833452:clu_16168:ENSG0(	102832906:102833452:clu_16168	7.5E-41
chr20:46126741:46128138:clu_33045:ENSG000(	46126741:46128138:clu_33045	0.0000000029
chr20:46126741:46128138:clu_32508:ENSG000(	46126741:46128138:clu_32508	0.0000000067
chr20:46126741:46128138:clu_32508:ENSG000(	46126741:46128138:clu_32508	0.0000000067
chr20:46021886:46023369:clu_29529:ENSG000(	46021886:46023369:clu_29529	0.0000000098
chr20:46126741:46128138:clu_27442:ENSG000(	46126741:46128138:clu_27442	0.00000041
chr20:45841956:45844263:clu_24540:ENSG000(	45841956:45844263:clu_24540	0.0000011
chr20:46126741:46128138:clu_22055:ENSG000(	46126741:46128138:clu_22055	0.0000015
chr20:45841956:45844263:clu_27123:ENSG000(	45841956:45844263:clu_27123	0.0000041
chr20:46021886:46023369:clu_29529:ENSG000(	46021886:46023369:clu_29529	0.000000000003
chr20:46021886:46023369:clu_53353:ENSG000(	46021886:46023369:clu_53353	0.0000000099
chr20:46023071:46023369:clu_24852:ENSG000(	46023071:46023369:clu_24852	0.00000029
chr20:46021886:46023369:clu_19068:ENSG000(	46021886:46023369:clu_19068	0.00000056
chr20:46023071:46023369:clu_25498:ENSG000(	46023071:46023369:clu_25498	0.00000078
chr20:46021886:46023369:clu_26648:ENSG000(	46021886:46023369:clu_26648	0.000002
chr20:46021886:46023369:clu_27001:ENSG000(	46021886:46023369:clu_27001	0.0000062
chr20:46021886:46023369:clu_29529:ENSG000(	46021886:46023369:clu_29529	0.0000000000093
chr20:46023071:46023369:clu_24852:ENSG000(	46023071:46023369:clu_24852	0.00000033
chr20:46021886:46023369:clu_26648:ENSG000(	46021886:46023369:clu_26648	0.000002
chr20:46021886:46023369:clu_53353:ENSG000(	46021886:46023369:clu_53353	0.000011
chr20:45907904:45909942:clu_30841:ENSG000(	45907904:45909942:clu_30841	0.00000016
chr20:45907904:45909942:clu_31248:ENSG000(	45907904:45909942:clu_31248	0.0000011
chr20:45907904:45909942:clu_31643:ENSG000(	45907904:45909942:clu_31643	0.0000096
chr20:46021886:46023369:clu_29529:ENSG000(	46021886:46023369:clu_29529	0.0000000047
chr20:46021886:46023369:clu_29529:ENSG000(	46021886:46023369:clu_29529	0.00000000011
chr20:46126741:46128138:clu_32508:ENSG000(	46126741:46128138:clu_32508	0.000000006
chr20:46126741:46128138:clu_32508:ENSG000(	46126741:46128138:clu_32508	0.000000006
chr20:46126741:46128138:clu_33045:ENSG000(	46126741:46128138:clu_33045	0.000000043
chr20:46126741:46128138:clu_27442:ENSG000(	46126741:46128138:clu_27442	0.00000038
chr20:46126741:46128138:clu_22055:ENSG000(	46126741:46128138:clu_22055	0.00000092
chr20:46023071:46023369:clu_25498:ENSG000(	46023071:46023369:clu_25498	0.0000012
chr20:45841956:45844263:clu_24540:ENSG000(	45841956:45844263:clu_24540	0.0000018
chr20:45841956:45844263:clu_27123:ENSG000(	45841956:45844263:clu_27123	0.0000021
chr20:46023071:46023369:clu_32502:ENSG000(	46023071:46023369:clu_32502	0.000017

Effect Size (β)	Tissue
-0.51	Testis
0.94	Testis
0.5	Thyroid
0.45	Lung
0.45	Lung
0.79	Pituitary
0.49	Artery - Aorta
0.58	Heart - Left Ventricle
0.74	Cells - EBV-transformed lymphocytes
0.49	Heart - Atrial Appendage
0.63	Pituitary
0.49	Testis
-0.44	Brain - Cortex
0.61	Brain - Substantia nigra
-0.32	Brain - Cerebellar Hemisphere
0.45	Brain - Cerebellum
0.45	Spleen
0.63	Pituitary
-0.45	Brain - Cortex
0.46	Brain - Cerebellum
0.38	Testis
0.36	Adipose - Subcutaneous
0.41	Breast - Mammary Tissue
0.32	Skin - Sun Exposed (Lower leg)
0.38	Pituitary
0.88	Pituitary
0.45	Lung
0.45	Lung
0.46	Thyroid
0.49	Artery - Aorta
0.76	Cells - EBV-transformed lymphocytes
-0.43	Brain - Cerebellar Hemisphere
0.56	Heart - Left Ventricle
0.49	Heart - Atrial Appendage
-0.41	Lung
